# Extractives from *Artemisia afra* with Anti-Bacterial and Anti-Fungal Properties

**DOI:** 10.3390/plants12193369

**Published:** 2023-09-25

**Authors:** Tumelo L. Molokoane, Douglas Kemboi, Xavier Siwe-Noundou, Ibukun M. Famuyide, Lyndy J. McGaw, Vuyelwa J. Tembu

**Affiliations:** 1 Department of Chemistry, Tshwane University of Technology, Private Bag X680, Pretoria 0001, South Africa; tumelolinahmolokoane@gmail.com; 2 Department of Chemistry, University of Kabianga, Kericho 2030, Kenya; 3 Department of Pharmaceutical Sciences, Sefako Makgatho Health Sciences University, Pretoria 0204, South Africa; xavier.siwenoundou@smu.ac.za; 4 Phytomedicine Programme, Department of Paraclinical Sciences, University of Pretoria, Private Bag X04, Onderstepoort 0110, South Africa; adeyerimi@gmail.com (I.M.F.); lyndy.mcgaw@up.ac.za (L.J.M.)

**Keywords:** *Artemisia afra*, extractives, anti-bacterial, anti-fungal, cytotoxicity

## Abstract

Secondary metabolites were isolated using chromatographic techniques after being extracted sequentially from the roots of *Artemisia afra* using organic solvents such as ethanol, ethyl acetate, dichloromethane, and n-hexane. The isolated compounds were evaluated for anti-fungal, anti-bacterial, and cytotoxicity activities. Spectroscopic techniques, including Nuclear Magnetic Resonance (NMR), Fourier transform infrared (FTIR), and liquid chromatography–mass spectrometry (LC-MS), were used to elucidate the structures of the isolated compounds. The phytochemical investigation of *A. afra* led to the isolation of eight (**A**–**H**) compounds which were identified as 3*β*-taraxerol (**A**), 3*β*-taraxerol acetate (**B**), dodecyl-*p*-coumarate (**C**), ferulic acid (**D**), scopoletin (**E**), sitosterol-3-*O*-*β*-*D*-glucopyranoside (**F**), 3,5-di-*O*-feruloylquinic acid (**G**) and Isofraxidin-7-*O*-*β*-*D*-glucopyranoside (**H**) based on spectroscopic data. Compounds **A**, **B**, **C**, **F**, **G**, and **H** are known but were isolated for the first time from the roots of *A. afra*. The isolated compounds and extracts from *A. afra* exhibited good anti-fungal and anti-bacterial activity with dichloromethane and ethyl acetate crude extracts (0.078 mg/mL) and compound **E** (62.5 µg/mL) showed good activities against *Escherichia coli*. Compounds **C** and **F** also showed good activity against *Enterococcus faecalis* with minimum inhibitory concentration (MIC) values of 62.5 and 31.25 µg/mL, respectively. Extracts and compounds (**A**–**H**) exhibited anti-fungal and anti-bacterial properties and showed no toxicity when tested on Vero monkey kidney (Vero) cells.

## 1. Introduction

*Artemisia afra*, also known as “African wormwood,” is a member of the Asteraceae family, and the genus includes between 200 and 400 species. Dry or fresh leaves, young stems, and the roots of *A. afra* are used traditionally for the treatment of illnesses such as intestinal worms, malaria, colic, fever, loss of appetite, and colds. The use of intravenous lines, catheters, and drains as indwelling medical devices contributes to many bacterial and fungal infections if not adequately sterilized. These tools allow the germs to enter the bloodstream by circumventing the mucosal surface’s physical barrier [1]. The World Health Organization (WHO) reported that *Escherichia coli’s* resistance rates to ciprofloxacin varied from 8.4 to 92.9%, and *Klebsiella pneumoniae’s* resistance rates ranged from 4.1 to 79.4%. According to the WHO, *Staphylococcus aureus’s* resistance towards methicillin was 12.11%, and for *E. coli,* resistance to third-generation cephalosporins was 36.0%. Drug-resistant *Candida albicans* ranked among the critical priority group of fungal pathogens. Even though there are excellent anti-fungal medications available, invasive candidiasis has a mortality rate of 20 to 50%. Depending on the underlying problems, hospital stays can extend up to two months and commonly last between two and four weeks [2,3]. This makes treating bacterial and fungal infections more difficult, more likely to fail, necessitating longer hospital stays and far more expensive treatment options. The prevalence of drug-resistant diseases, as well as the toxicity of currently available anti-fungal and anti-bacterial drugs, has raised awareness of natural products’ antimicrobial activity [4].

One of the several Ethiopian herbs used in the treatment of infectious diseases over the past ten years is *Artemisia* species, which has been used traditionally in a variety of populations. The traditional uses include treatment of syphilis, rabies, tonsillitis, cough, leprosy, and gonorrhea [5]. Fresh roots have been used to treat domestic animals with epilepsy. Additionally, boiled leaves and milk were reported to alleviate heart troubles, and leaves mixed with other plants are used for the treatment of malaria [6]. *Artemisia* species contain a diverse chemical composition, which include flavonoids, essential oils, terpenoids, sesquiterpene lactones, coumarins, phenolic acids, caffeoylquinic acids, and sterols [7,8].

According to previous reports, *A. afra* has a wide range of microorganism inhibitory properties [9]. *A. afra* demonstrated strong pharmacological properties, such as antioxidant, antimicrobial, spasmolytic action, and cardiovascular protection [10]. *A. afra* also inhibited several bacteria, fungi, and protozoa [11]. Six known compounds were isolated (scopoletin, acacetin, *α*-amyrin, phytol, betulinic acid, and 12*α*, 4*α*-dihydroxybishopsolicepolide) from *A. afra* and were evaluated for their antimicrobial activity against *Actinomyces israelii, Streptococcus mutans*, *Prevotella intermedia, Aggregatibacter actinomycetemcomitans, Porphyromonas gingivalis*, and *C. albicans*. It was established that the isolated compounds showed activities ranging from 1.0 to 0.25 mg/mL, while the crude extracts’ activities ranged from 1.6 to 25 mg/mL [12]. Isoalantolactone, a sesquiterpenoid from *A. afra*, was tested against HeLa cancer cells, and it showed dose-dependent cytotoxicity with a low IC_50_ value of 8.15 ± 1.16 μM [13]. Artemisinin isolated from *A. annua* L. reduced the growth of three bacterial strains (*Salmonella* sp., *S. aureus*, and *B. subtilis*) with a MIC value of 0.09 mg/mL against all strains [14]. Yomogiartemin and 1*α*, 4*α*-dihydroxybishopsolicepolide, two guaianolide sesquiterpene lactones, were previously isolated and were active (IC_50_ < 10 μg/mL; ~10 μM) against intra-erythrocytic and gametocyte asexual *P. falciparum* parasites with IC_50_ values of <10 μg/mL and ~10 μM respectively [15]. Since only a few publications have been published since 2009 in terms of phytochemical identification in *A. afra*, this project focused on the isolation, characterization, and evaluation of the anti-bacterial properties of secondary metabolites from the roots of the *Artemisia afra* plant.

## 2. Results and Discussions

### 2.1. Isolated Compounds from A. afra

**Figure 1:**
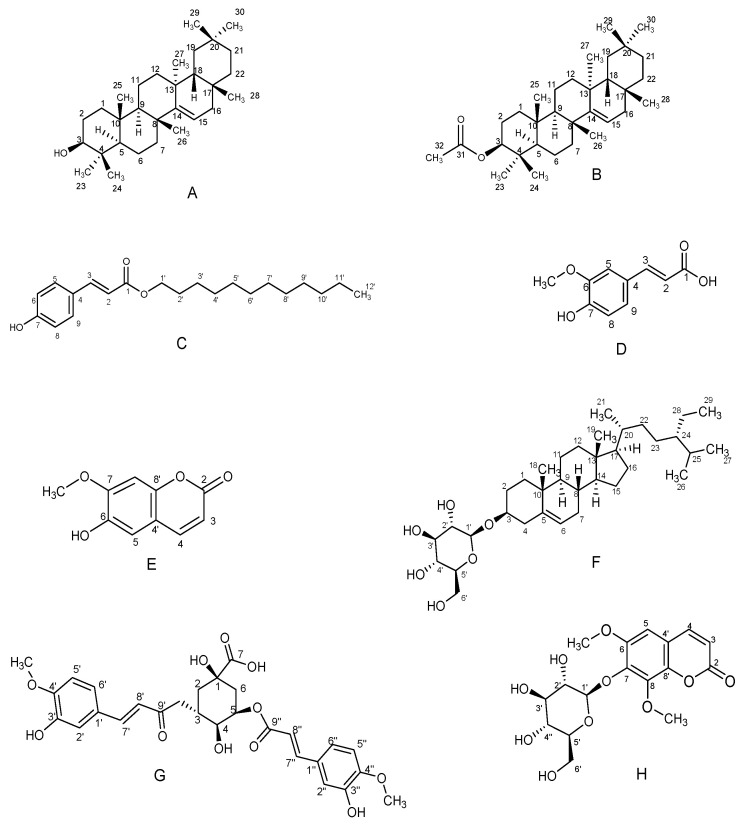
Structures of compounds isolated from *A. afra* roots (**A**–**H**).

Chromatographic separation of the EtOAc/DCM and EtOH *A. afra* roots crude extracts afforded eight known compounds. EtOAc and DCM crude extracts were combined based on the similarity of their thin-layer chromatography (TLC) profiles. Compounds (**A**–**H**) were identified using detailed spectroscopic analysis and by comparison of their spectroscopic data with those reported in the literature as 3*β*-taraxerol (**A**) [16,17,18], 3*β*-taraxerol acetate (**B**) [17,19], dodecyl-*p*-coumarate (**C**) [20], ferulic acid (**D**) [21], and scopoletin (**E**) [22,23,24], isolated from the EtOAc, DCM extracts and, sitosterol-3-*O*-*β*-*D*-glucopyranoside (**F**) [24,25,26], 3,5-di-*O*-feruloylquinic acid (**G**) [27], and isofraxidin-7-*O*-*β*-*D*-glucopyranoside (**H**) [28], isolated from the ethanol extracts. Figure 1 below shows the structures of the isolated compounds.

According to Bora and Sharma [7], the genus *Artemisia* is characterized by terpenoids, flavonoids, coumarins, caffeoylquinic acids, sterols, and acetylenes as its major classes of phytoconstituents of the genus. In the present study, eight compounds (**A**–**H**) were isolated and identified from the roots of *A. afra*, which were classified as triterpenes (**A** and **B**), an aryl coumarate (**C**), a hydroxycinnamic acid (**D**), coumarins (**E** and **H**), a sterol (**F**), and a quinic acid (**G**). All eight isolated compounds are known; however, six compounds (**A**, **B**, **C**, **F**, **G**, and **H**) were isolated for the 1st time from the roots of *A. afra*. Most of the isolated compounds in this research were phenolic compounds, which supports previous studies conducted on extracts and/or pure compounds of the *Artemisia* species, which revealed high phenolic content, antioxidant capacity, and anti-inflammatory activity [29]. According to Balasundram et al. [29], the phenolic compounds found in fruits and vegetables have a variety of biological properties, including anti-inflammatory, antiatherogenic, anti-bacterial, antithrombotic, and cardioprotective, but especially antioxidant activity.

### 2.2. Anti-Bacterial, Anti-Fungal, and Cytotoxicity Studies of A. afra Crude Extracts and Isolated Compounds

According to Bora and Sharma [7], a review of the literature revealed that the various *Artemisia* species have a wide range of biological activities, including antimalarial, cytotoxic, antihepatotoxic, anti-bacterial, anti-fungal, and antioxidant activity. Some very important drug leads have been discovered from this genus, notably artemisinin, the well-known antimalarial drug isolated from the Chinese herb *Artemisia annua* [7].

The anti-bacterial, anti-fungal, and cytotoxicity results of *Artemisia afra* crude extracts and isolated compounds (**A**–**H**) are given in Table 1 and Table 2. In this study, the MIC values of the crude extracts and compounds **A**, **C**, **D**, **E**, **F**, **G,** and **H** were determined using a serial dilution method as described by Masoko et al. [30] and Eloff [31]. Values were recorded in mg/mL. Compound **B** was not soluble in both solvents used (acetone and DMSO); therefore, antimicrobial assays were not achieved for compound **B**. From the results obtained (Table 1), the crude extracts showed anti-bacterial activities ranging from 0.078 to 2.5 mg/mL and no activity for other compounds. Sartorrato et al. [32] mentioned that MIC values of 0.5 or less than 0.5 mg/mL are strong microbial inhibitors; this gives a clear indication that the EtOAc and DCM crude extract are strong inhibitors of *Escherichia coli* with a MIC value of 0.078 mg/mL followed by EtOH crude with a moderate MIC value of 0.625 mg/mL and the hexane crude showed no activity. All crude extracts showed strong inhibition against *Enterococcus faecalis* with an average MIC value of 0.156 mg/mL. Hexane and EtOH extracts showed moderate activity against *Pseudomonas aeruginosa* with MIC values of 1.25 and 2.5 mg/mL, respectively, while the EtOAc and DCM extracts showed no activity. EtOAc, DCM, and EtOH extracts showed moderate activity against *Staphylococcus aureus* and *Salmonella* Typhimurium with a range of 0.625–1.25 mg/mL, while the hexane crude showed no activity for the two bacteria strains with a MIC value above 250 mg/mL. All the crude extracts showed moderate fungal activity against *C. albicans*, with a range of 0.625 to 1.25 mg/mL. The cytotoxicity study of the crude extracts as a mixture of different compounds has shown to be non-toxic with LC_50_ values ranging from 50 to 380 µg/mL. A crude extract of a medicinal plant is deemed safe according to standards set by the National Cancer Institute (NCI) Plant Screening Program if in vitro cytotoxicity experiments shows an IC_50_ value greater than 20 g/mL after 48–72 h of incubation [33].

Screening of isolated compounds against selected bacterial and fungal strains (Table 2) showed selective anti-bacterial and anti-fungal activity ranging from 31.25–250 µg/mL, with others showing no activity. Taraxerol (**A**), scopoletin (**E**), and isofraxidin-7-*O*-*β*-*D*-glucopyranoside (**H**) showed activity against *Escherichia coli* with scopoletin isolated from the EtOAc and DCM crude showing the highest MIC value of 62.5 µg/mL. Dodecyl-*p*-coumarate (**C**), scopoletin, and sitosterol-3-*O*-*β*-*D*-glucopyranoside (**F**) also showed strong to moderate activity against *Enterococcus faecalis* with sitosterol-3-*O*-*β*-*D*-glucopyranoside from the EtOH crude showing the lowest MIC value of 31.25 µg/mL. Compounds **A**, **D**, **E,** and **H** showed activity against *Staphylococcus aureus* at their highest concentrations (250 µg/mL). Taraxerol, scopoletin, and isofraxidin-7-*O*-β-*D*-glucopyranoside showed moderate activity against *S.* Typhimurium*,* with scopoletin showing the highest activity with a MIC value of 125 µg/mL. All the compounds showed no activity against the Gram-negative *Pseudomonas aeruginosa* at their highest concentration (250 µg/mL), showing that this bacterium is resistant to the isolated compounds. All isolated compounds also showed anti-fungal activity against *C. albicans* at their highest concentrations (250 µg/mL) except for compound **G**, which showed no activity against all bacterial and fungal strains selected. The cytotoxicity study of the isolated compounds demonstrated that they were non-toxic with LC_50_ values above 200 µg/mL.

The selectivity index (SI) is a value that shows the selective toxicity of an extract or compound to the tested microbes compared to mammalian cells. The higher the SI value, the safer the extract/compound [34]. Therefore, a SI value greater than 1 shows that tested samples are less toxic to normal mammalian cells than to the pathogen. In the present study, the hexane crude extract had the best SI of 2 against *E. faecalis*. Of the compounds tested, sitosterol-3-*O*-*β*-*D*-glucopyranoside had the best SI of more than 6 against *E. faecalis*, while scopoletin, with an SI of 3, was best against *E. coli*. Both compounds are potential candidates for further evaluation of their therapeutic potential against pathogens. It may also be worthwhile to investigate if the most active compounds will have synergistic activity against microbes.

The MIC values of 3*β*-taraxerol (**A**) in this study were above 250 µg/mL, indicating that this compound’s antimicrobial activity was negligible against the tested microorganisms. However, from previous studies, taraxerol was found to have antimicrobial activity against *S. aureus* (18.37–23.63 mm inhibition zone), *P. aeruginosa* (18.23–21.30 mm inhibition zone), and no activity against *C. albicans* [35]. Koay et al. [36] investigated the minimum inhibitory concentrations (MICs) of taraxerol on several bacteria and found that the compound is active against Gram-positive *Bacillus subtilis* and *Staphylococcus aureus* at a concentration of 15.6 µg/mL but is only moderately inhibitive to the Gram-negative *Escherichia coli*, *Klebsiella pneumoniae*, and *Salmonella* Typhimurium at a concentration of 62.5 µg/mL. The antimicrobial activity of taraxerol is comparable to that of positive control gentamicin [37]. In an earlier study, taraxerol at a concentration of 1 mg/disc exhibited weak anti-fungal activities against four types of fungi, namely *Aspergillus niger*, *Aspergillus flavus*, *Rhizoctonia phaseoli*, and *Penicillium chrysogenum* [38]. Taraxerol was particularly effective against several species of *Trichophyton*, for instance, *T. rubrum* and *T. mentagrophytes,* with a MIC value of 12.5 µg/mL, as well as *Candida albicans* (MIC = 25 µg/mL) and *Aspergillus niger* at 100 µg/mL [39,40]. Singh et al. [41] observed that 1 mg of taraxerol compound exhibited moderate antimicrobial activity against two Gram-positive (*Staphylococcus aureus* and *Bacillus thuringiensis*) and three Gram-negative bacteria (*Escherichia coli, Enterobacter cloacae*, and *Klebsiella pneumoniae*) [41]. The cytotoxicity study of taraxerol on Vero cells was above 200 µg/mL. Taraxerol in previous studies showed cytotoxicity towards the A431 squamous carcinoma cell line at 2.65 µg/mL, even though it was found to be inactive against HeLa, MCF-7, and MRC-5 cancer cell lines. While taraxerol cytotoxicity exhibited low activity compared to the positive control, doxorubicin, the activity is comparable to that of cisplatin [40,42]. Microbial studies were not conducted on compound (**B**) as it was not soluble in acetone and DMSO; however, previous research found that the COX-1 and COX-2 enzymes were significantly inhibited by 3*β*-taraxerol acetate (IC_50_: 116.3 +/− 0.03 µM and IC_50_: 94.7 +/− 0.02 µM, respectively) when using an in vitro enzyme inhibition assay [43]. Cyclooxygenase inhibitors are one of the main classes of therapeutic agents for the management of inflammation [44]. Taraxerol acetate was also found to have gastroprotective effects [45], anticancer [46], antiviral [47], antiulcer [48], anti-inflammatory, antimicrobial [41], anti-leukemic activity [49], and antipyretic effects [38].

Dodecyl-*p*-coumarate (**C**) in this study showed good activity against *E. faecalis* with a MIC value of 62.5 mg/mL. This compound showed no activity against other tested microbes and insignificant activity with *C. albicans*. From previous studies, compound **C** showed inhibition of mycelium growth of two pathogens, *Botrytis cinerea* and *Sclerotinia sclerotiorum* [50]. Dodecyl-*p*-coumarate also displayed high leishmanicidal activity (16.34 ± 0.49, *Leishmania braziliensis* amastigotes, EC_50_ µg/mL), likely due to their relatively high lipophilicity and antiplasmodial activity (81.19 ± 8.54 *Plasmodium falciparum*, EC_50_ µg/mL) [51]. According to cytotoxicity research, this compound had a minimal toxic effect on HEK295 but reduced the viability of MCF-7 tumor cells by at least 23% at concentrations of 1 µg/mL and 15% at a concentration of 5 μg/mL for Caco-2 tumor cells [52].

Ferulic acid (**D**) is found in Chinese medicinal herbs such as *Ligusticum chuangxiong, Cimicifuga heracleifolia*, and *Angelica sinensis*. Ferulic acid showed insignificant activity against all tested microbes with a MIC value equal to 250 µg/mL and above. Ferulic acid is used in cosmetic and food industries due to its low toxicity and biological processes as the raw material to produce vanillin and preservatives [53,54]. It was found that ferulic acid had antimicrobial activity with a MIC value of 100 μg/mL for both *E. coli* and *P. aeruginosa* and 1100 μg/mL and 1250 μg/mL for *S. aureus* and *L. monocytogenes*, respectively [55]. Ferulic acid from Indonesian purple rice showed biological function as an antimicrobial agent through toll-like receptor signaling [56]. Compound **D** was also reported to have anticancer effects [57].

Scopoletin (**E**) is a significant phytoalexin that has been identified from numerous plants and classified as a phenolic coumarin [58]. The compound and the plants that produce it have been used to treat symptoms and disorders, including inflammation, convulsions, leprosy, and rheumatic pains [59,60,61]. Scopoletin, in this study, showed activity against *S.* Typhimurium (125 µg/mL) and *E. coli* (62.5 µg/mL) and was not active against other selected microbes, including *C. albicans*. Buathong et al. [62] reported that scopoletin showed high anti-bacterial activity and can inhibit both Gram-positive bacteria, such as *Staphylococcus aureus* ATCC 43300 and *Enterococcus faecium* UCLA 192, with a minimum inhibitory concentration (MIC) value of 128 μg/mL, and Gram-negative bacteria *Stenotrophomonas maltophila* DMST 19079 with a MIC value of 256 μg/mL. The literature review of the pharmacological properties of scopoletin revealed that scopoletin possesses antimicrobial activity, antioxidant activity, immunomodulatory and anti-inflammatory activity, antimetabolic disorder, and neuroprotective activity [63]. Scopoletin isolated from *Artemisia afra* was reported to cause a strong transformation of the DPPH radical into its reduced form, with an IC_50_ value of 1.24 µg/mL that was comparable to that of vitamin C (1.22 µg/mL), and it was ineffective against *Candida albicans* [12]. Mogana et al. [64] also reported that the EC_50_ of scopoletin for scavenging DPPH and ABTS free radicals were 647.89 ± 0.07 µM and 191.51 ± 0.01 µM, respectively.

Sitosterol-3-*O*-*β*-*D*-glucopyranoside (**F**), in this study, showed good activity against *Enterococcus faecalis* (MIC 31.25 μg/mL) and was not active with all other tested microbes. Nevertheless, according to Subramaniam et al. [65], this compound had acceptable activity against *S. dysenteriae*, *P. aeruginosa,* and *E. faecalis* with MIC values of 12, 10, and 15 g/mL, respectively [66]. Njinga et al. [67] reported that sitosterol-3-*O*-*β*-*D*-glucopyranoside isolated from *Lannea kerstingii* showed a wide spectrum of anti-bacterial and anti-fungal activity at a concentration of 200µg/mL against *S. aureus*, methicillin-resistant *Staphylococcus aureus* (MRSA), *Proteus mirabilis*, *S. typhi*, *K. pneumoniae*, *E. coli*, *Bacillus subtilis* and was also active against the fungi *C. albicans* and *C. tropicalis*. Regarding anticancer activity, compound (**F**) was found to exhibit cytotoxic effects against HepG2 and Huh7 cells but not against normal human primary fibroblasts. Compound (**F**) was able to inhibit the proliferation of HepG2 and Huh7 cells in a dose-dependent manner with half-maximal inhibitory concentration (IC_50_) values of 4.64 ± 0.48 µg/mL and 5.25 ± 0.14 µg/mL, respectively (*p* < 0.01) [66].

Compound (**G**), 3, 5-di-*O*-feruloylquinic acid, was reported for the first time from the roots of *Artemisia afra*. This compound’s antimicrobial study of the selected strains is reported for the first time. However, no activity was observed against all tested microorganisms with MIC values > 250 µg/mL.

Isofraxidin-7-*O*-*β*-*D*-glucopyranoside (**H**), also known as calycanthoside, a known glucoside of isofraxidin classified as a coumarin and reported for the first time from the roots of *A. afra*, showed insignificant activity against all tested microorganisms with a MIC of 250 µg/mL and exceeding. Yuan et al. [68] also tested the anti-bacterial effect of calycanthoside against *Staphylococcus aureus* by filter paper slice method, and no activity was observed. However, isofraxidin-7-*O*-*β*-*D*-glucopyranoside isolated from *Artemisia selengensis* showed 14% inhibitory activity against IL-6 production in TNF-α stimulated MG-63 cells [28].

## 3. Materials and Methods

The sequential extraction method was carried out using ethanol (EtOH), ethyl acetate (EtOAc), dichloromethane (DCM), and hexane. Column chromatography (CC) over silica gel (Kieselgel 60 GF_254_, 15 μm, Merck, Germany) was used for fractional, elution, and separation of compounds from crude extracts. Thin layer chromatography (aluminum sheets covered with silica gel 60 F_254_ from Merck) was used for the analysis of compound profiles and determination of R_f_ (retention factor) values. Ultra-violet (UV) light at 254 nm and 365 nm was used to identify ultra-violet active constituents. The TLC plates were stained using *p*-anisaldehyde and sulphuric reagent and heated to about 100°C for two minutes to identify non-UV compounds.

The melting point values were determined using an Ernst Leitz Wetziar micro-hot stage and were reported in degrees Celsius. Perkin Elmer version 10.5.4 was used for FTIR analysis, and samples were evaluated as function groups in their natural state, with absorption bands quantified in cm^−1^.

The Jasco P-2000 polarimeter was used to carry out the specified optical rotation, [α]_D_ (JASCO, Tokyo, Japan). The polarimeter was calibrated using a D-glucose solution of 10 g/100 mL at mercury green line λ = 546 nm at 22.0 °C. Both Chloroform (CHCl_3_) and dimethyl sulfoxide (DMSO) were used to dissolve between 1 and 10 mg of the pure compound, and optical rotation in both the clockwise and counterclockwise directions was measured while the temperature ranged between 22 and 25 °C. Six analyses were conducted, and the average optical rotation, measured in [(^0^). mL dm^−1^ g^−1^] degree milliliters per decimeter grams, was reported.

To identify the molecular ion of the compounds, HR-ESI-MS (high-resolution electron spray ionization mass spectroscopy) investigations were performed on a Bruker Daltonics Compact QTOF mass spectrometer (Milford, CT, USA) in positive mode using an electrospray ionization probe. The spectrometer was connected to a thermal scientific ultimate 3000 Dionex UHPLC system, which included an HPG-3400 RS Pump, a WPS-3000 RS Auto Sampler, and a DAD-3000 RS detector. The 5 min run was attained using an Acclaim RSLC 120 C18, 2.2 μm, 2.1 × 100 mm (P/N 068982) column at 40 °C, and a flow rate of 0.2 mL/min. The solvent system employed was Water-Acetonitrile (10:90, *v*/*v*), each solvent containing 0.1% of formic acid.

The one-dimension (1D) and two-dimension (2D) NMR spectra were acquired by 400 MHz (100.6 MHz for ^13^C NMR) Bruker Avance III HD and Varian spectrometers. The spectra were recorded at 25 °C, and the chemical shifts (δ) were calculated in parts per million (ppm) and compared to the internal solvent shift of tetramethyl silane (TMS). The chemical shifts were measured in ppm relation to residual chloroform (δ 7.26 and δ 76.29 ppm), methanol (δ 3.30, 4.82 and δ 48.02 ppm) and dimethyl sulfoxide (δ 2.48 and δ 39.53 ppm). Coupling constants were recorded in hertz (Hz). Deuterated chloroform (CDCl_3_), deuterated methanol (CD_3_OH), and deuterated dimethyl sulfoxide solvents, all purchased from Sigma-Aldrich, Kempton park, RSA, were used to dissolve the NMR samples.

### 3.1. Collection and Identification

**Figure 2:**
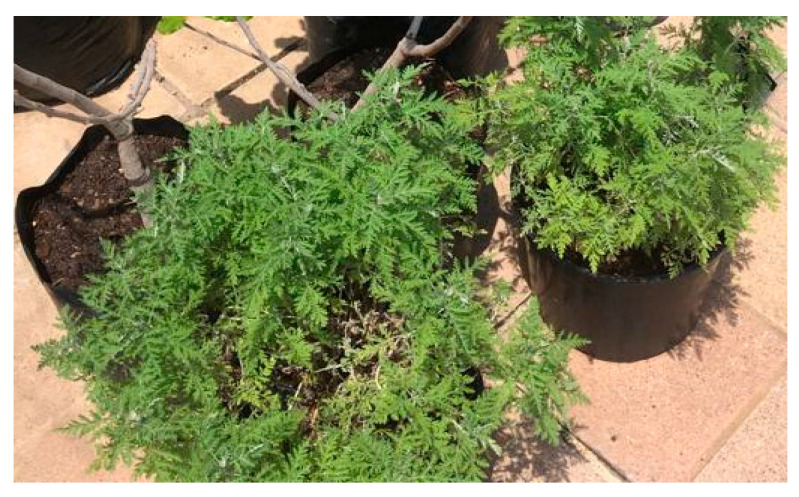
*Artemisia afra* plants.

*Artemisia afra* plants (Figure 2) were purchased from Grow Wild in November 2018, and a voucher specimen (Voucher number: 19034) was sent and identified at the South African National Biodiversity Institute (SANBI) Pretoria as *Artemisia afra* Jacq. ex Willd.

### 3.2. Sample Preparation and Extraction

*Artemisia afra* roots were cut into small pieces and air-dried. Before extraction, the dried plant material was ground into powder using a laboratory ball mill (MM200, Retsch, Germany) and the powder was stored at room temperature in tight plastic bags away from sunlight. The roots ground material (3.79 kg) was sequentially extracted in five litres of organic solvents: hexane, dichloromethane, ethyl acetate, and ethanol at 100% each, respectively, using a shaker (HS 501 digital, IKA-Werke, Staufen, Germany) for 4 days per solvent. The extractives were filtered using Whatman No. 1 filter paper and evaporated at low pressure at approximately 40 °C using a rotavapor. After that, the concentrated crude extracts were dried, weighed, and refrigerated until fractionation.

### 3.3. Fractionation and Purification of Crude Extracts

#### 3.3.1. Fractionation and Purification of DCM/Ethyl Acetate Crude Extracts

The DCM and EtOAc crude extracts were combined based on the similarity of their TLC profile and fractionated using column chromatography (CC) on 60-120 mesh silica gels (800 g) packed with hexane. A total amount of 15 g of the combined DCM and EtOAc crude extracts was made into a free-flowing powder using 30 g of silica gel and was subsequently loaded onto an already packed column and eluted using solvent mobile phase with increasing step gradient of DCM in n-hexane (0–100%) of increasing concentration. A total of 217 fractions of 80 mL portions were collected. The fractions were spotted on a TLC plate, developed, and examined for UV active compounds at 254 and 365 nm wavelengths. To observe non-UV active compounds, the TLC plate was stained with a *p*-anisaldehyde/sulphuric acid spray reagent and heated to about 100 °C for two minutes. About 217 fractions were collected, and similar fractions observed from the TLC were combined to form six (6) sub-fractions labeled A-R (A, BCD, EF, I, L, and NOPQR). The fractions were purified further based on their TLC profiles. Further purification of fraction BCD (1916.1 mg) was carried out with elution achieved using 80:20 Hex: DCM, to obtain a colourless crystalline 3*β*-Taraxerol (**A**), 205.21 mg, 30:70 Hex: DCM to obtain a colourless crystalline 3*β*-Taraxerol acetate (**B**), 56.0 mg and 20:80 Hex: DCM to obtain a cream white powder Dodecyl-*p*- coumarate (**C**), 62.3 mg. Fraction L (238.4 mg) was further purified using column chromatography and elution was achieved using 100% CHCl_3_, to obtain a colourless crystalline Ferulic acid (**D**), 18.6 mg. Fraction NOPQRS (1500.7 mg) was further purified using column chromatography and elution achieved using 10:90 EtOAc: DCM, followed by 5:95 EtOAc: DCM (TLC prep) to obtain a yellow crystalline Scopoletin (**E**), 29.2 mg. Hexane and 60-120 mesh silica gels were used to pack the column for all purifications (stationary phase).

#### 3.3.2. Fractionation and Purification of Ethanol Crude Extract

Column chromatography was packed as described in Section 3.3.1, similar to that of the DCM and EtOAc crude. A total amount of 20 g of ethanol extract was introduced onto a packed column for elution. Fractions were collected using different solvent systems of increasing polarity. This yielded a total of 156 fractions of 80 mL, each labeled (1-207). Fractions that had similar compounds were combined into 6 main fractions labeled EtOH 5-8, EtOH 17-19, EtOH 28-31, EtOH 45-108, EtOH 121-134, and EtOH 148-156, respectively. Fraction EtOH 45-108 (56.14 mg) was further purified using column chromatography and elution achieved using 90:10 EtOAc: MeOH, to obtain green solids, 3,5-di-*O*-feruloylquinic acid (**F**), 19.6 mg. Fractions EtOH 121-134 upon drying formed layers that were further separated by washing off impurities with DCM and EtOAc. This yielded white powder, compound (**G**), 44.40 mg. Fraction EtOH 148-156 upon drying formed layers that were further separated by washing off impurities with CHCl_3_. This yielded pale brown powder, compound (**H**), 61.512 mg.

Repeated CC of *A. afra* (ethyl acetate, dichloromethane, and ethanol extracts) led to the isolation of 8 pure compounds (**A**–**H**), 3*β*-Taraxerol (**A**), 3*β*-Taraxerol acetate (**B**), Dodecyl-*p*-coumarate (**C**), Ferulic acid (**D**), Scopoletin (**E**), sitosterol-3-*O*-*β*-*D*-glucopyranoside (**F**), 3,5-di-*O*-feruloylquinic acid (**G**) and Isofraxidin-7-*O*-*β*-*D*-glucopyranoside (**H**).

### 3.4. Spectral Data of Isolated Compounds

#### 3.4.1. Compound A: 3*β*-Taraxerol

Colourless needle-like crystals (56.0 mg), Rf: 0.75 (n-hexane–EtOAc, 80:20), melting Point: 284–286 °C, [α]_D_^20^ = +14.2°, c = 0.25 in CHCl_3,_ HR-EI-MS [M + H]^+^: found *m/z* 427.8067 for C_30_H_50_O (calcd 426.7174), C_30_H_50_O, IR (KBr) Cm^–1^: 3490 (O-H), 3056, 1038, 996.9 (=C-H), 2920 and 2852, (aliphatic C-H) and 1446 (C=C). ^1^H NMR (400 MHz, CDCl_3_): 0.78 (1H, m, H-5), 0.83 (3H, s, H-24), 0.85 (3H, s, H-28), 0.92 (3H, s, H-30), 0.92 (1H, m, H-1), 0.94 (3H, s, H-27), 0.95 (1H, m, H-18), 0.95 (3H, s, H-25), 0.97 (1H, m, H-19), 0.97 (3H, s, H-29), 1.00 (3H, s, H-23), 1.02 (1H, m, H-22), 1.11 (3H, s, H-26), 1.25 (1H, m, H-21), 1.30 (1H, m, H-19), 1.31 (1H, m, H-21), 1.36 (1H, m, H-7), 1.38 (1H, m, H-22), 1.44 (1H, m, H-9), 1.45 (1H, m, H-11), 1.52 (1H, m, H-6), 1.56 (1H, m, H-12), 1.61 (2H, m, H-2), 1.63 (1H, m, H-1), 1.64 (1H, m, H-12), 1.66 (1H, m, H-16), 1.67 (1H, m, H-6), 1.70 (1H, m, H-11), 1.97 (1H, dd, J = 14.7, 3.2 Hz, H-16), 2.03 (1H, dt, J = 12.5, 3.0 Hz, H-7), 3.20 (1H, dd, J = 11.1, 4.8 Hz, H-3), 5.55 (1H, dd, J = 8.2, 3.2 Hz, H-15); ^13^C NMR (100.6 MHz, CDCl_3_): 15.4 (C-25), 15.5 (C-24), 17.5 (C-11), 18.8 (C-6), 21.3 (C-30), 25.9 (C-26), 27.2 (C-2), 28.0 (C-23), 28.8 (C-20), 29.8 (C-28), 29.9 (C-27), 33.1 (C-21), 33.4 (C-29), 33.7 (C-12), 35.1 (C-22), 35.8 (C-17), 36.7 (C-19), 37.6 (C-13), 37.7 (C-1), 37.7 (C-16), 38.0 (C-10), 38.8 (C-4), 39.0 (C-8), 41.3 (C-7), 49.3 (C-9), 48.8 (C-18), 55.5 (C-5), 79.1 (C-3), 116.9 (C-15), 158.1 (C-14). Supplementary data (Figures SA.1–SA.9).

#### 3.4.2. Compound B: 3*β*-Taraxerol Acetate

Colourless needle-like crystal (217.26 mg), Rf: 0.97 (n-hexane–EtOAc, 80:20), melting Point: 303–305 °C, [α]_D_^20^ = +62.7°, c = 0.23 in CHCl_3_, HR-EI-MS [M + H]: found *m/z* 469.8088 for C_32_H_52_O_2_ (calcd 468.7541), IR (KBr) cm^–1^: 3056, 1029.4, 991 (=C-H), 2938, 2870, 1475, 1375 (Aliphatic C-H), 1726.6 (C=O) and 1446 (C=C). ^1^H NMR (400 MHz, CDCl_3_): δH 0.85 (3H, s, H-28), 0.88 (3H, s, H-23), 0.92 (3H, s, H-27), 0.92 (3H, s, H-30), 0.92 (1H, m, H-5), 0.93 (1H, m, H-18), 0.97 (3H, s, H-25), 0.97 (3H, s, H-24), 0.97 (3H, s, H-29), 1.02 (1H, m, H-21), 1.06 (1H, m, H-22), 1.10 (1H, m, H-22), 1.11 (3H, s, H-26), 1.25 (1H, m, H-7), 1.92 (1H, m, H-1), 1.97 (1H, m, H-1), 1.30 (1H, m, H-12), 1.34 (1H, m, H-12), 1.36 (1H, m, H-7), 1.38 (1H, m, H-19), 1.42 (1H, m, H-19), 1.42 (1H, m, H-21), 1.47 (1H, m, H-9), 1.47 (1H, m, H-11), 1.61 (1H, m, H-6), 1.64 (1H, m, H-16), 1.64 (2H, m, H-2), 1.66 (1H, m, H-6), 1.67 (1H, m, H-11), 1.67 (1H, m, H-16), 2.06 (3H, s, CH3 COO), 4.47 (1H, dd, J = 10.2, 5. 6 Hz, H-3), 5.55 (1H, dd, J = 7.6, 2.4 Hz, H-15); ^13^C NMR (100.6 MHz, CDCl_3_): 15.5 (C-25), 16.6 (C-24), 17.5 (C-11), 18.7 (C-6), 21.3 (CH3 COO), 21.3 (C-30), 23.5 (C-2), 25.9 (C-26), 28.0 (C-23), 28.8 (C-20), 29.8 (C-28), 29.9 (C-27), 33.1 (C-7), 33.4 (C-29), 33.7 (C-16), 35.1 (C-21), 35.8 (C-17), 36.7 (C-12), 37.4 (C-22), 37.4 (C-10), 37.7 (C-1), 37.6 (C-13), 37.9 (C-4), 39.0 (C-8), 41.2 (C-19), 48.8 (C-18), 49.2 (C-9), 55.6 (C-5), 81.0 (C-3), 117.0 (C-15), 158.0 (C-14), 170.9 (COO). Supplementary data (Figures SB.10–SB.18).

#### 3.4.3. Compound C: Dodecyl-*p*-coumarate

Cream white powder (62.3 mg), Rf: 0.95 (n-hexane–EtOAc, 20:80), melting Point: 74–76 °C, [α]_D_^20^ = +14.2°, c = 0.56 in CHCl_3_, HR-EI-MS [M + H]: found *m/z* 333.6103 for C_21_H_32_O_3_ (calcd 332.4770), IR (KBr) cm^–1^: 3385.1 (OH stretch), 2922-2847 (C-H) 1677 (C=O), and 1606 & 1516 (Aromatic CH=CH). ^1^H–NMR (CDCl_3_, 300 MHz): δ 7.63 (1H, d, J = 15.9 Hz, H–3), 7.43 (2H, d, J = 8.4 Hz, H–5,9), 6.87 (2H, d, J = 8.4 Hz, H–6,8), 6.34 (1H, d, J = 15.9 Hz, H–2), 4.22 (2H, t, J = 6.6 Hz, H–1′), 1.72 (2H, m, H–2′), 1.41–1.28 (18H, br s, H–3′-H–11′), 0.91 (3H, t, J = 6.5 Hz, H–12′); ^13^C–NMR (CDCl_3_, 75 MHz); δ 168.0 (C–1), 158.0 (C–7), 144.6 (C–3), 130.0 (C–5,9), 127.0 (C–4), 115.5 (C–2), 115.9 (C–6,8), 64.8 (C–1′), 32.0 (C–10′), 29.72–26.0 (29.72, 29.68, 29.62, 29.56, 29.38, 29.32, 28.8, 26.0) (C–2′–C–9′), 22.7 (C–11′), 14.1 (C–12′). Supplementary data (Figures SC.19–SC.27).

#### 3.4.4. Compound D: Ferulic Acid

White crystals (18.6 mg), Rf: 0.45 (CDCl_3_: methanol 96:4), melting point: 169-171 °C, [α]_D_^21^ = −183.9°, c = 0.070 in CHCl_3_, HR-EI-MS [M + H]: found *m/z* 195.1823 for C_10_H_10_O_4_ (calcd 194.1840), IR (KBr) cm^–1^: 3333.8 (carboxylic acid OH stretch), 2946.8 & 2937.5 and 1611.4 (CH=CH), and 1614.4 & 1511 (Aromatic CH=CH). ^1^HNMR (400 MHz, CDCl_3_, J in Hz) δ 3.95 (3H, s, H-6′), 6.34 (1H, d, J = 15.9 Hz, H-2), 6.88 (1H, d, J = 8.2 Hz, H-5), 7.16 (1H, s, H-8), 7.60 (1H, d, J = 15.9 Hz, H-3), 7.43 (1H, d, J = 15 Hz, H-9); ^13^CNMR (100.6 MHz, CDCl_3_) δ: 56.0 (C-6′), 110.5 (C-5), 113.0 (C-8), 115.9 (C-2), 121.8 (C-9), 128.1 (C-4), 144.7 (C-3), 145.9 (C-7), 148.5 (C-6), 167.7 (C-1). Supplementary data (Figures SD.28–SD.36).

#### 3.4.5. Compound E: Scopoletin

Yellow crystals (40.037 mg), Rf: 0.55 (100% CDCl_3_), melting point: 204-207 °C, [α]_D_^20^ = +48.9°, c = 0.22 in CHCl_3_, HR-EI-MS [M + H]: found *m/z* 193.1408 for C_10_H_8_O_4_ (calcd 192.1681), IR (KBr) cm^–1^: 3333.8 (OH), 2991 & 2949.7 (C-H) 1703 (C=O) and 1611.4 (CH=CH), 1564.1 and 1514 benzene ring and 861.0. ^1^H NMR (400 MHz CDCl3): δ 3.88 (3H, s, OCH_3_-7), 6.19 (1H, s-OH-6), 6.28 (1H, d, J = 9.5 Hz, H-3), 6.87 (1H, s, H-5), and 7.60 (1H, d, J = 9.5 Hz, H-4); ^13^C NMR (100.6 MHz, CDCl_3_) δ 56.4 (O-CH_3_), 103.2 (C-8), 107.4 (C-5), 111.5 (C-4′), 113.4 (C-3), 143.4 (C-4), 144.0 (C-6), 149.7 (C-7), 150.3 (C-8′), and 161.5 (C-2). Supplementary data (Figures SE.37–SE.45).

#### 3.4.6. Compound F: Sitosterol-3-*O*-*β*-*D*-glucopyranoside

Solid (19.602 mg), melting point: 283-286 °C, [α]_D_^20^ = −1274.6°, c = 0.20 in DMSO, HR-EI-MS [M + H–H_2_O C_6_H_12_O_6_] ^+^: found *m/z* 399.0710 C_35_H_60_O_6_ (calcd 576.8473), FT-IR (cm^−1^) at 3370- OH stretch, 2934, 2870, 1659, 1456, (-CH (CH_3_) 2)-1377, secondary alcohol (C-OH)–1054. ^1^H NMR (400 MHz, DMSO-d6): δ 0.63 (3H, s, H-18), 0.79 (3H, d, J = 7.2 Hz, H-26), 0.80 (3H, t, J = 7 Hz, H-29), 0.88 (1H, m, H-9), 0.88 (3H, d, J = 6.4 Hz, H-21), 0.89 (1H, m, H-24), 0.94 (1H, m, H-1), 0.94 (3H, s, H-19), 0.97 (3H, d, J = 6.8 Hz, H-27), 0.99 (1H, m, H-15), 1.06 (lH, m, H-14), 1.08 (lH, m, H-17), 1.12-1.14 (2H, m, H-23), 1.19 (2H, m, H-28), 1.21 (2H, m, H-2, H-20), 1.23 (2H, m, H-16, H-22), 1.32 (1H, m, H-22), 1.37 (1H, m, H-8), 1.40 (1H, m, H-7), 1.45 (1H, m, H-22), 1.48 (2H, H-11), 1.51 (1H, m, H-15), 1.60 (lH, m, H-25), 1.62 (1H, m, H-2), 1.76 (1H, m, H-1), 1.79 (2H, m, H-16), 1.88 (1H, m, H-7), 1.92 (1H, m, H-12), 2.11 (1H, m, H-4), 2.33 (1H, m, H-4), 2.49 (1H, m, H-12), 3.09 (1H, m, H-3), 5.31 (1H, d, J = 4.8 Hz, H-6); glucose; 2.88 (lH, m, H-2′), 3.02 (lH, m, H-4′), 3.04 (lH, m, H-5′), 3.09 (lH, m, H-3′), 3.64 (1H, dd, J = 11.6, 5.6 Hz, H-6′), 3.64 (1H, dd, J = 11.6, 1.6 Hz, H-6′), 4.20 (1H, d, J = 7.6 Hz, H-1′); ^13^C NMR (100.6 MHz, DMSO-d6): δ 12.1 (C-29), 12.2 (C-18), 19.1 (C-21), 19.4 (C-19), 19.6 (C-26), 20.1 (C-27), 21.0 (C-11), 23.1 (C-28), 24.3 (C-15), 25.9 (C-23), 28.2 (C-16), 29.2 (C-25), 29.7 (C-2), 31.8 (C-8), 31.9 (C-7), 33.8 (C-22), 35.9 (C-20), 36.7 (C-10), 37.2 (C-1), 38.3 (C-4), 40.2 (C-12), 42.3 (C-13), 45.6 (C-24), 50.1 (C-9), 56.6 (C-14), 55.9 (C-17), 77.4 (C-3), 121.7 (C-6), 140.9 (C-5); glucose; 61.6 (C-6′), 70.6 (C-4′), 73.9 (C-2′), 77.1 (C-5′), 77.2 (C-3′), 101.2 (C-1′). Supplementary data (Figures SF.46–SF.53).

#### 3.4.7. Compound G: 3,5-Di-*O*-feruloylquinic Acid

Cream white powder (44.404), Rf: 0.20 (CDCl_3_: methanol, 55:45), melting point: 184–185 °C, [α]_D_^20^ = −86.0°, c = 0.21 in CHCl_3_, HR-EI-MS [M-Na+H_2_O]^−^: found *m/z* 553.1325 for C_27_H_28_O_12_ (calcd 544.5040), IR (KBr) cm^−1^: 3348 (OH), 2941 (C-H) 1685 (C=O) and 1606 (CH=CH), 1520, 1260 and 809 benzene ring. ^1^H NMR (400 CDCl_3_): Quinic acid; δ 2.03–2.36 (4H, m, H-2, H-6), 4.00 (1H, dd, J = 7.1, 3.0, H-4), 5.41 (1H, m, H-3), 5.46 (1H, ddd, J = 7.1, 7.1, 3.0, H-5), Feruloyl; δ 7.65–7.61 (each 1H, d, J = 15.9 Hz, H-7′ and H-7″), 7.09–7.08 (each 1H, H-2′ and H-2″), 6.97–6.96 (each 1H, d, J = 8.2 Hz, H-6′ and H-6″), 6.81 (each 1H, d, J = 8.2 Hz, H-5′ and H-5″), 6.31–6.26 (each 1H, d, J = 15.9 Hz, H-8′ and H-8″); ^13^C NMR (100.6 MHz, CDCl_3_): Quinic acid; 34.6 (C-2), 36.9 (C-6), 61.1 (C-4), 70.7 (C-3), 71.2 (C-5), 73.3 (C-1), 175.9 (C-7); Feruloyl’; 55.49 (OCH3), 113.8 (C-8′), 114.2 (C-2′), 115.1 (C-5′), 126.8 (C-1′), 145.4 (C-3′), 145.9 (C-7′), 148.1 (C-4′), 167.5 (C-9′); Feruloyl′; 55.17 (OCH3), 113.8 (C-8″), 113.9 (C-2″), 115.1 (C-5″), 126.4 (C-1″), 145.4 (C-3″), 145.7 (C-7″), 148.1 (C-4″), 167.5 (C-9″). Supplementary data (Figures SG.54–SG.60).

#### 3.4.8. Compound H: Isofraxidin-7-*O*-*β*-*D*-glucopyranoside

Pale brown powder (61.512), Rf: 0.25 (CDCl_3_: methanol, 88:12), Melting point: 204–206 °C, [α]_D_^20^ =+59.9°, c=0.48 in DMSO, HR-EI-MS [M-C_6_H_12_O_6_]: found *m/z* 223.0603 for C_17_H_20_O_10_ (calcd 384.3347), IR (KBr) cm^−1^: 3423–3528 (OH^−^), 2979–2870 (medium, C-H), 1715 (strong, C=O), 1576 (medium, C=C aromatic), 1410-1298 (medium, C-O), 1035 secondary alcohols and 839 benzene ring. ^1^H NMR (400 MHz, DMSO-d6) δ 3.81 (3H, s, 6-OCH3), 3.90 (3H, s, 8-OCH3), 5.14 (1H, d, J = 7.3 Hz, H-1′), 6.39 (1H, d, J = 9.5 Hz, H-3), 7.11 (1H, s, H-5), 7.93 (1H, d, J = 9.5 Hz, H-4); glucose; 3.09 (2H, m, H-3″, H-4″), 3.24 (2H, m, H-2″, H-5″), 3.38 (1H, m, H-6″), 3.59 (1H, m, H-6″); ^13^C NMR (100.6 MHz, DMSO-d6) δ 56.5 (6-OCH3), 61.7 (8-OCH3), 105.9 (C-5), 115.0 (C-4′), 115.3 (C-3), 140.7 (C-8), 142.2 (C-7), 142.8 (C-8′), 144.8 (C-4), 149.9 (C-6), 160.3 (C-2); glucose; 61.2 (C-6″), 70.3 (C-4″), 74.5 (C-2″), 76.9 (C-5″), 77.9 (C-3″), 102.6 (C-1″).

### 3.5. Minimum Inhibitory Assay for Quantitative Antimicrobial Test

The MIC value of the plant extracts and isolated compounds against one fungal and five bacterial strains (*Salmonella* Typhimurium ATCC 39183, *Escherichia coli* ATCC 25922, *Pseudomonas aeruginosa* ATCC 27853, *Staphylococcus aureus* ATCC 29213, *Enterococcus faecalis* ATCC 29212, and fungi culture, *Candida albicans* ATCC 10231) was assessed in triplicate using the serial dilution microplate method [31]; acetone and DMSO were used as solvents. Bacterial cultures were grown in Mueller Hinton broth (Sigma Aldrich, St. Louis, MO, USA) overnight and adjusted to McFarland standard 1 (1 × 10^8^ CFU/mL). Then, 100 µL of samples were transferred into the first well of a sterile 96-well microtiter plate, which contained 100 µL of water and a 1:1 serial dilution with sterilized distilled water was carried out. In each well, 100 µL of appropriately adjusted fungal or bacterial cultures were added, and the bacteria were subjected to final concentrations of 250–3.9 μg/mL for pure compounds and 2.5–0.02 mg/mL for crude extracts. Amphotericin-B and Gentamicin (Virbac, Carros, France) served as positive controls for fungi and bacteria, respectively. For sterility, water served as control, while acetone served as a negative control. The microplates were then incubated for 24 h at 37 °C. Following incubation, 40 µL of 0.2 mg/mL p-iodonitrotetrazolium violet (INT, Sigma Aldrich) dissolved in water (hot) was added to the wells before being incubated at 37 °C for another 2 h. In the case of fungi, the INT was added before incubation. The MIC was measured visually as the lowest concentration of the compounds/extracts that inhibited proliferation [31].

### 3.6. Cytotoxicity Assay of Isolated Compounds and Crude Extracts

Cytotoxicity of compounds and extracts was tested against mammalian cells using the MTT assay [69,70]. MCF-7 cancer cells were grown in a 5% CO_2_ incubator in Dulbecco’s Modified Eagle’s Medium (DMEM, Highveld Biological, Johannesburg, South Africa) supplemented with 10% fetal calf serum (Adcock-Ingram, Johannesburg, South Africa) and 1% penicillin-streptomycin (10,000 U/mL and 10 mg/mL streptomycin, Sigma Aldrich). In a 5% CO_2_ incubator, Vero cells were grown in a minimal essential medium (MEM, Highveld Biological, South Africa) supplemented with 5% fetal calf serum (Adcock-Ingram) and 0.1% gentamicin (Virbac). Cell suspensions were obtained from 70–80% confluent monolayer cultures and were plated in 96-well cell culture plates at a density of 1 × 10^4^. Plates were incubated in a 5% CO_2_ incubator for 24 h at 37 °C. The cells were exposed to the extracts or compounds dissolved in the media at different concentrations for 48 h. Positive and negative controls were doxorubicin (Pfizer, New York, NYC, USA) and acetone or DMSO, respectively. After that, the wells were rinsed with phosphate-buffered saline (PBS, Sigma) and 200 µL of fresh medium was poured into the wells. Thirty microlitres (5 mg/mL) of MTT (Sigma) dissolved in PBS was added to each well, and the plates were incubated at 37 °C for 4 h. The medium from the wells was removed, rinsed, and 50 µL of 100% DMSO was added to the wells. At a wavelength of 570 nm, absorbance was measured using a microplate reader (BioTek Synergy, Beijing, China). Each sample concentration was tested in quadruplicate in two repeats (n = 8), and the concentration causing 50% inhibition of cell viability (LC_50_) was calculated. Selectivity index (SI) values for the extracts/compounds were obtained by dividing cytotoxicity LC_50_ values of normal (Vero) cells by those of the cancer cells.

## 4. Conclusions

Eight compounds (**A**–**H**) were isolated from *A. afra* extracts through chromatographic techniques. By comparing spectroscopic data obtained with the one from the literature, the compounds were correctly identified as 3*β*-taraxerol (**A**), 3*β*-taraxerol acetate (**B**), dodecyl-*p*-coumarate (**C**), ferulic acid (**D**), scopoletin (**E**), sitosterol-3-*O*-*β*-*D*-glucopyranoside (**F**), 3,5-di-*O*-feruloylquinic acid (**G**) and isofraxidin-7-*O*-*β*-D-glucopyranoside (**H**). 3*β*-Taraxerol and 3*β*-taraxerol acetate are identified for the first time according to our knowledge from *Artemisia afra roots* and wormwood species, even though the compounds were previously isolated from other plant species. Moreover, dodecyl-*p*-coumarate, sitosterol-3-*O*-*β*-*D*-glucopyranoside, 3,5-di-*O*-feruloylquinic acid, and isofraxidin-7-*O*-*β*-*D*-glucopyranoside were also identified for the first time from the roots of *Artemisia afra*.

The hexane, DCM/EtOAc and EtOH extracts of *A. afra* exhibited good antimicrobial activity depending on concentration against both Gram-positive and Gram-negative bacteria and fungi, with DCM and EtOAc crude extracts showing exceptional activity against *Escherichia coli* (MIC = 0.078 mg/mL). Scopoletin (**E**) isolated from the DCM and EtOAc crude showed very good activity against *Escherichia coli* (MIC = 62.5 µg/mL) and 3β-sitosterol glucopyranoside (**F**) isolated from the ethanol extract and dodecyl-*p*-coumarate isolated from the DCM/EtOAc crude extract showed very good activity against *Enterococcus faecalis* (MIC = 31.25 and 62.5 µg/mL, respectively). The remaining compounds (ferulic acid, 3,5-di-*O*-feruloylquinic acid, and isofraxidin-7-*O*-*β*-*D*-glucopyranoside) displayed no efficacy against the strains. However, earlier research reports that these compounds (**A**–**H**) have strong antioxidant and anti-inflammatory properties [1,28,37]. The isolated compounds were active at their highest concentration (MIC = 250 µg/mL), and all the crude extracts (Hex, DCM/EtOAc, and EtOH) and isolated compounds (**A**–**H**) were determined to be non-toxic to Vero cells and displayed modest efficacy against *C. albicans*.

## Figures and Tables

**Table 1:** Minimum inhibitory concentration (MIC) and cytotoxicity studies (against Vero cells) of crude extracts. The selectivity index is given in the brackets. Values in bold show a good selectivity index value. Values are given in mg/mL.

Crude Extracts	*E. coli*	*E. faecalis*	*P. aeruginosa*	*S. aureus*	*S.* Typhimurium	*C. albicans*	LC_50_
HEX	>2.50 (<0.152)	0.156 (**2.42**)	1.25 (0.304)	>2.50 (<0.152)	>2.50 (<0.152)	0.625 (0.61)	0.38
EA & DCM	0.078 (0.641)	0.156 (0.321)	>2.5 (<0.02)	1.25 (0.04)	0.625 (0.08)	0.625 (0.08)	0.05
ETOH	0.625 (0.192)	0.156 (0.77)	2.5 (0.048)	0.625 (0.192)	0.625 (0.192)	1.25 (0.096)	0.12
Gentamicin	0.02	0.02	0.02	0.02	0.02	-	-
Amphotericin-B	-	-	-	-	-	0.02	-
Doxorubicin	-	-	-	-	-	-	0.01

Gentamicin: Positive control, Amphotericin-B: Positive control, Doxorubicin (Pfizer): Positive control, (-): Not determined.

**Table 2:** Minimum inhibitory concentration (MIC) and cytotoxicity studies of isolated compounds. Values are given in µg/mL. The selectivity index is given in the brackets. Values in bold show a good selectivity index value. Gentamicin: Positive control; Amphotericin-B: Positive control; (-): Not determined.

Isolated Compounds	*E. coli*	*E. faecalis*	*P. aeruginosa*	*S. aureus*	*S.* Typhimurium	*C. albicans*	LC_50_
3*β*-Taraxerol	250 (>0.8)	>250 (<0.8)	>250 (<0.8)	250 (>0.8)	250 (>0.8)	250 (>0.8)	>200
Dodecyl-*p*-coumarate	>250 (<0.8)	62.5 (**>3.2**)	>250 (<0.8)	>250 (<0.8)	>250 (<0.8)	250 (>0.8)	>200
Ferulic acid	>250 (<0.8)	>250 (<0.8)	>250 (<0.8)	250 (>0.8)	>250 (<0.8)	250 (>0.8)	>200
Scopoletin	62.5 (**>3.2**)	250 (0.8)	>250 (<0.8)	250 (>0.8)	125 (**>1.6**)	250 (>0.8)	>200
3,5-Di-*O*-feruloylquinic acid	>250 (>0.8)	>250 (>0.8)	>250 (>0.8)	>250 (>0.8)	>250 (>0.8)	>250 (>0.8)	>200
Sitosterol-3-*O*-*β*-*D*-glucopyranoside	>250 (<0.8)	31.25 (**>6.4**)	>250 (<0.8)	>250 (<0.8)	>250 (<0.8)	250 (>0.8)	>200
Isofraxidin-7-*O*-*β*-*D*-glucopyranoside	250 (>0.8)	>250 (<0.8)	>250 (<0.8)	250 (0.8)	250 (>0.8)	250 (>0.8)	>200
Gentamicin	3.9	3.9	3.9	3.9	3.9	-	-
Amphotericin-B	-	-	-	-	-	3.9	-
Doxorubicin	-	-	-	-	-	-	0.01

Gentamicin: Positive control; Amphotericin-B: Positive control; Doxorubicin (Pfizer): Positive control; (-): Not determined.

## Data Availability

Not applicable.

## Supplementary Materials

The following supporting information can be downloaded at: https://www.mdpi.com/article/10.3390/plants12193369/s1, Table S1: ^1^H (δ_H_; J, Hz) NMR (400 MHZ) data for compounds **A**–**H** in CDCl_3,_ MeOD and DMSO; Table S2: ^13^C (δc) (100.6 MHz) NMR data for compounds A-H in CDCl_3_, MeOD and DMSO and S2, Figures SA.1–SH.68: Spectral data for compounds (**A**–**H**).
