# Supplementary material for: Extractives from Artemisia afra with Anti-Bacterial and Anti-Fungal Properties
Source: Plants (Basel). 2023 Sep 25;12(19):3369. doi: 10.3390/plants12193369 (PMC10574767; doi:10.3390/plants12193369)
Supplement: Supplementary file 1 [file plants-12-03369-s001.zip › plants-2623315-supplementary.pdf]

Article

Extractives from *Artemisia afra* with anti-bacterial and anti-fungal properties

Tumelo L. Molokoane [1], Douglas Kemboi [1,2,*], Xavier Siwe-Noundou [3], Ibukun M. Famuyide [4], Lyndy J. McGaw [4] and Vuyelwa J. Tembu [1,*]

[1] Department of Chemistry, Tshwane University of Technology, Private Bag X680, Pretoria 0001, South Africa; tumelolinahmolokoane@gmail.com

[2] Department of Chemistry, University of Kabianga, Kericho 2030, Kenya

[3] Department of Pharmaceutical Sciences, Sefako Makgatho Health Sciences University, Pretoria 0204, South Africa; xavier.siwenoundou@smu.ac.za

[4] Phytomedicine Programme, Department of Paraclinical Sciences, University of Pretoria, Private Bag X04, Onderstepoort 0110, South Africa; adeyerimi@gmail.com (I.M.F.); lyndy.mcgaw@up.ac.za (L.J.M.)

* Correspondence: kemboidouglas01@gmail.com (D.K.); tembuvj@tut.ac.za (V.J.T.)

Supplementary data

Table 1: ^{1}H (δ_H; J, Hz) NMR (400 MHZ) data for compounds A-H in $CDCl_3$, MeOD and DMSO

	Compound A	Compound B	Compound C	Compound D	Compound E	Compound F	Compound G	Compound H
1	0.92-1.63 (2H, m)	1.92-1.97 (2H, m)	-	-	-	0.94-1.76 (2H, m)	-	-
2	1.61 (2H, m)	1.64 (2H, m)	6.34 (1H, d, J = 15.9 Hz)	6.34 (1H, d, *J* = 15.9 Hz)	-	1.21-1.62 (2H, m)	2.16-2.36 (2H, m)	-
3	3.20 (1H, dd, *J*=11.1, 4.8 Hz)	4.47 (1H, dd, *J*=10.2, 5.6 Hz)	7.63 (1H, d, *J* = 15.9 Hz)	7.60 (1H, d, *J* = 15.9 Hz)	6.28 (1H, d, *J* = 9.5 Hz)	3.09 (1H, m)	5.41 (1H, m)	6.39 (1H, d, J 9.5 Hz)
4	-	-	-	-	7.60 (1H, d, *J* = 9.5 Hz)	2.11-2.33 (2H, m)	4.00 (1H, dd, *J* =3.0, 7.1 Hz)	7.93 (1H, d, J 9.5 Hz)
5	0.78 (1H, m)	0.92 (1H, m)	7.43 (2H, d, *J* = 8.4 Hz)	6.88 (1H, d, *J* = 8.2 Hz)	6.87 (1H, s)	-	5.46 (1H, ddd, *J*= 3.0, 7.1, 7.1 Hz)	7.11 (1H, s)
6	1.52 -1.67 (2H, m)	1.61-1.66 (2H, m)	6.87 (2H, d, *J* = 8.4 Hz)	-	-	5.31 (1H, d, *J* = 4.8 Hz)	2.03-2.25 (2H, m)	-
7	1.36 - 2.03 (2H, m)	1.25-1.36 (2H, m)	-	-	-	1.40-1.88 (2H, m)	-	-
8	-	-	6.87	7.16 (1H, s)	6.94 (1H, s)	1.37 (1H m)	-	-

9	1.44 (1H, m)	1.47 (1H, m)	7.43	7.04 (1H, s)	-	0.88 (1H, m)	-	-
10	-	-	-	-	-		-	-
11	1.45-1.70 (2H, m)	1.47-1.67 (2H, m)	-	-	-	1.48 (2H m)	-	-
12	1.56-1.64 (2H, m)	1.30-1.34 (2H, m)	-	-	-	1.92-2.49 (2H, m)	-	-
13	-	-	-	-	-	-	-	-
14	-	-	-	-	-	1.06 (1H, m)	-	-
15	5.55 (1H, dd, *J*=8.2, 3.2 Hz)	5.55 (1H, dd, *J*=8.0, 3.8 Hz)	-	-	-	0.99-1.51 (2H, m)	-	-
16	1.66-1.97 (1H, m: 1H, dd, *J*=14.7, 3.2 Hz)	1.64-1.67 (2H, m)	-	-	-	1.23-1.79 (2H, m)	-	-
17	-	-	-	-	-	1.08 (1H, m)	-	-
18	0.95 (1H, m)	0.93 (1H, m)	-	-	-	0.63 (3H, s)	-	-
19	0.97-1.30 (2H, m)	1.38-1.42 (2H, m)	-	-	-	0.94 (3H, s)	-	-
20	-	-	-	-	-	1.21 (1H, m)	-	-

21	1.25-1.31 (2H, m)	1.02-1.42 (2H, m)	-	-	-	0.88 (3H, s)	-	-
22	1.02-1.34 (2H, m)	1.06-1.10 (2H, m)	-	-	-	1.23-1.45 (2H, m)	-	-
23	1.00 (3H, s)	0.88 (3H, s)	-	-	-	1.12-1.14 (2H, m)	-	-
24	0.83 (3H, s)	0.97 (3H, s)	-	-	-	0.89 (1H, m)	-	-
25	0.95 (3H, s)	0.97 (3H, s)	-	-	-	1.60 (1H, m)	-	-
26	1.11 (3H, s)	1.11 (3H, s)	-	-	-	0.79 (3H, s)	-	-
27	0.94 (3H, s)	0.92 (3H, s)	-	-	-	0.97 (3H, s)	-	-
28	0.85 (3H, s)	0.85 (3H, s)	-	-	-	1.19 (2H, m)	-	-
29	0.97 (3H, s)	0.97 (3H, s)	-	-	-	0.80 (3H, s)	-	-
30	0.92 (3H, s)	0.92 (3H, s)	-	-	-	-	-	-
31	-	2.06 (3H, s)	-	-	-	-	-	-
1′	-	-	4.22 (2H, t, *J* = 6.7 Hz)	-	-	4.20 (1H, m)	-	5.14 (1H, d, J 7.3 Hz)
2′	-	-	1.72 (2H, m)	-	-	2.88 (1H, m)	7.09 (1H, s)	3.24 (1H, m)

3′	-	-	1.41 (2H, dd, *J* = 9.7, 5.8 Hz)	-	-	3.09 (1H, m)		3.10 (1H, m)
4′	-	-	1.28 (1H, s)	-	-	3.02 (1H, m)		3.09 (1H, m)
5′	-	-	1.28 (1H, s)	-	-	3.04 (1H, m)	6.81 (1H, d, *J* = 8.2 Hz)	3.23 (1H, m)
6′	-	-	1.28 (1H, s)	-	-	3.64 (1H, m)	6.97 (1H, d, *J* = 8.2 Hz)	3.38 (1H, m) 3.59 (d, J = 11.7 Hz, 1H)
7′	-	-	1.28 (1H, s)	-	-	-	7.65 (1H, d, *J* = 15.9 Hz)	-
8′	-	-	1.28 (1H, s)	-	-	-	6.31 (1H, d, *J* = 15.9 Hz)	-
9′	-	-	1.28 (1H, s)	-	-	-	-	-
10′	-	-	1.28 (1H, s)	-	-	-	-	-
11′	-	-	1.28 (1H, s)	-	-	-	-	-
12′	-	-	0.91 (3H, t, J = 6.5 Hz)	-	-	-	-	-
1′′	-	-	-	-	-	-	-	-
2′′	-	-	-	-	-	-	7.08 (1H, s)	-

3′′	-	-	-	-	-	-	-	-
4′′	-	-	-	-	-	-	-	-
5′′	-	-	-	-	-	-	6.81 (1H, d, *J* = 8.2 Hz)	-
6′′	-	-	-	-	-	-	6.96 (1H, d, *J* = 8.2 Hz)	-
7′′	-	-	-	-	-	-	7.61 (1H, d, *J* = 15.9 Hz)	-
8′′	-	-	-	-	-	-	6.26 (1H, d, *J* = 15.9 Hz)	-
9′′	-	-	-	-	-	-	-	-
OCH_3	-	-	-	3.95 (3H, s)	3.98 (3H, s)	-	3.87 (3H, s)	3.81 (3H, s)
OCH_3	-	-	-	-	-	-	3.90 (3H, s)	3.90 (3H, s)
OH	-	-	-	-	6.19 (1H, s)	-	-	-

Key: *s* – singlet, *d* – doublet, *dd* – doublet of doublet, *t* – triplet, *m* – multiplet, Hz – hertz, (-) - not determined

Table 2: ^{13}C (δc) (100.6 MHz) NMR data for compounds A-H in $CDCl_3$, MeOD and DMSO

	Compound A	Compound B	Compound C	Compound D	Compound E	Compound F	Compound G	Compound H

1	37.7 CH_2	37.7 CH_2	168.0	167.7 C		37.2 CH_2	73.3 C	
2	27.2 CH_2	23.5 CH_2	115.5 CH	115.9 CH	161.5 C	29.7 CH_2	34.6 CH	160.3 C
3	79.1 CH	81.00 CH	144.6 CH	144.7 CH	113.4 CH	77.4 CH	70.7 CH	115.1 CH
4	38.8 C	37.9 C	127.0	128.1 C	143.4 CH	38.3 CH_2	61.1 CH	144.8 CH
5	55.5 CH	55.6 CH	130.0 CH	110.5 CH	107.4 CH	140.9 C	71.2 CH	105.9 CH
6	18.8 CH_2	18.7 CH_2	115.9 CH	148.5 C	144.0 C	121.7 CH	36.9 CH	149.9 C
7	41.3 CH_2	33.1 CH_2	158.0	145.9 C	149.7 C	31.9 CH_2	175.9	142.2 C
8	39.0 C	39.0 C	115.9 CH	113.0 CH	103.2 CH	31.8 CH	-	140.7 C
9	49.3 CH	49.2 CH	130.0 CH	121.8 CH	-	50.1 CH	-	-
10	38.0 C	37.4 C	-	-	-	36.7 C	-	-
11	17.5 CH_2	17.5 CH_2	-	-	-	21.04 CH_2	-	-
12	33.7 CH_2	36.7 CH_2	-	-	-	40.2 CH_2	-	-
13	37.6 C	37.6 CH	-	-	-	42.3 C	-	-
14	158.1 C	158.0 C	-	-	-	56.6 CH	-	-
15	116.9 CH	117.0 CH	-	-	-	24.3 CH_2	-	-
16	37.7 CH_2	33.7 CH_2	-	-	-	28.2 CH_2	-	-
17	35.8 C	35.8 C	-	-	-	55.9 CH	-	-
18	48.8 CH	48.8 CH	-	-	-	12.2 CH_3	-	-
19	36.7 CH_2	41.2 CH_2	-	-	-	19.4 CH_3	-	-
20	28.8 C	28.8 C	-	-	-	35.9 CH	-	-
21	33.1 CH_2	35.1 CH_2	-	-	-	19.1 CH_3	-	-

22	35.1 CH_2	37.4 CH_2	-	-	-	33.80 CH_2	-	-
23	28.0 CH_3	28.0 CH_3	-	-	-	25.9 CH_2	-	-
24	15.5 CH_3	16.6 CH_3	-	-	-	45.6 CH	-	-
25	15.4 CH_3	15.5 CH_3	-	-	-	29.2 CH	-	-
26	25.9 CH_3	25.9 CH_3	-	-	-	19.6 CH_3	-	-
27	29.9 CH_3	29.90 CH_3				20.1 CH_3		
28	29.8 CH_3	29.8 CH_3	-	-	-	23.1 CH_2	-	-
29	33.4 CH_3	33.3 CH_3	-	-	-	12.1 CH_3	-	-
30	21.3 CH_3	21.3 CH_3	-	-	-	-	-	-
31	-	170.93 C=O	-	-	-	-	-	-
32	-	21.3 CH_3	-	-	-	-	-	-
1′	-	-	64.8 CH_2	-	-	101.2 CH	126.8 C	-
2′	-	-	28.8 CH_2	-	-	73.9 CH	114.2 CH	-
3′	-	-	26.00 CH_2	-	-	77.2 CH	145.4 C	-
4′	-	-	29.72 CH_2	-	111.5 C	70.6 CH	148.1 C	115.0 C
5′	-	-	29.62 CH_2	-	-	77.12 CH	115.1 CH	-
6′	-	-	29.38 CH_2	-	-	61.6 CH_2	121.7 CH	-
7′	-	-	29.32 CH_2	-	-	-	145.9 CH	-
8′	-	-	29.56 CH_2	-	150.3 C	-	113.8 CH	142.8 C
9′	-	-	29.68 CH_2	-	-	-	167.5 C	-
10′	-	-	32.0 CH_2	-	-	-	-	-

11′	-	-	22.7 CH_2	-	-	-	-	-
12′	-	-	14.1 CH_3	-	-	-	-	-
1′′	-	-	-	-	-	-	126.4 C	102.6 CH
2′′	-	-	-	-	-	-	113.9 CH	74.5 CH
3′′	-	-	-	-	-	-	145.4 C	77.9 CH
4′′	-	-	-	-	-	-	148.1 C	70.3 CH
5′′	-	-	-	-	-	-	115.1 CH	76.9 CH
6′′	-	-	-	-	-	-	121.6 CH	61.2 CH_2
7′′	-	-	-	-	-	-	145.7 CH	-
8′′	-	-	-	-	-	-	113.8 CH	-
9′′	-	-	-	-	-	-	167.0 C	-
OCH_3	-	-	-	56.0	56.4	-	55.49	56.5
OCH_3							55.17	61.7

1. Spectral data of compound A

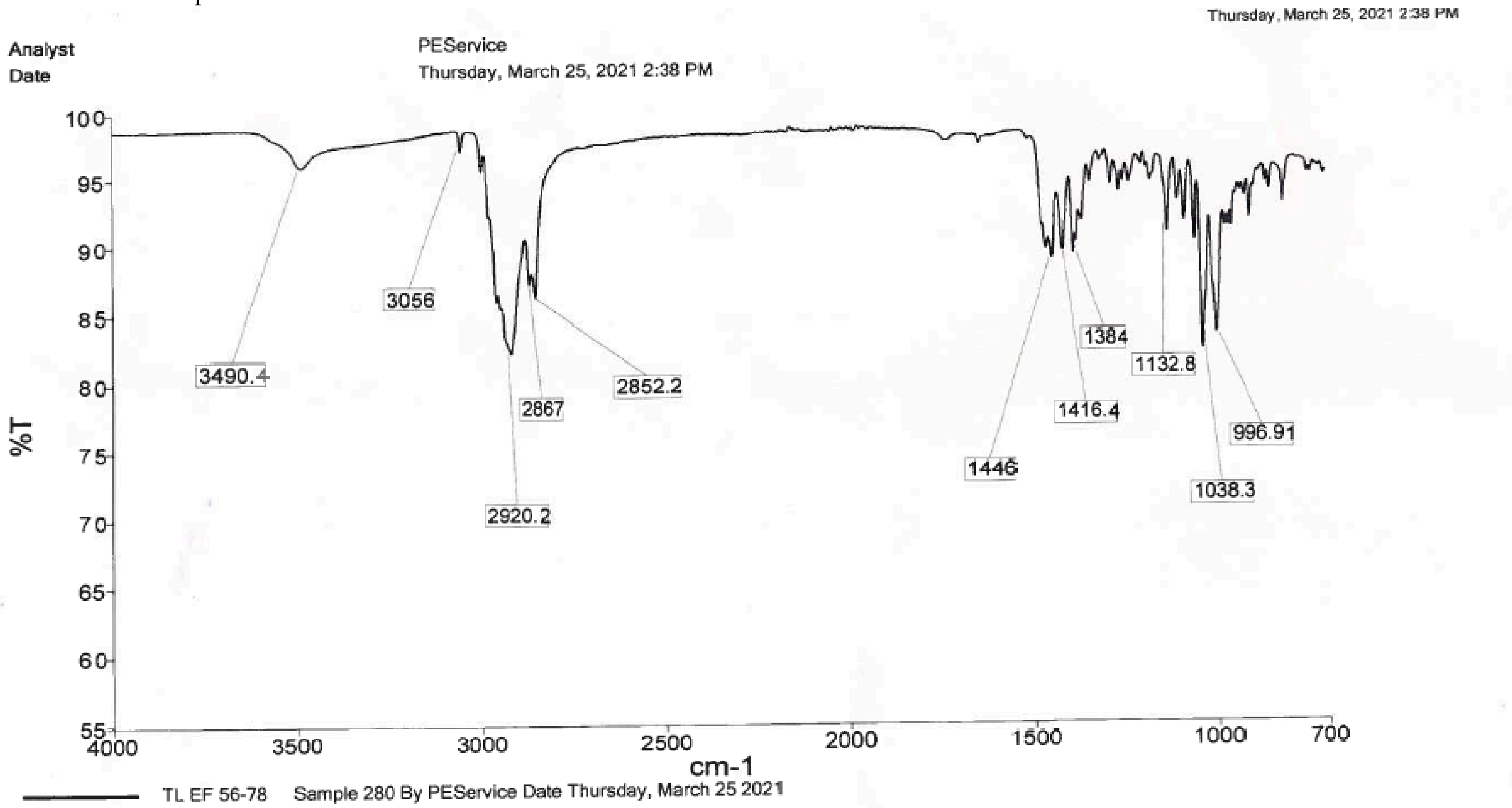

Figure SA.1: Fourier-Transform Infrared Spectroscopy (FTIR) spectrum of (3β)- D-Friedoolean-14-en-3-ol (3β Taraxerol).

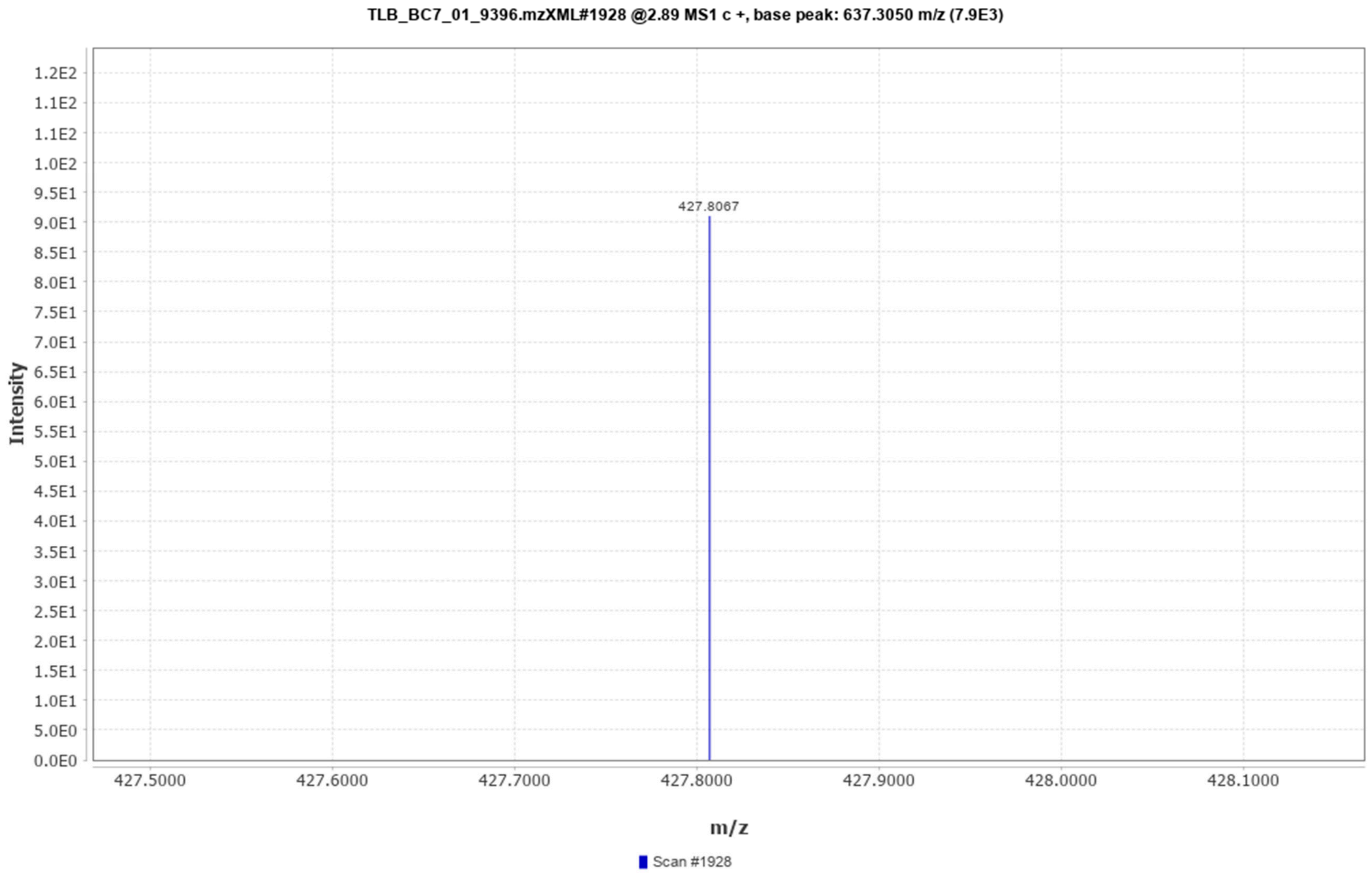

Figure SA.2: High-Resolution Electrospray Ionization Mass spectrum (HR-ESI-MS) of 3β Taraxerol $[M+H]^+$ m/z = 427.8067

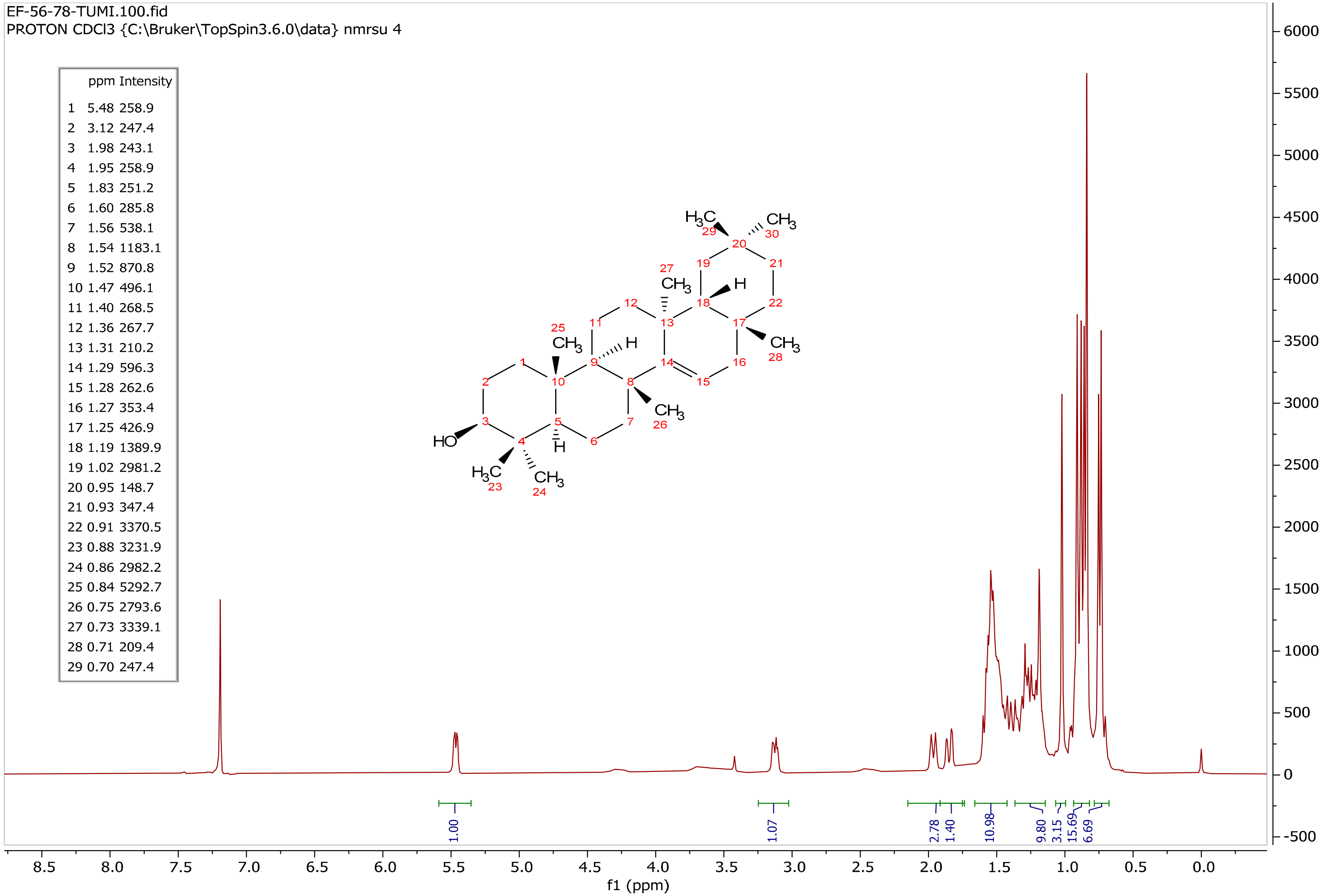

Figure SA.3: Proton Nuclear Magnetic Resonance (^{1}H NMR) spectrum of 3β Taraxerol ($CDCl_3$, 400 MHz)

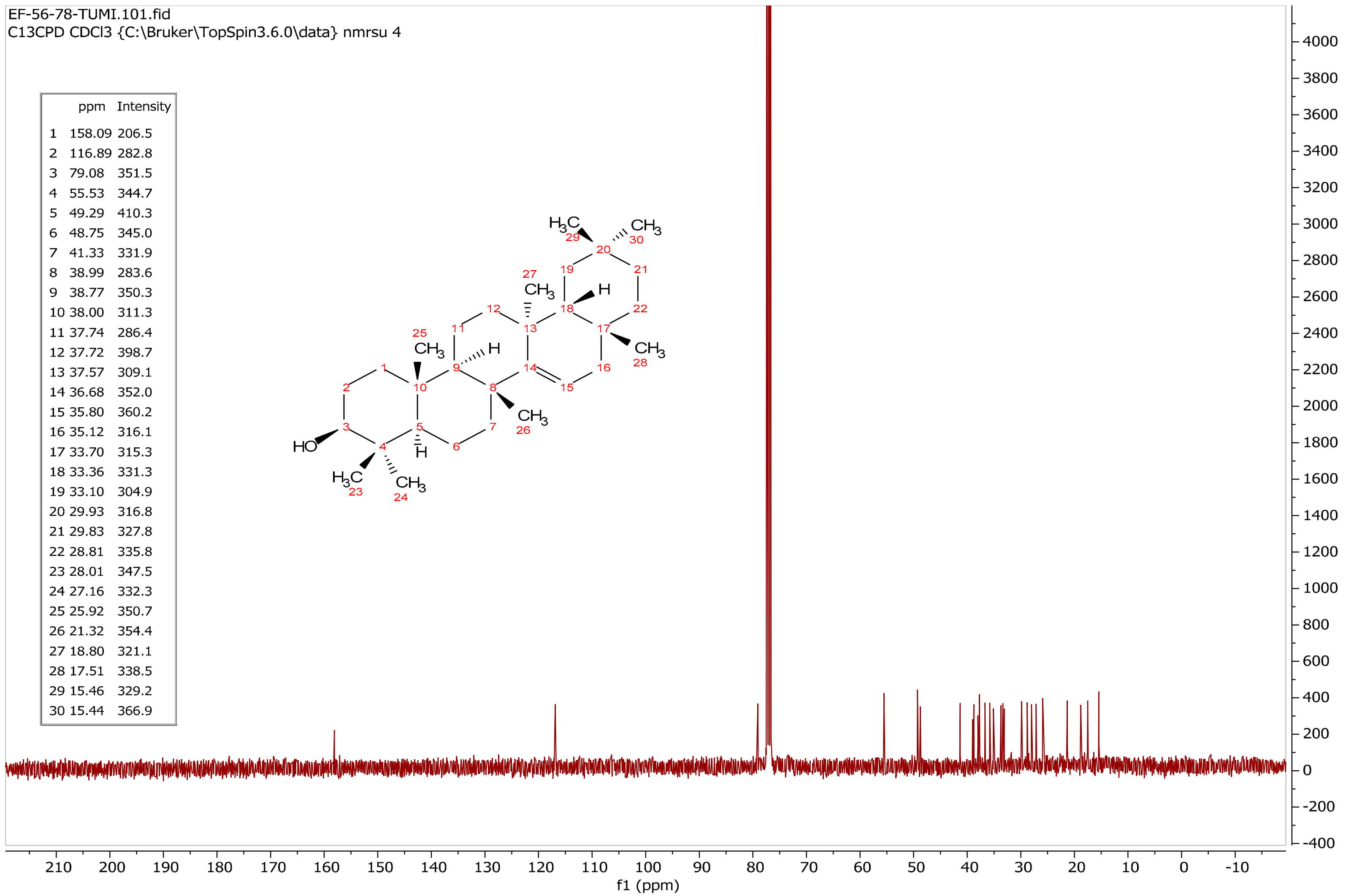

Figure SA.4: Carbon-13 Nuclear Magnetic Resonance (^{13}C NMR) spectrum of 3β Taraxerol ($CDCl_3$, 100 MHz)

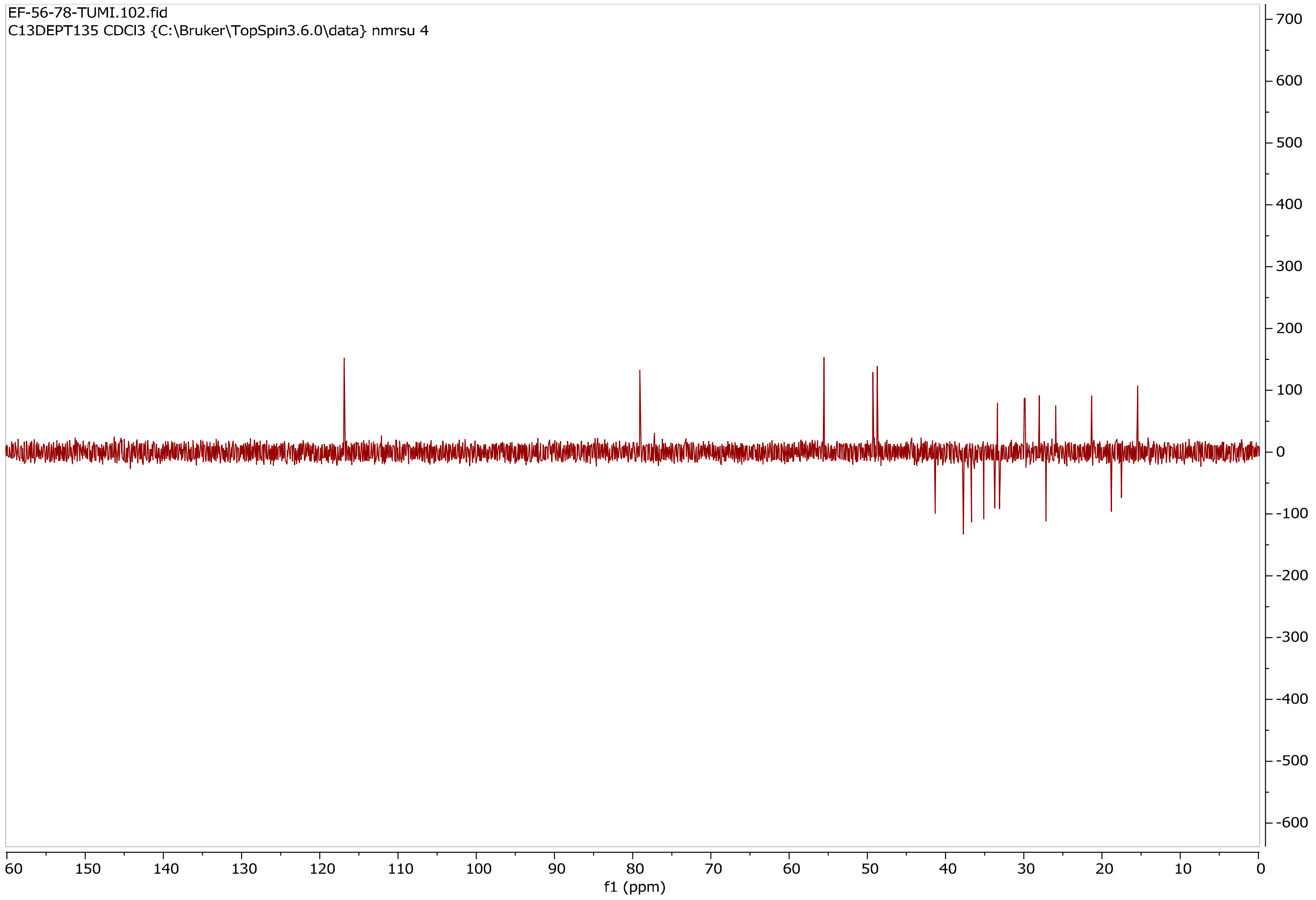

Figure SA.5: Distortionless Enhancement by Polarization Transfer (DEPT) NMR spectra of 3β Taraxerol ($CDCl_3$, 100 MHz)

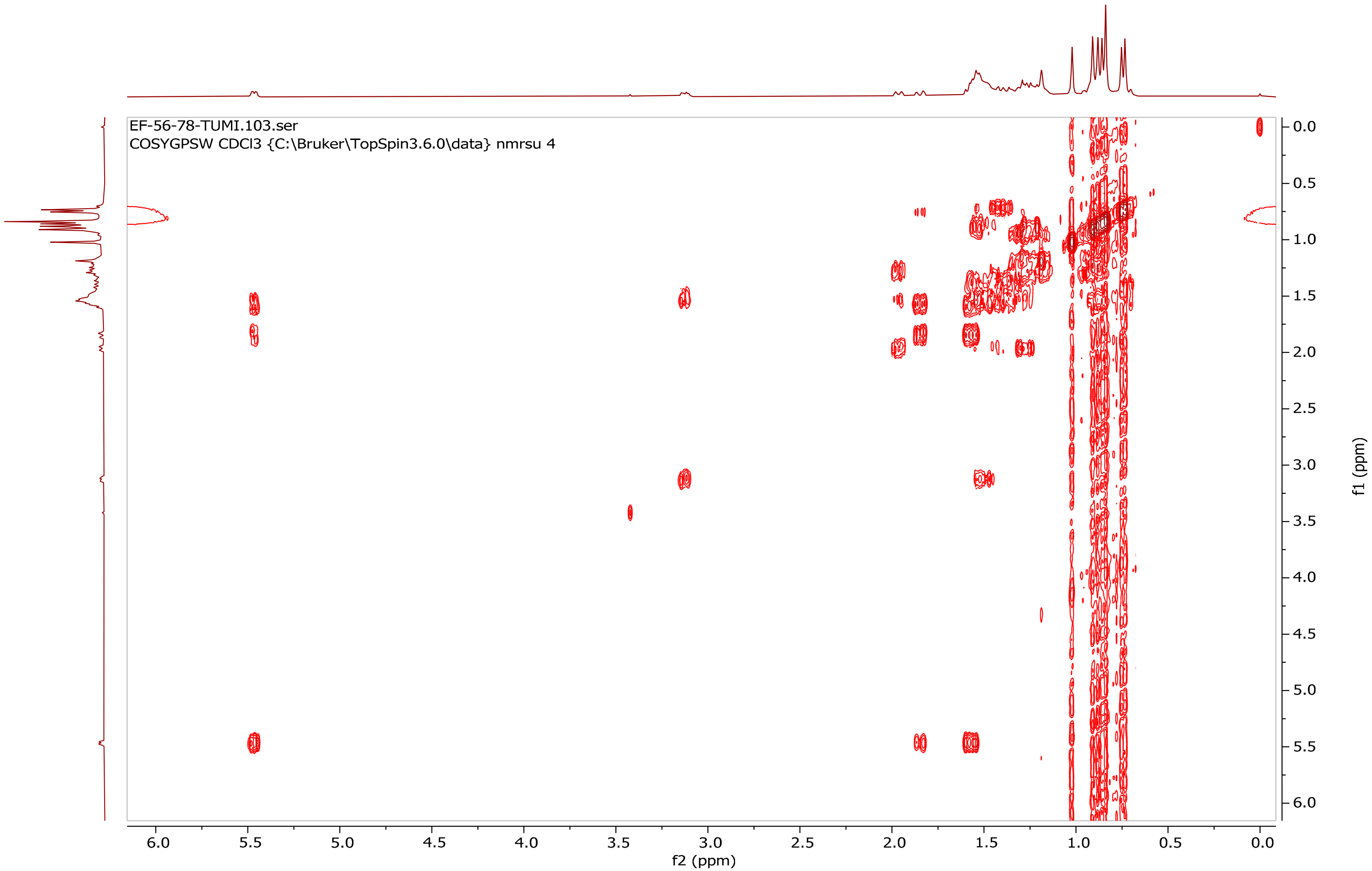

Figure SA.6: Gradient Correlated (gCOSY) spectrum of 3β Taraxerol

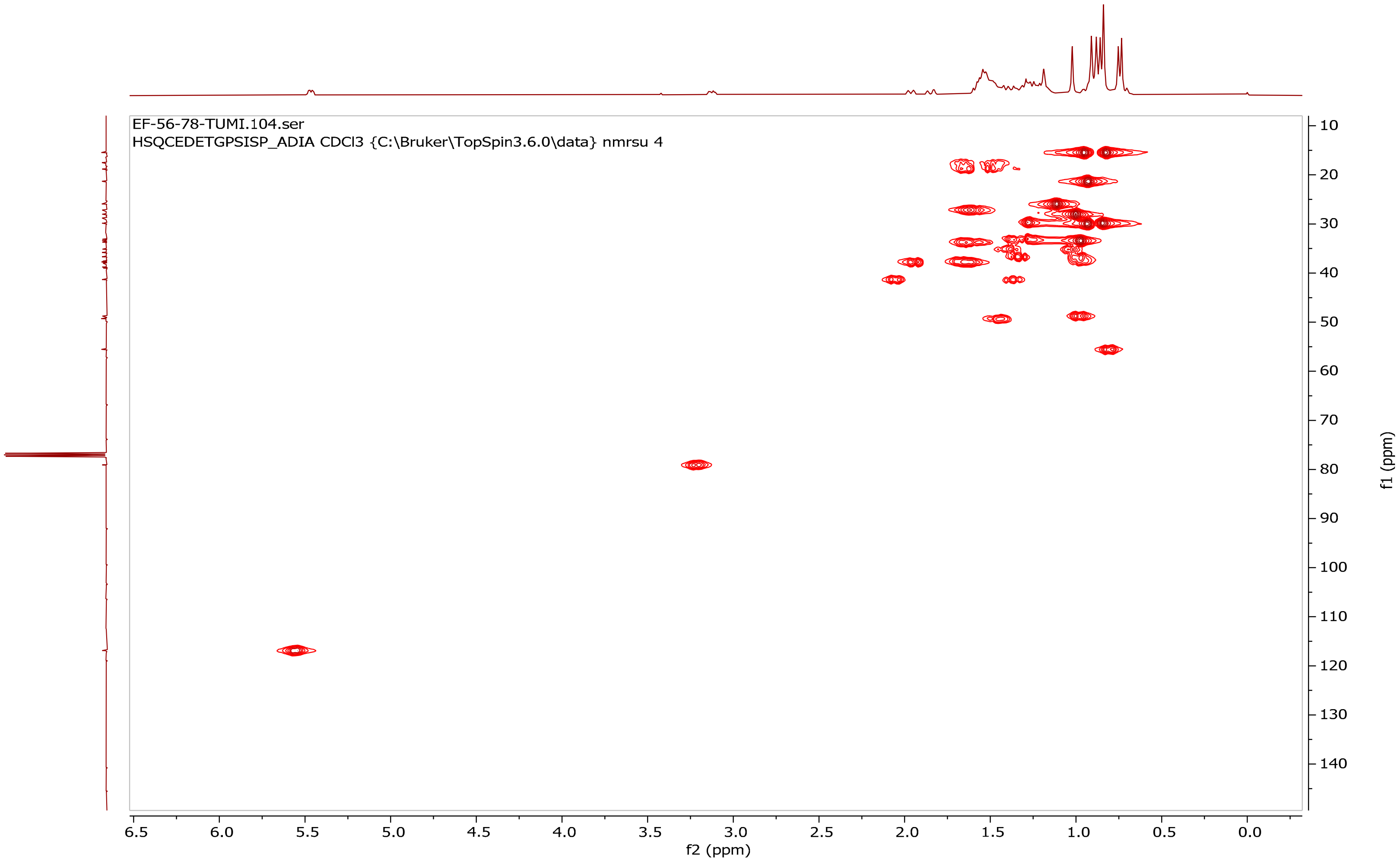

Figure SA.7: Gradient Heteronuclear Single Quantum Coherence (gHSQC) spectrum of 3β Taraxerol

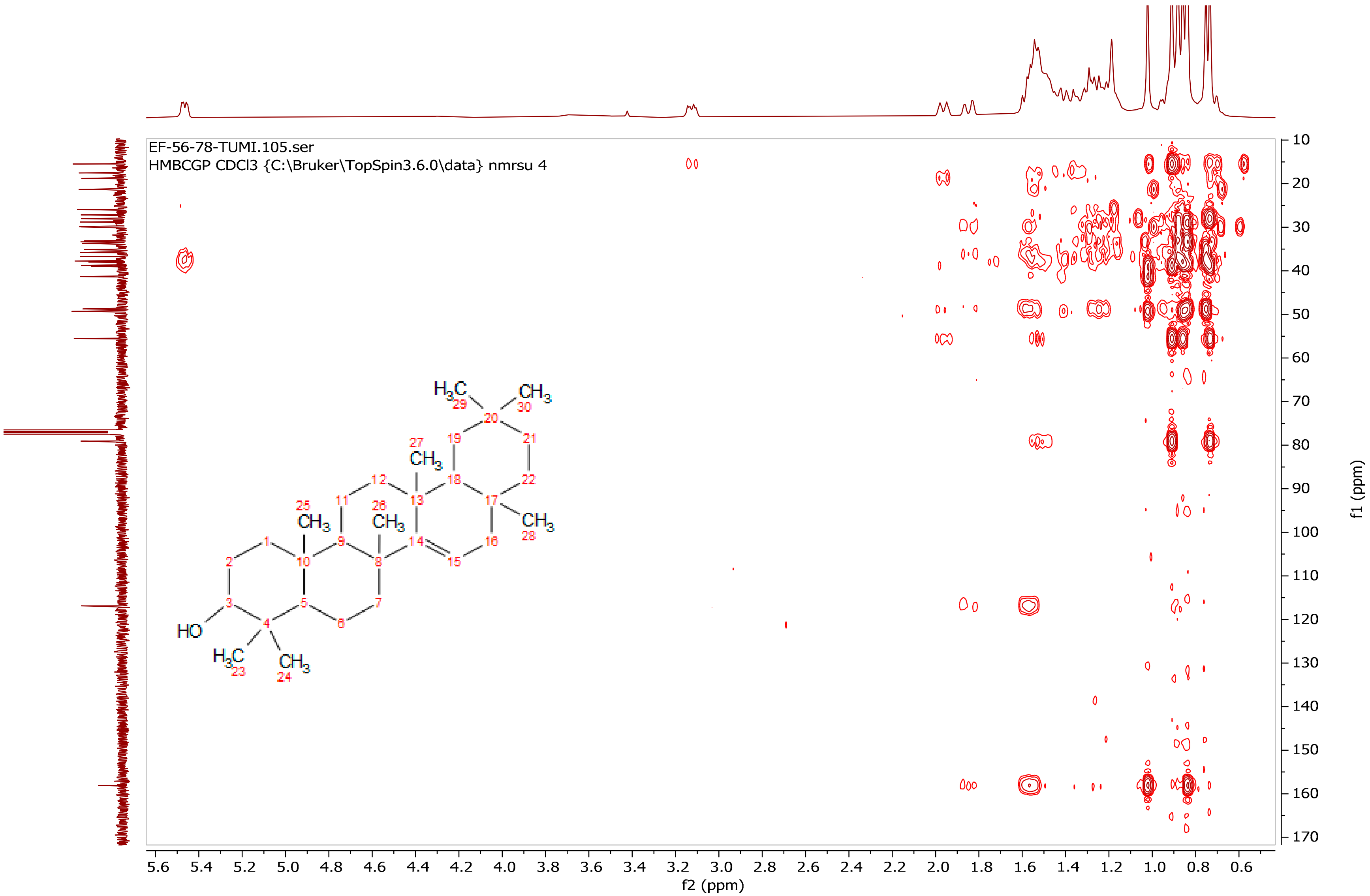

Figure SA.8: Gradient Heteronuclear Multiple Bond Quantum Coherence (gHMBC) spectrum of 3β Taraxerol

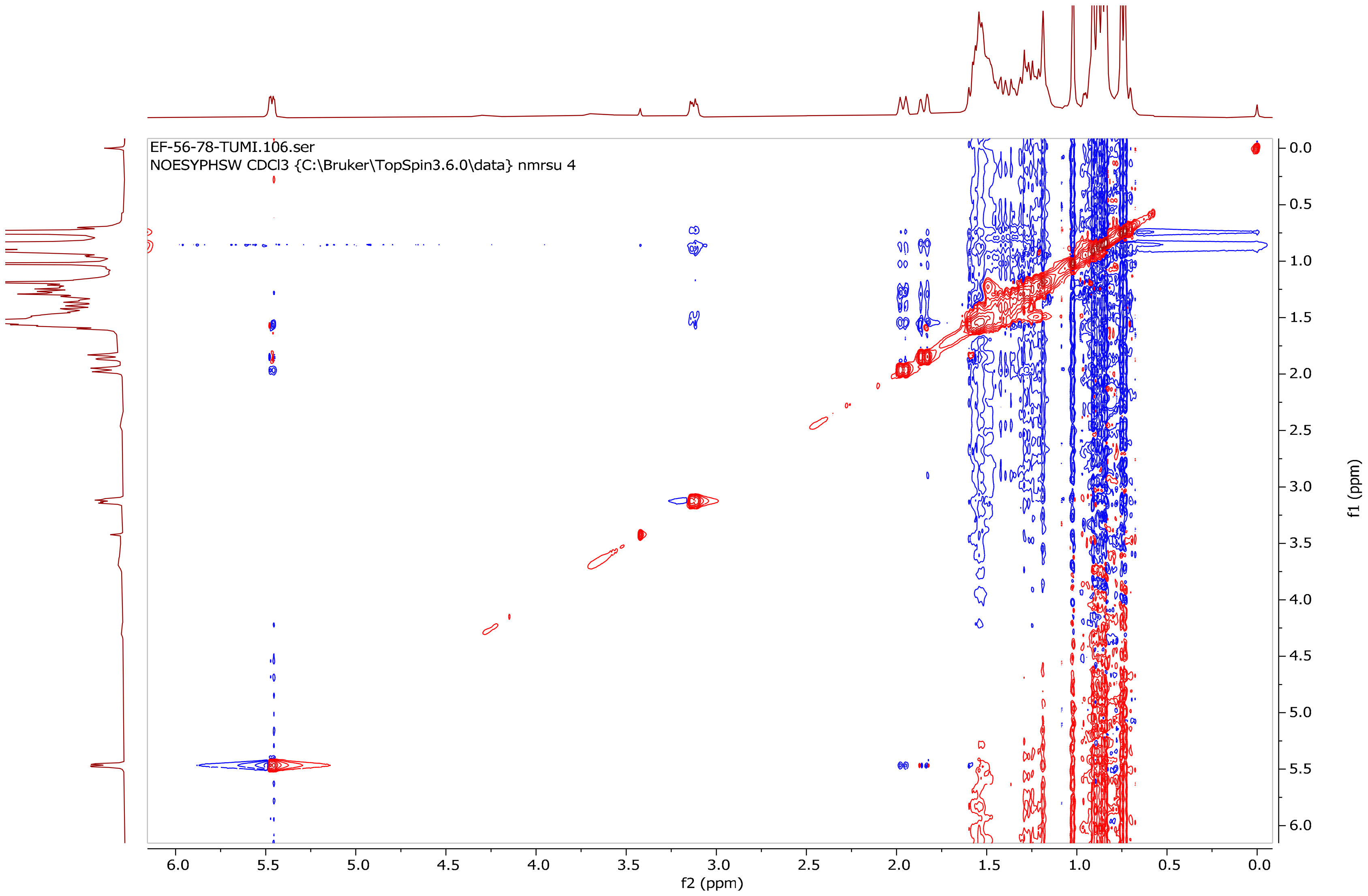

Figure SA.9: Nuclear Overhauser Effect Spectroscopy (NOESY) spectrum of 3β Taraxerol

2. **Spectral data of Compound B**

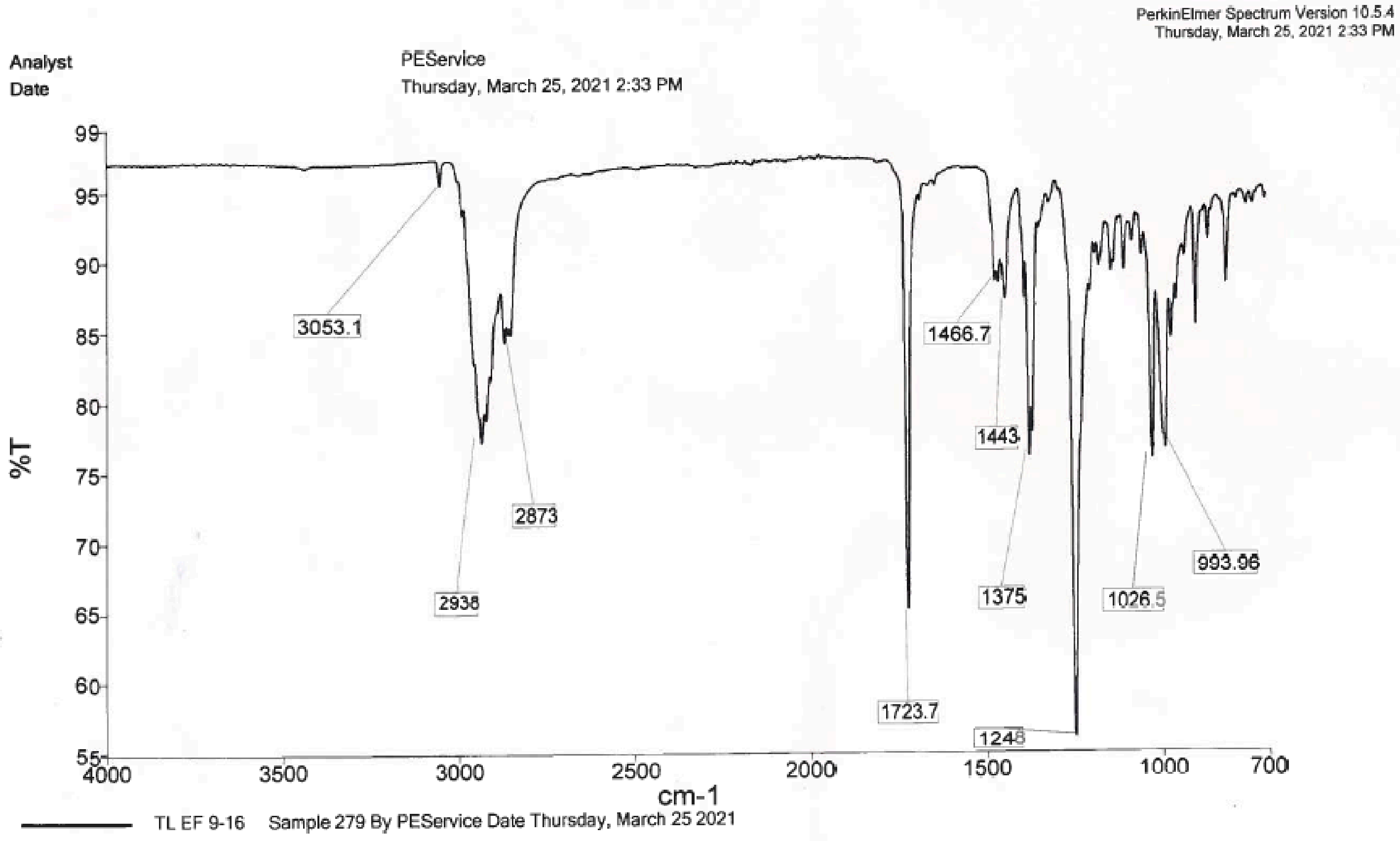

Figure SB.10: Fourier-Transform Infrared Spectroscopy (FTIR) spectrum of Acetyl (3β)- D-Friedoolean-14-en-3-ol (3β Acetyl taraxerol)

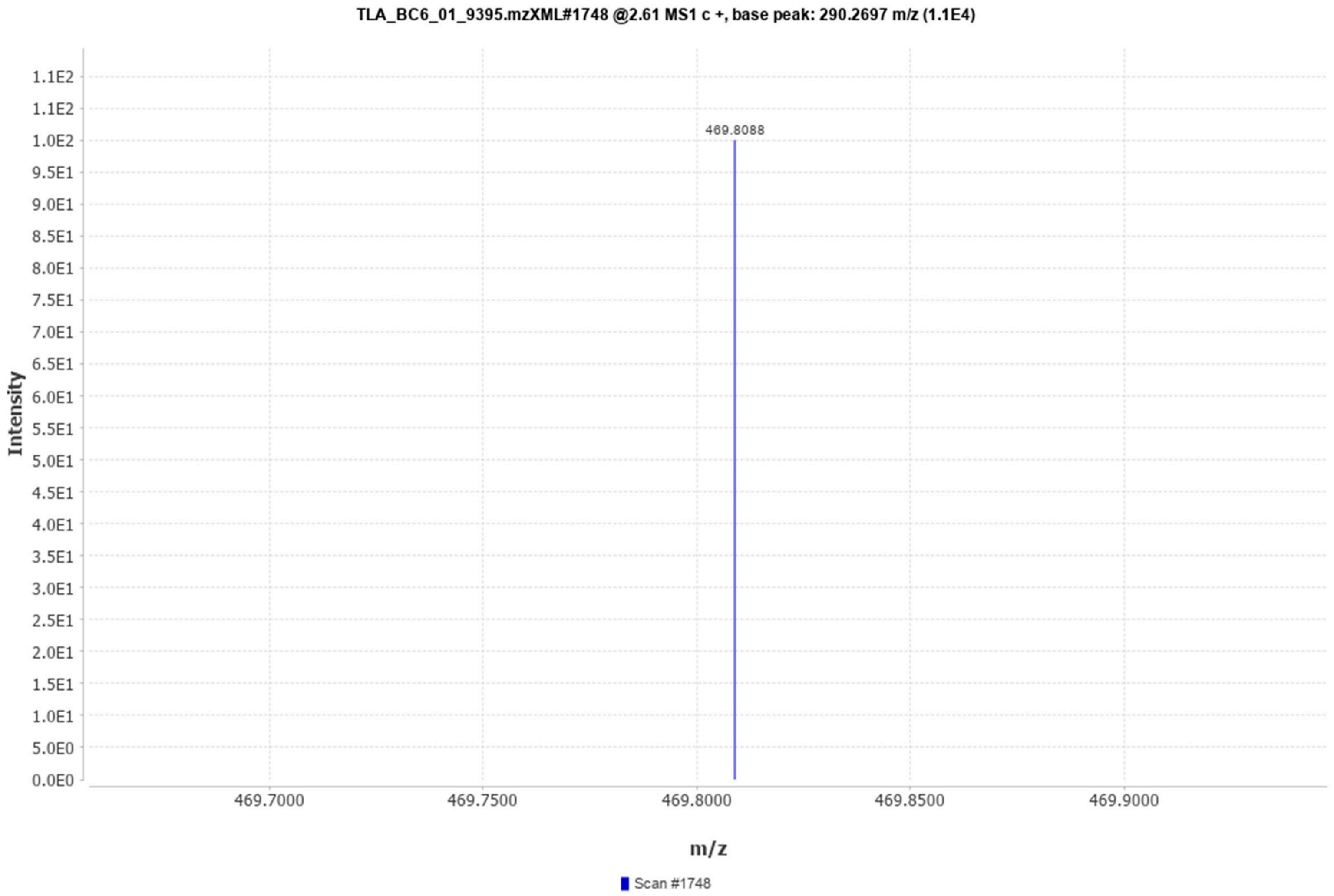

Figure SB.11: High-Resolution Electrospray Ionization Mass spectrum (HR-ESI-MS) of 3β Acetyl taraxerol [M+H]+ m/z = 469.8088

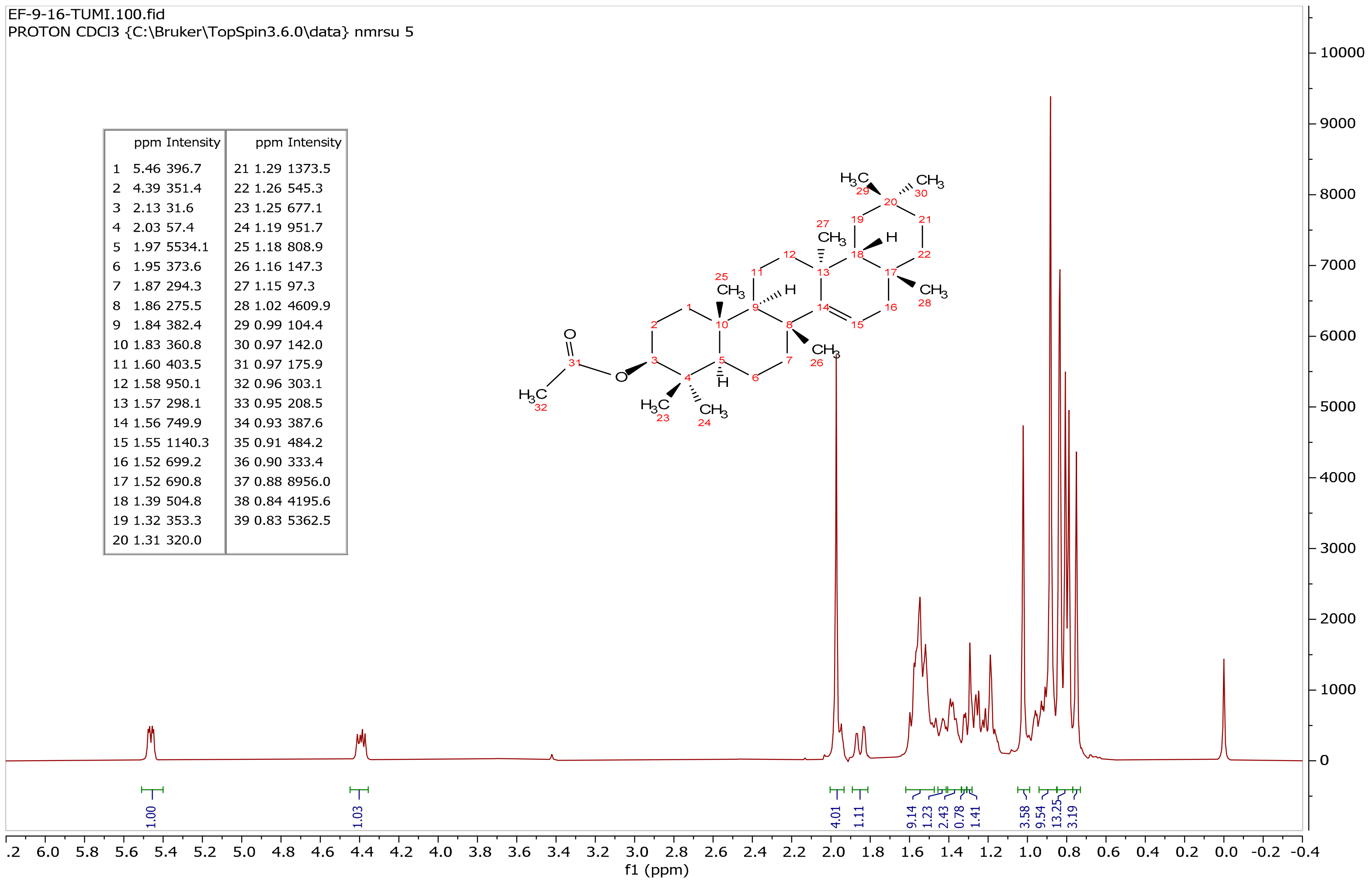

Figure SB.12: Proton Nuclear Magnetic Resonance (^{1}H NMR) spectrum of 3β Acetyl taraxerol ($CDCl_3$, 400 MHz)

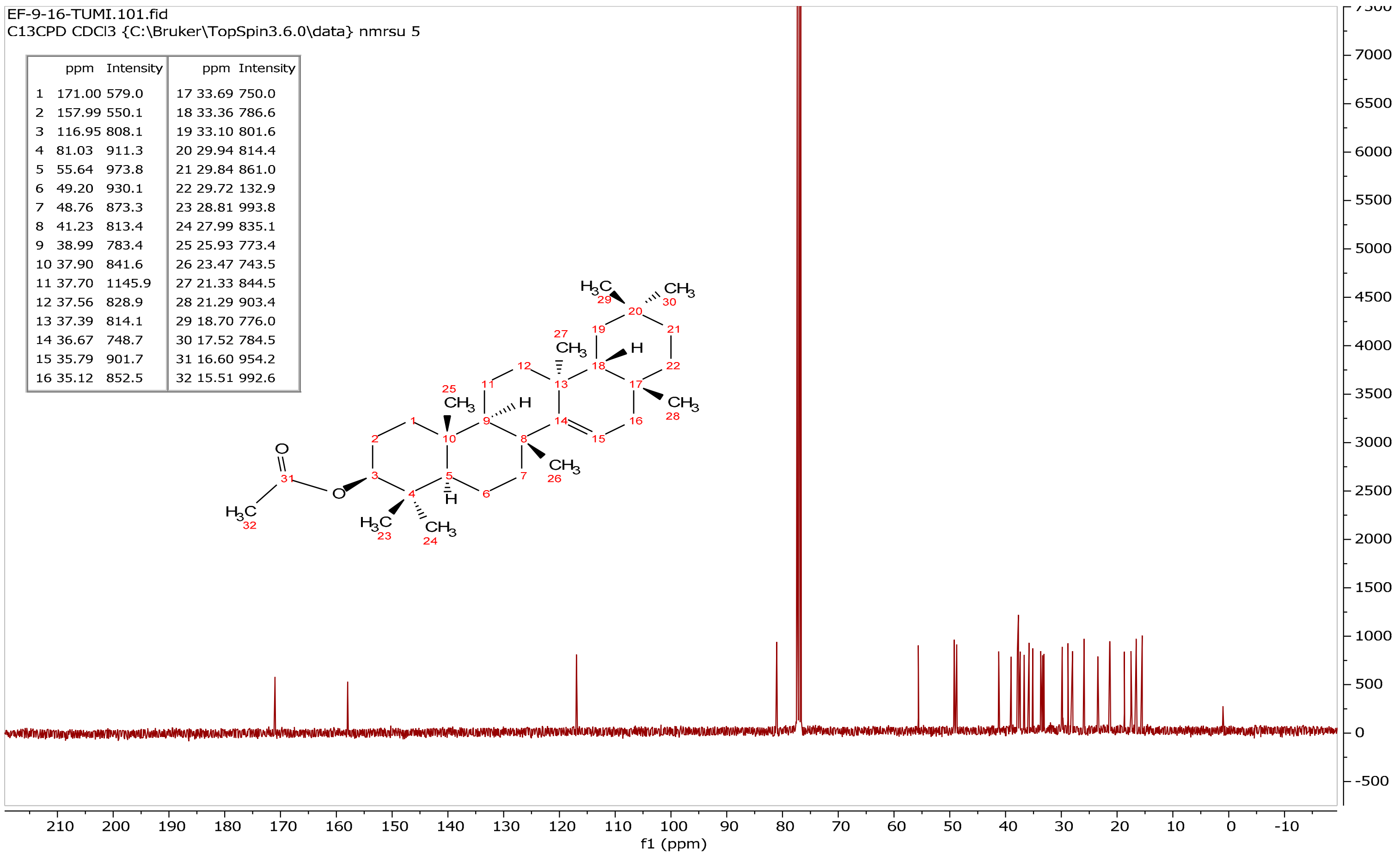

	ppm	Intensity		ppm	Intensity
1	171.00	579.0	17	33.69	750.0
2	157.99	550.1	18	33.36	786.6
3	116.95	808.1	19	33.10	801.6
4	81.03	911.3	20	29.94	814.4
5	55.64	973.8	21	29.84	861.0
6	49.20	930.1	22	29.72	132.9
7	48.76	873.3	23	28.81	993.8
8	41.23	813.4	24	27.99	835.1
9	38.99	783.4	25	25.93	773.4
10	37.90	841.6	26	23.47	743.5
11	37.70	1145.9	27	21.33	844.5
12	37.56	828.9	28	21.29	903.4
13	37.39	814.1	29	18.70	776.0
14	36.67	748.7	30	17.52	784.5
15	35.79	901.7	31	16.60	954.2
16	35.12	852.5	32	15.51	992.6

Figure SB.13: Carbon-13 Nuclear Magnetic Resonance (^{13}C NMR) spectrum of 3β Acetyl taraxerol ($CDCl_3$, 100 MHz)

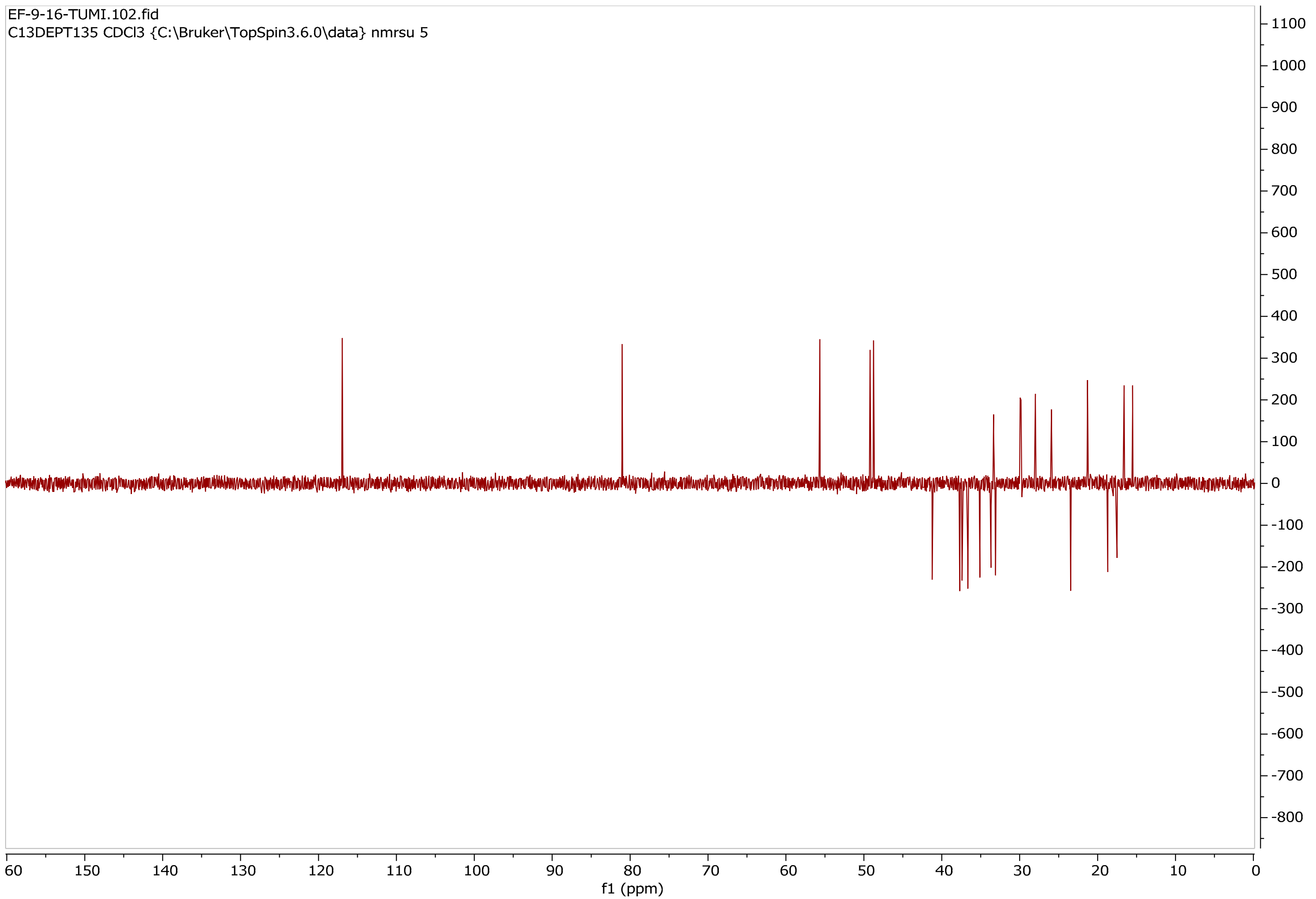

Figure SB.14: Distortionless Enhancement by Polarization Transfer (DEPT) NMR spectra of 3β Acetyl taraxerol ($CDCl_3$, 100 MHz)

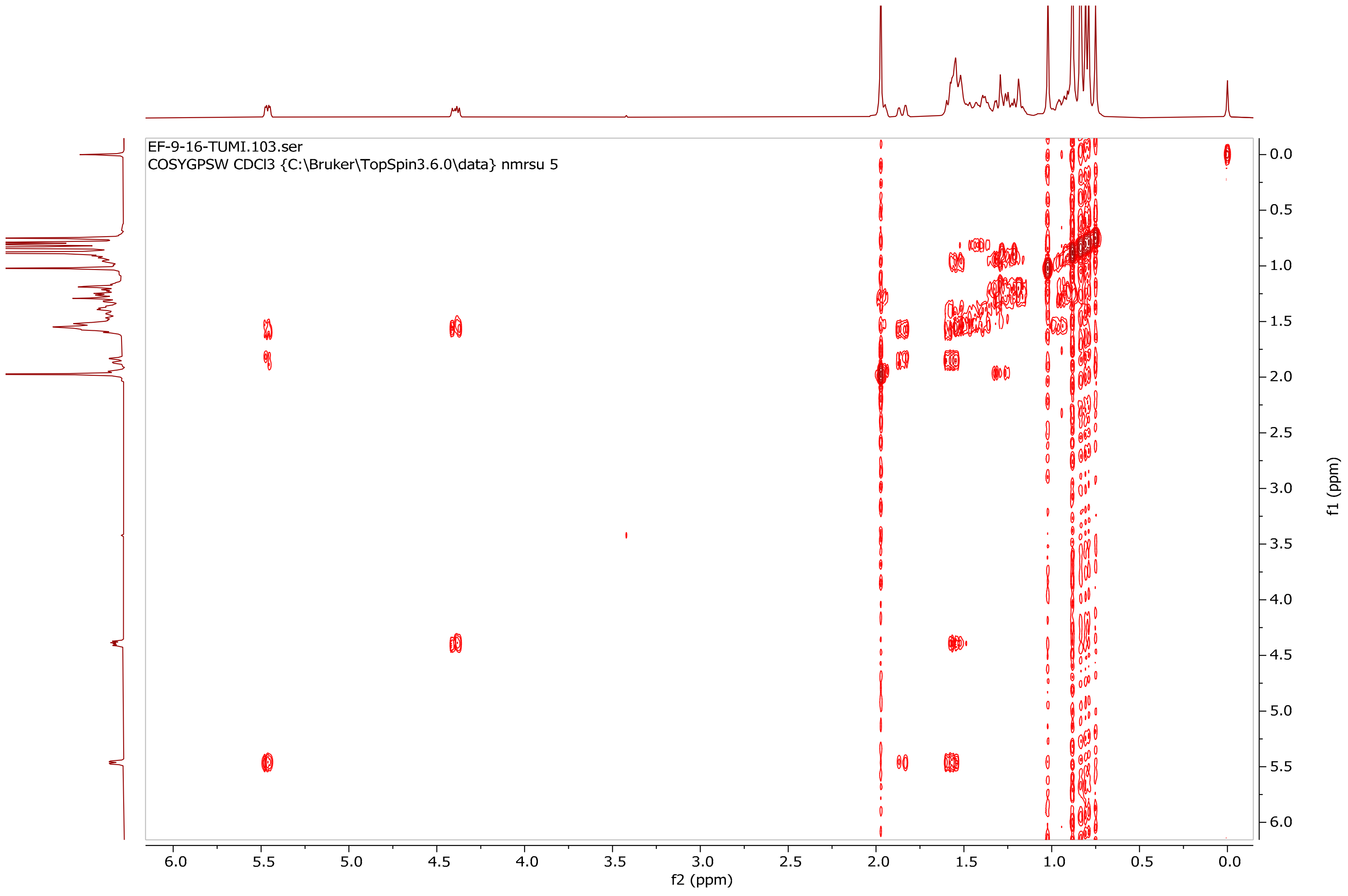

Figure SB.15: Gradient Correlated (gCOSY) spectrum of 3β Acetyl taraxerol

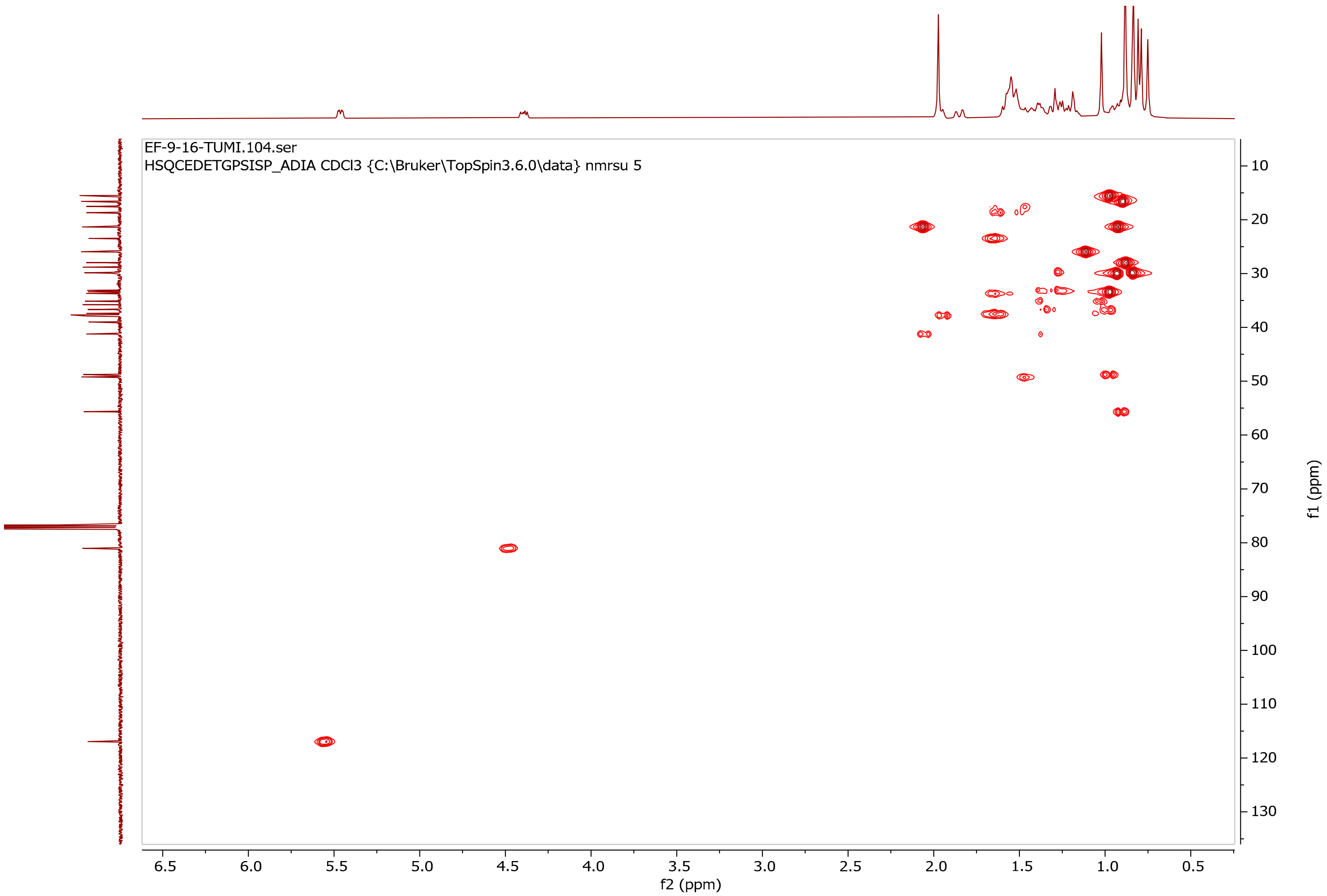

Figure SB.16: Gradient Heteronuclear Single Quantum Coherence (gHSQC) spectrum of 3β Acetyl taraxerol

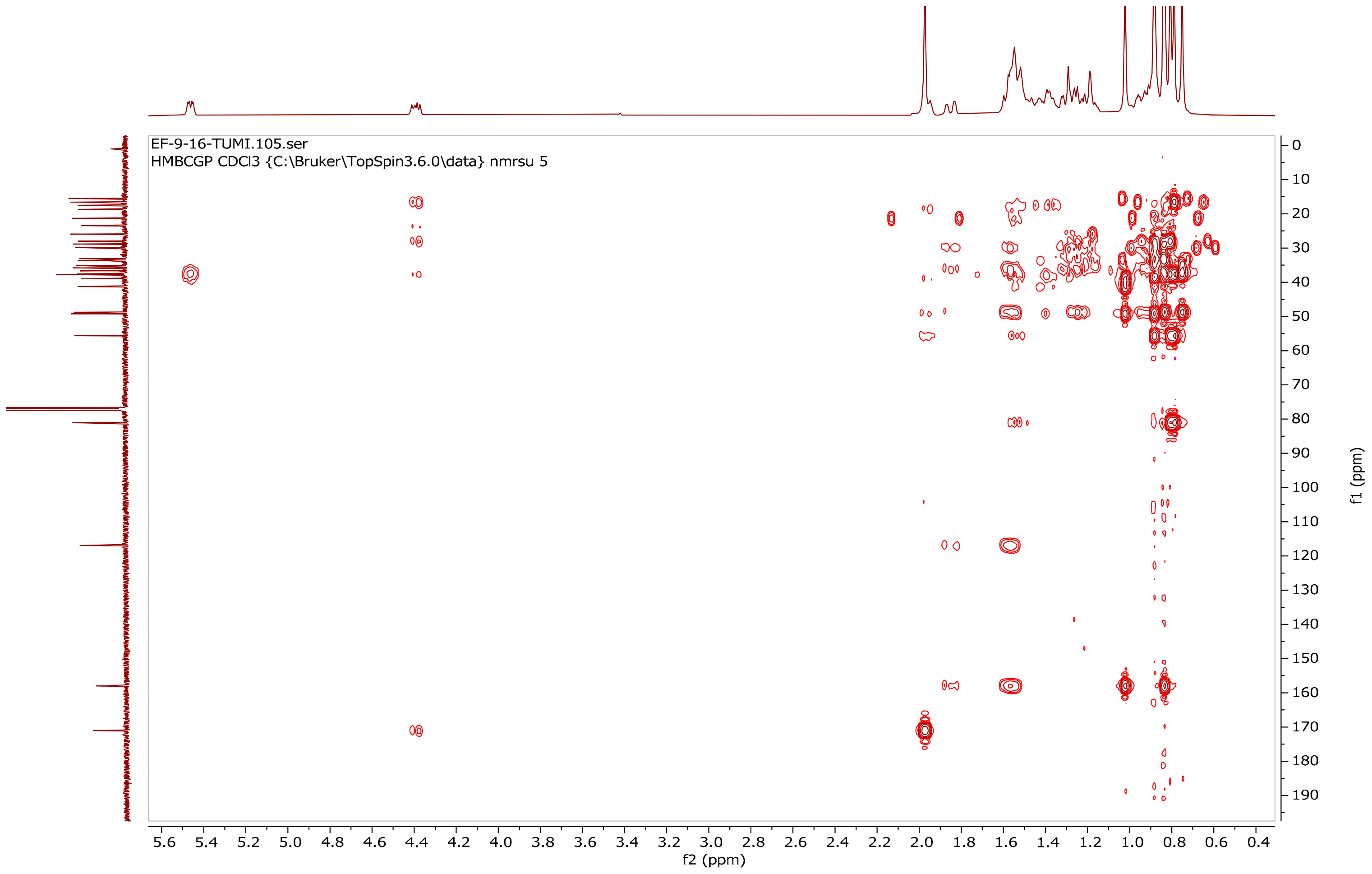

Figure SB.17: Gradient Heteronuclear Multiple Bond Quantum Coherence (gHMBC) spectrum of 3β Acetyl taraxerol

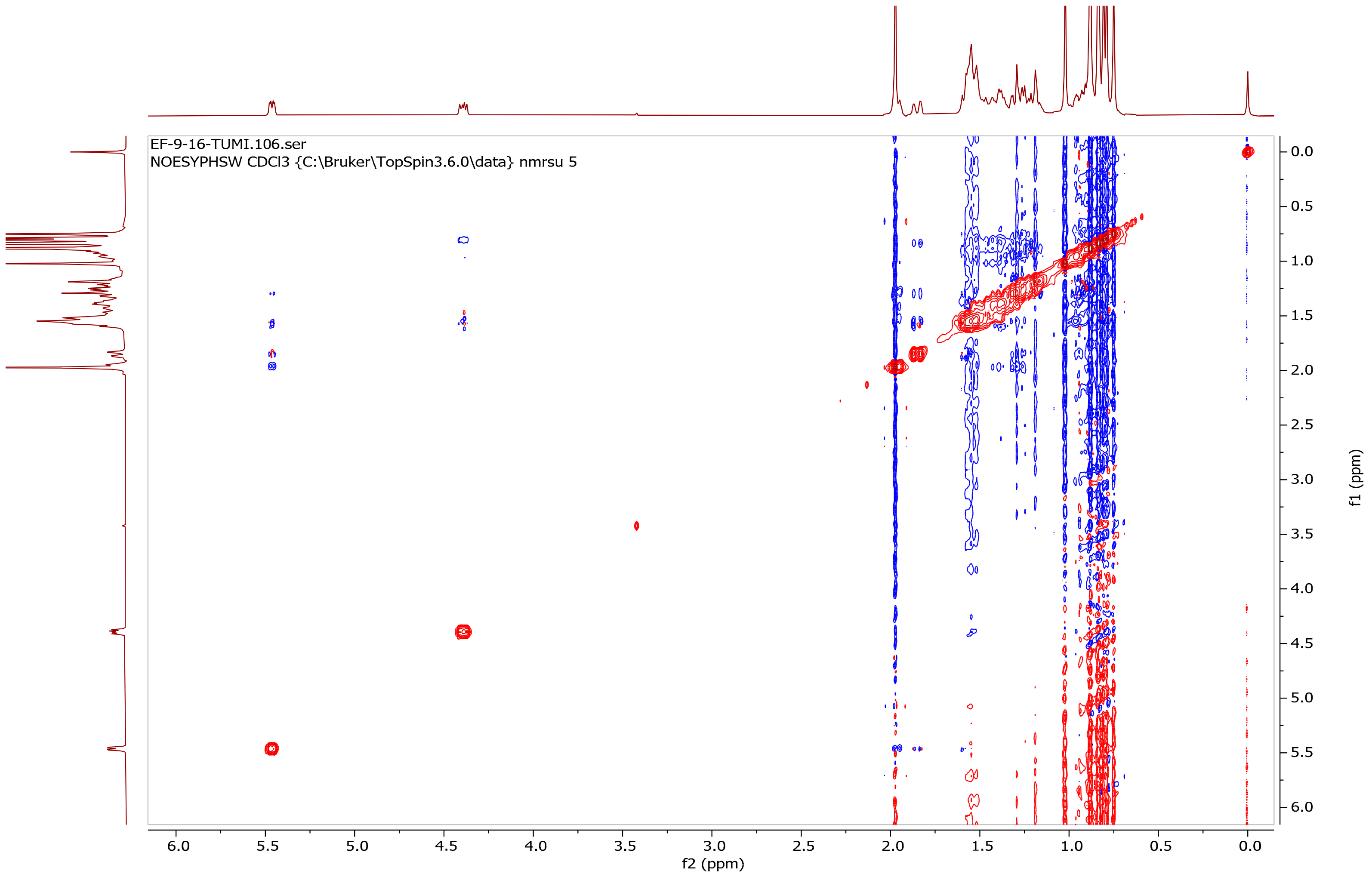

Figure SB.18: Nuclear Overhauser Effect Spectroscopy (NOESY) spectrum of 3β Acetyl taraxerol

3. Spectral data of Compound C

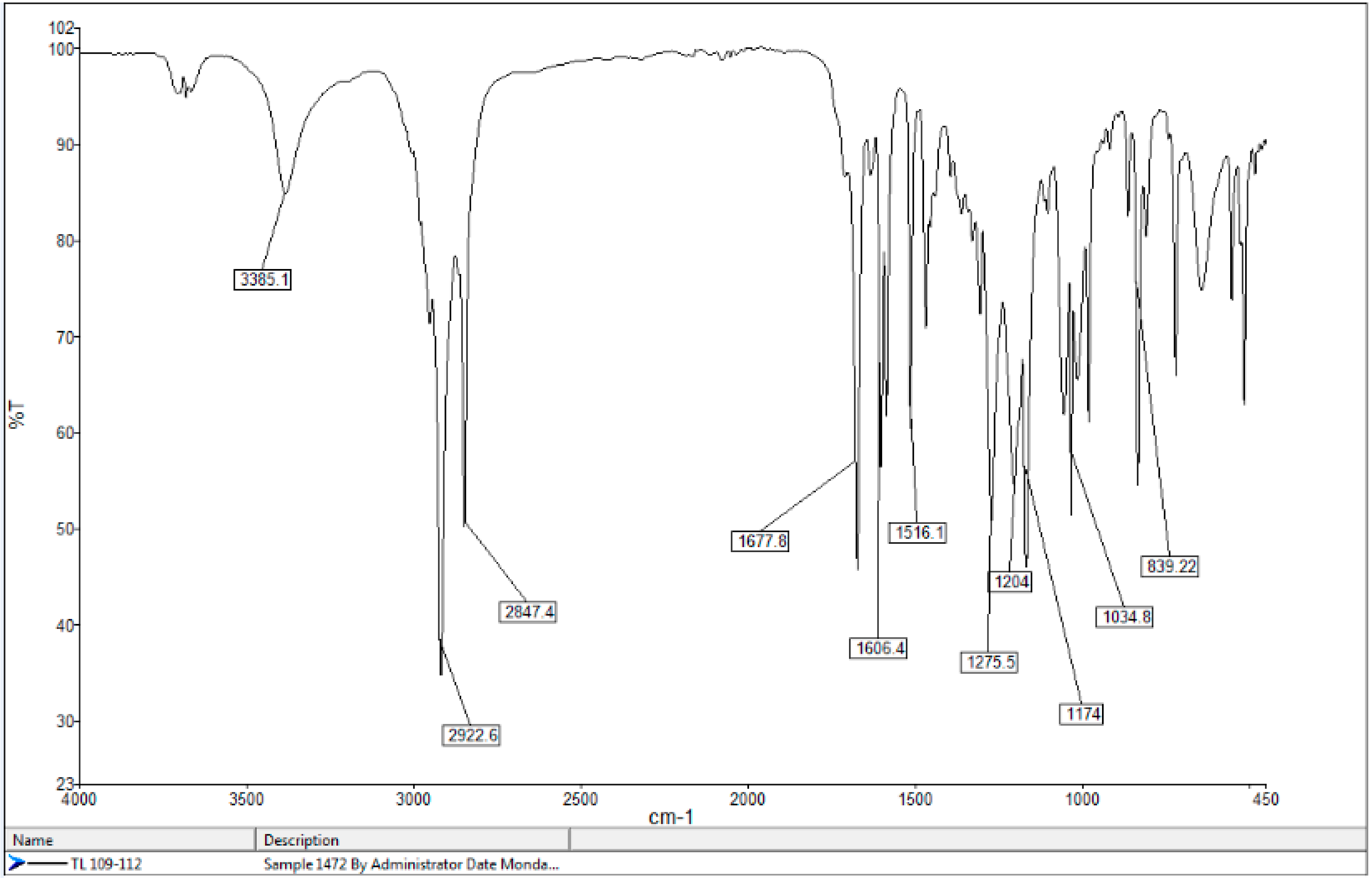

Figure SC.19: Fourier-Transform Infrared Spectroscopy (FTIR) spectrum of Dodecyl P Coumarate [Dodecyl (*E*)-3-(4-hydroxyphenyl) prop-2-enoate]

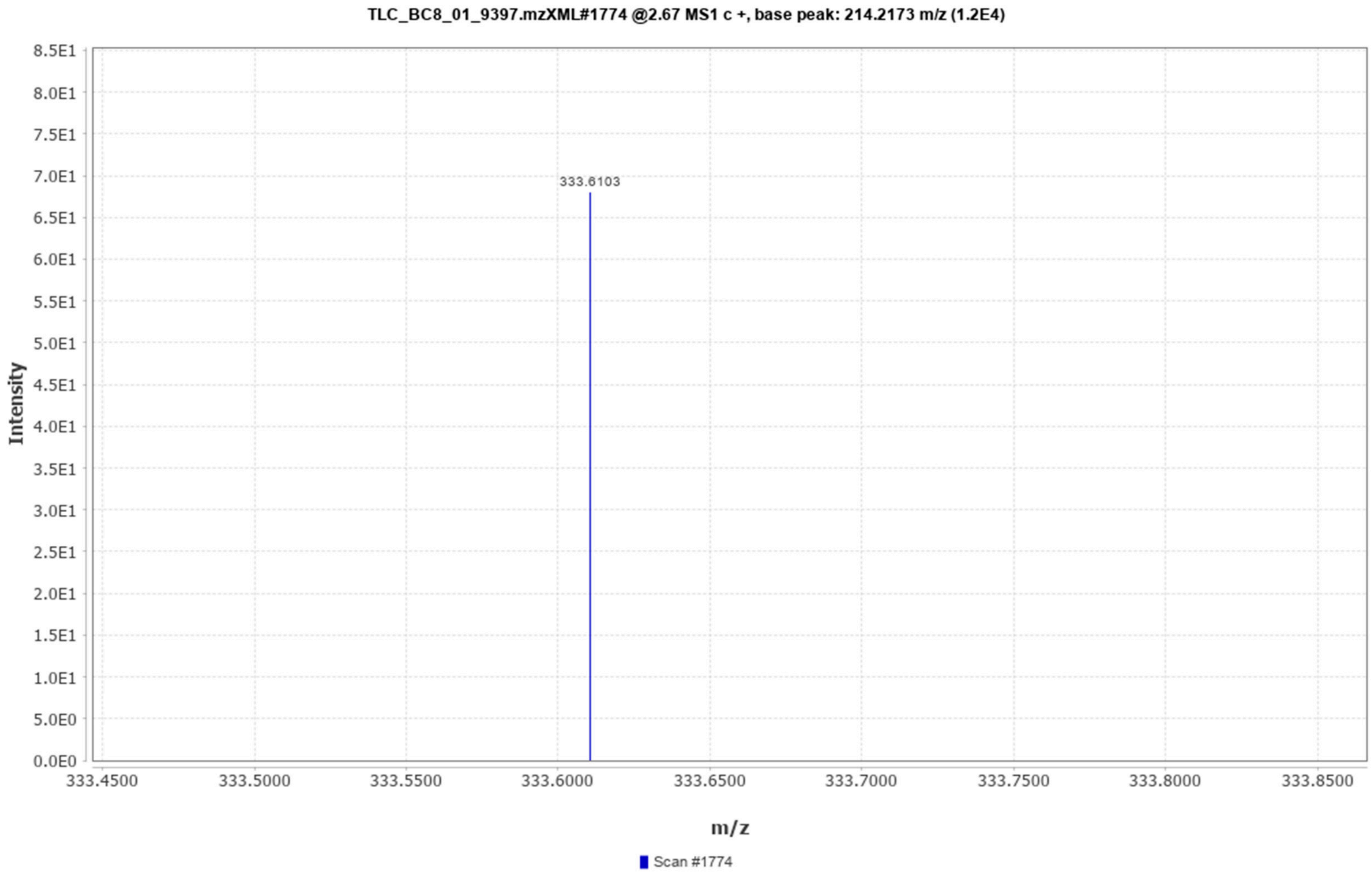

Figure SC.20: High-Resolution Electrospray Ionization Mass spectrum (HR-ESI-MS) of Dodecyl P Coumarate $[M+H]^+$ m/z = 333.6103

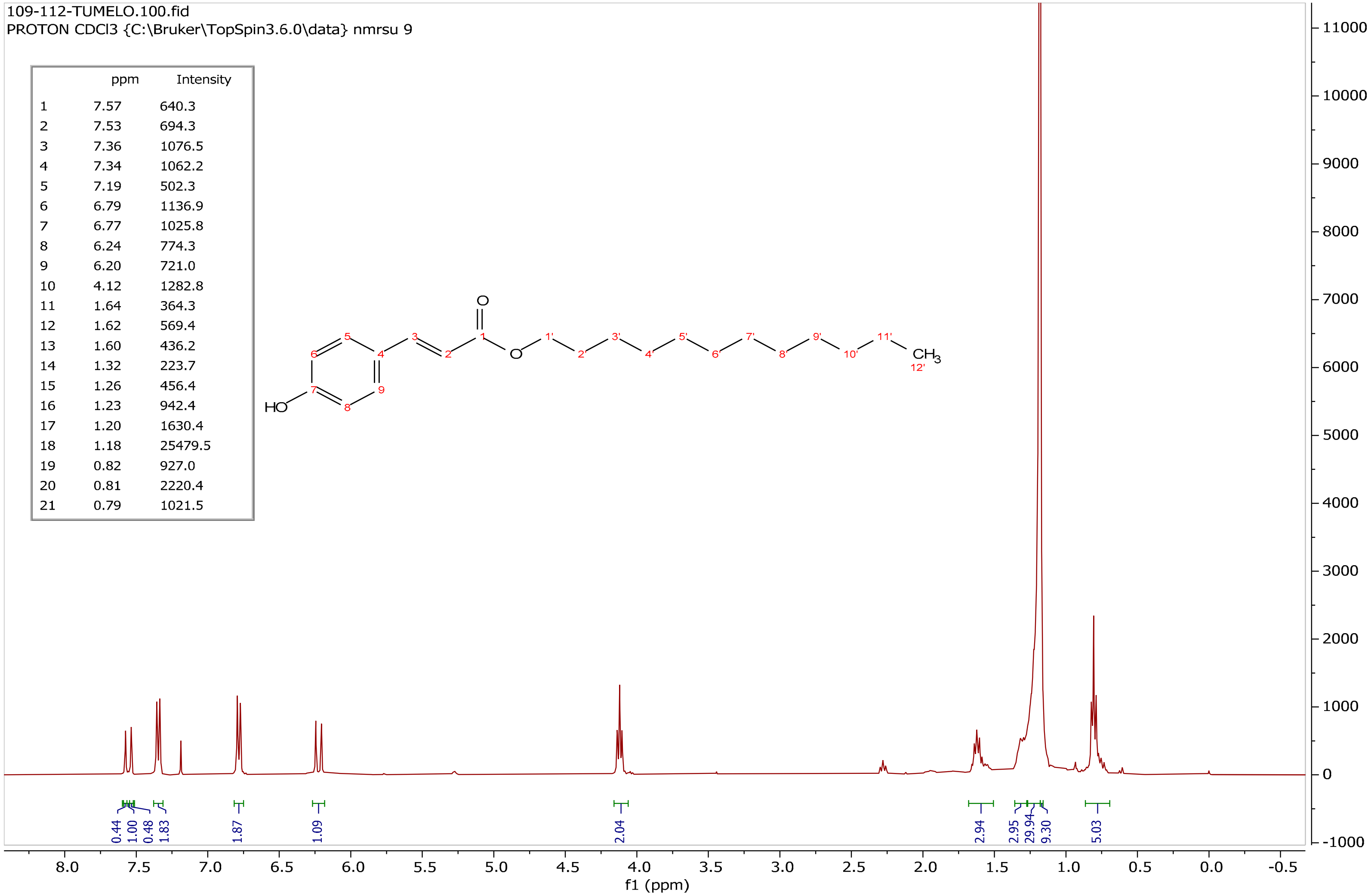

Figure SC.21: Proton Nuclear Magnetic Resonance (^1H NMR) spectrum of Dodecyl P Coumarate ($CDCl_3$, 400 MHz)

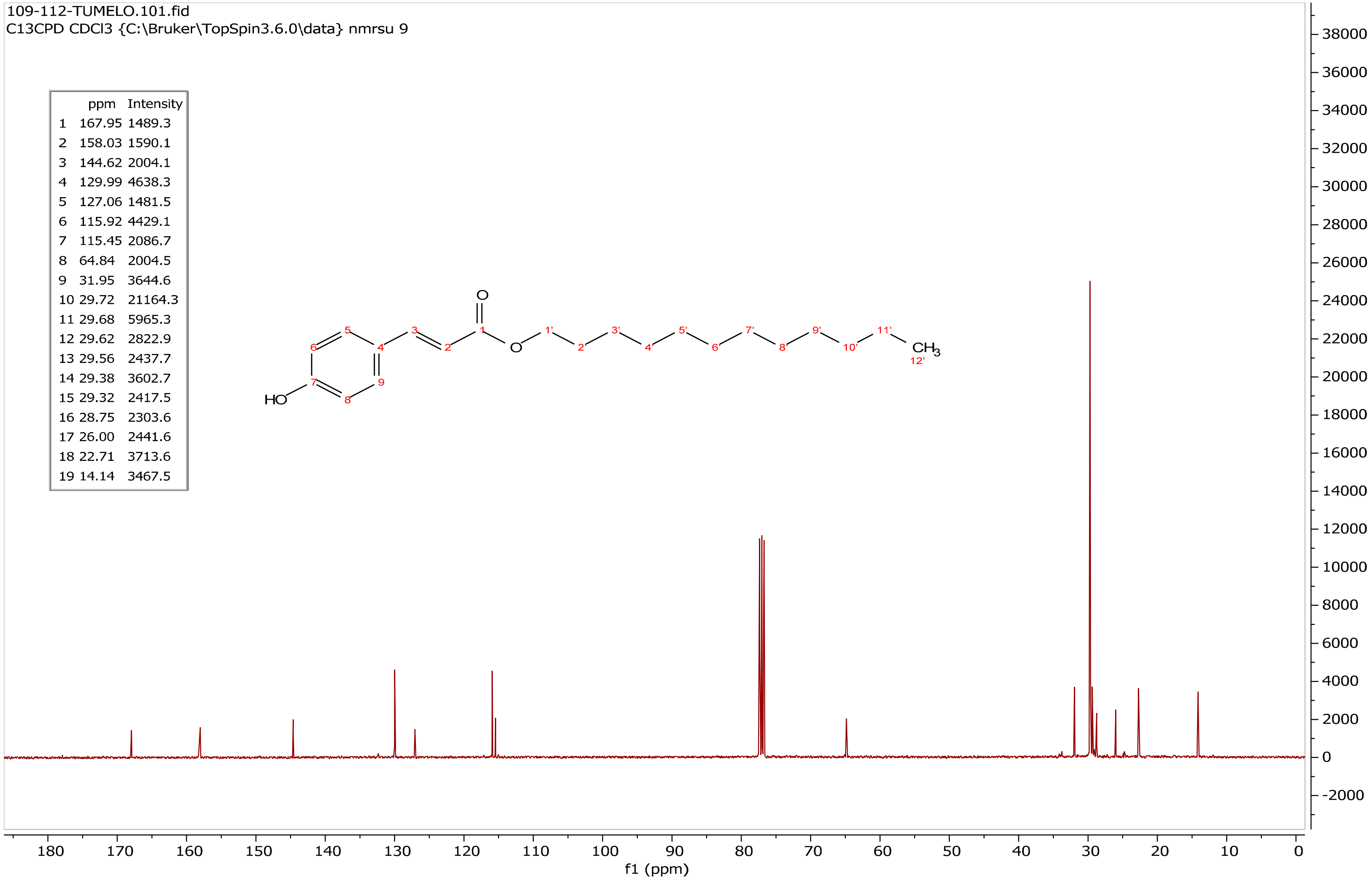

Figure SC.22: Carbon-13 Nuclear Magnetic Resonance (^{13}C NMR) spectrum of Dodecyl P Coumarate ($CDCl_3$, 100 MHz)

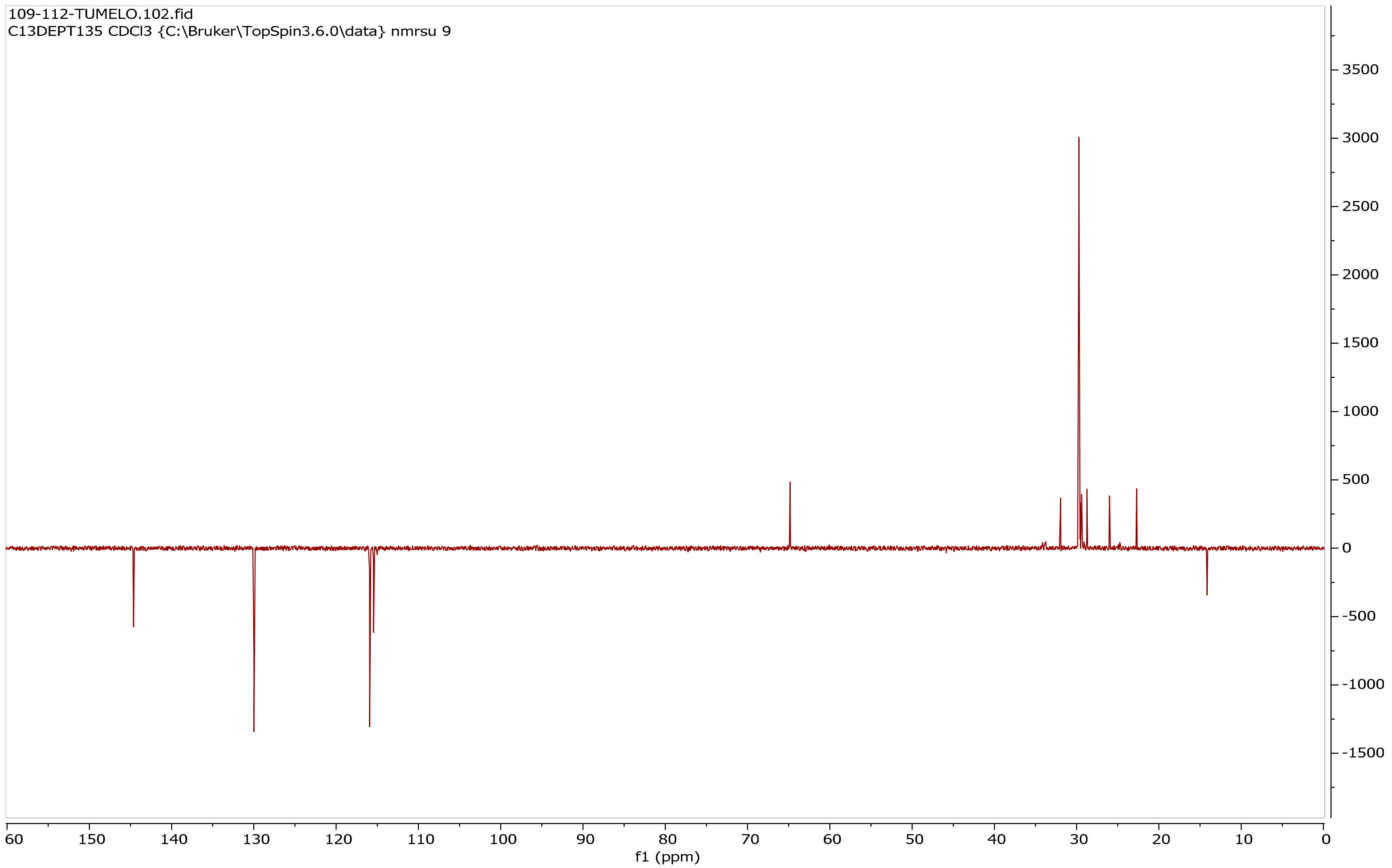

Figure SC.23: Distortionless Enhancement by Polarization Transfer (DEPT) NMR spectra of Dodecyl P Coumarate ($CDCl_3$, 100 MHz)

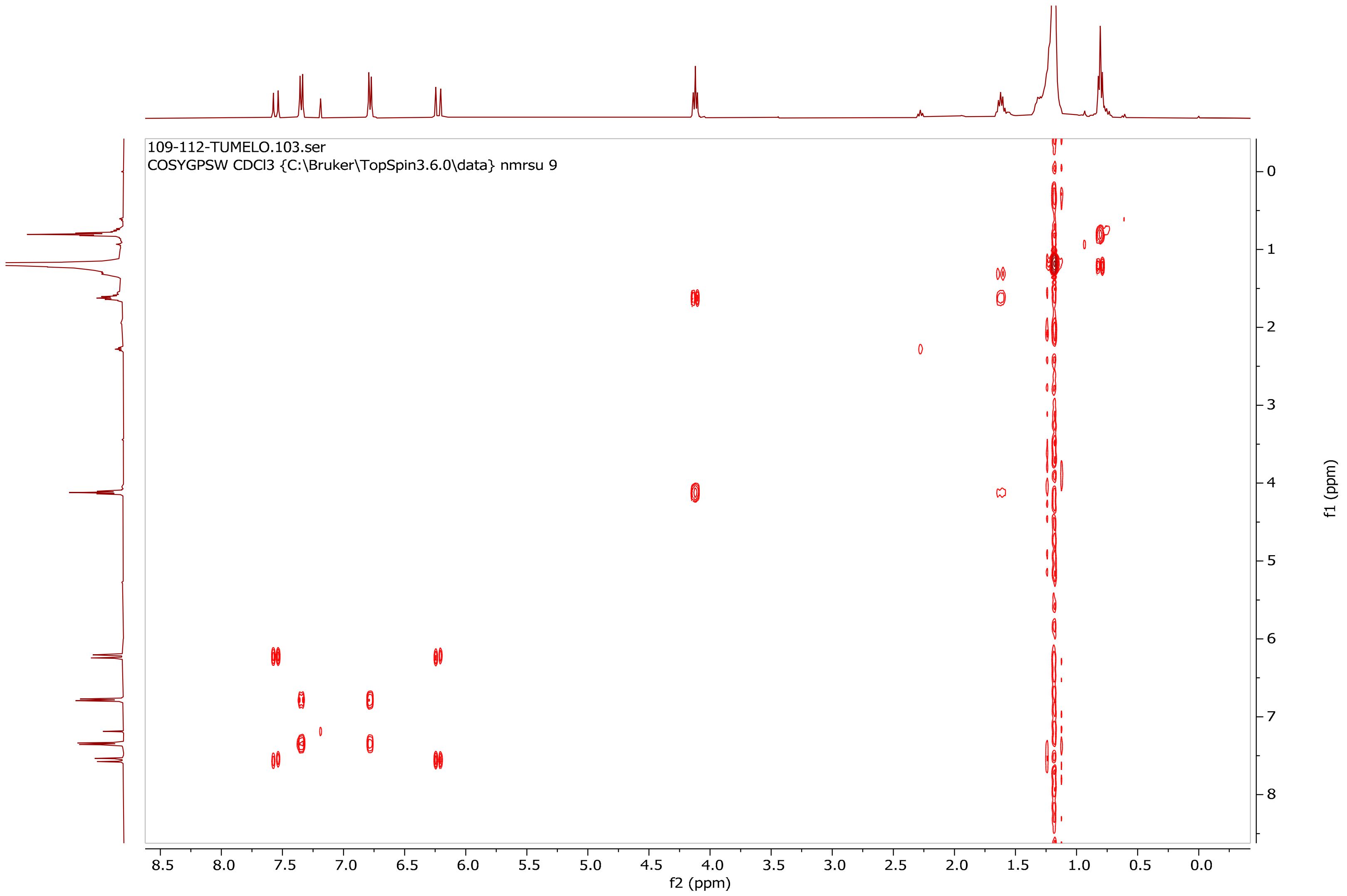

Figure SC.24: Gradient Correlated (gCOSY) spectrum of Dodecyl P Coumarate

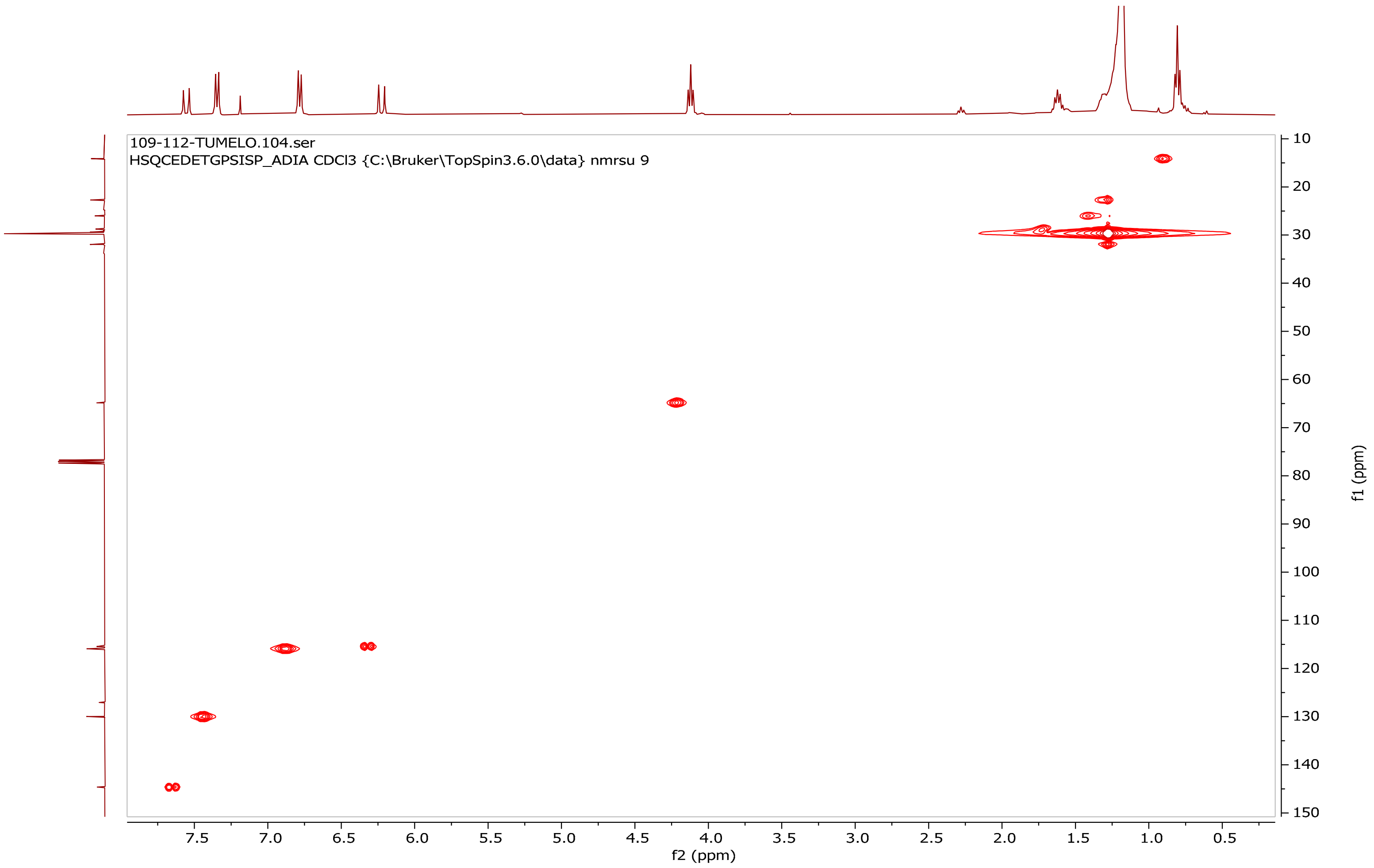

Figure SC.25: Gradient Heteronuclear Single Quantum Coherence (gHSQC) spectrum of Dodecyl P Coumarate

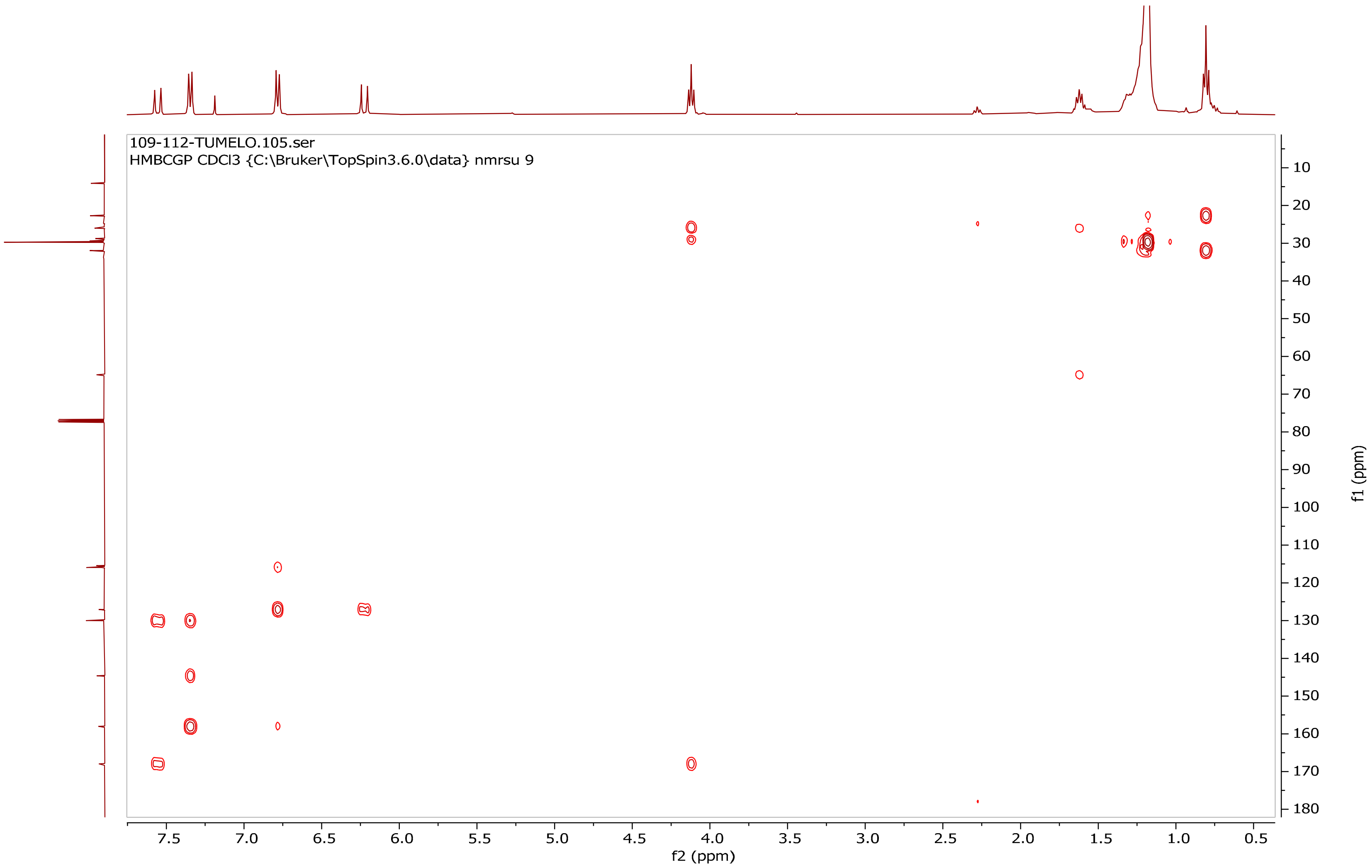

Figure SC.26: Gradient Heteronuclear Multiple Bond Quantum Coherence (gHMBC) spectrum of Dodecyl P Coumarate

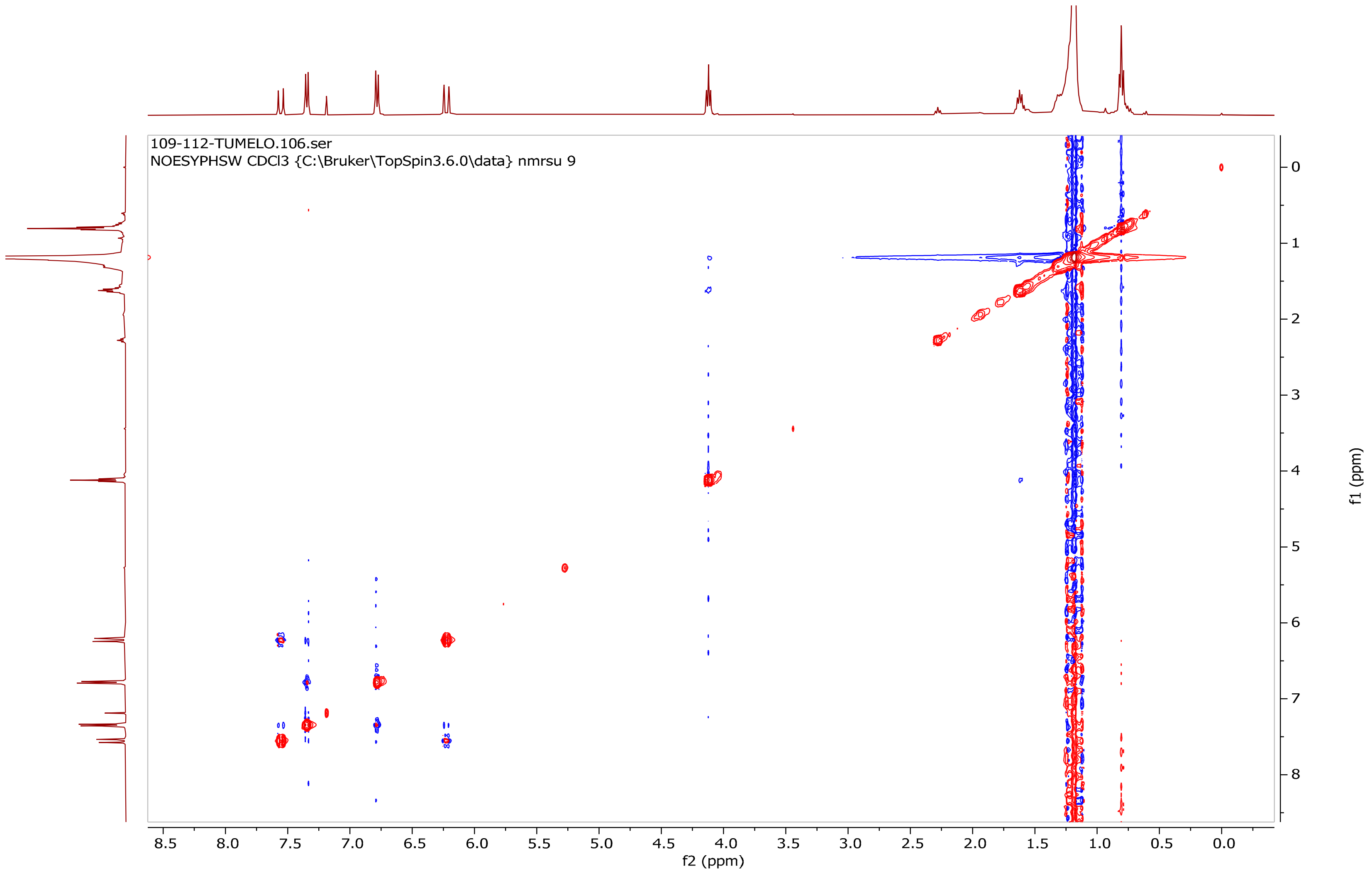

Figure SC.27: Nuclear Overhauser Effect Spectroscopy (NOESY) spectrum of Dodecyl P Coumarate

4. **Spectral data of Compound D**

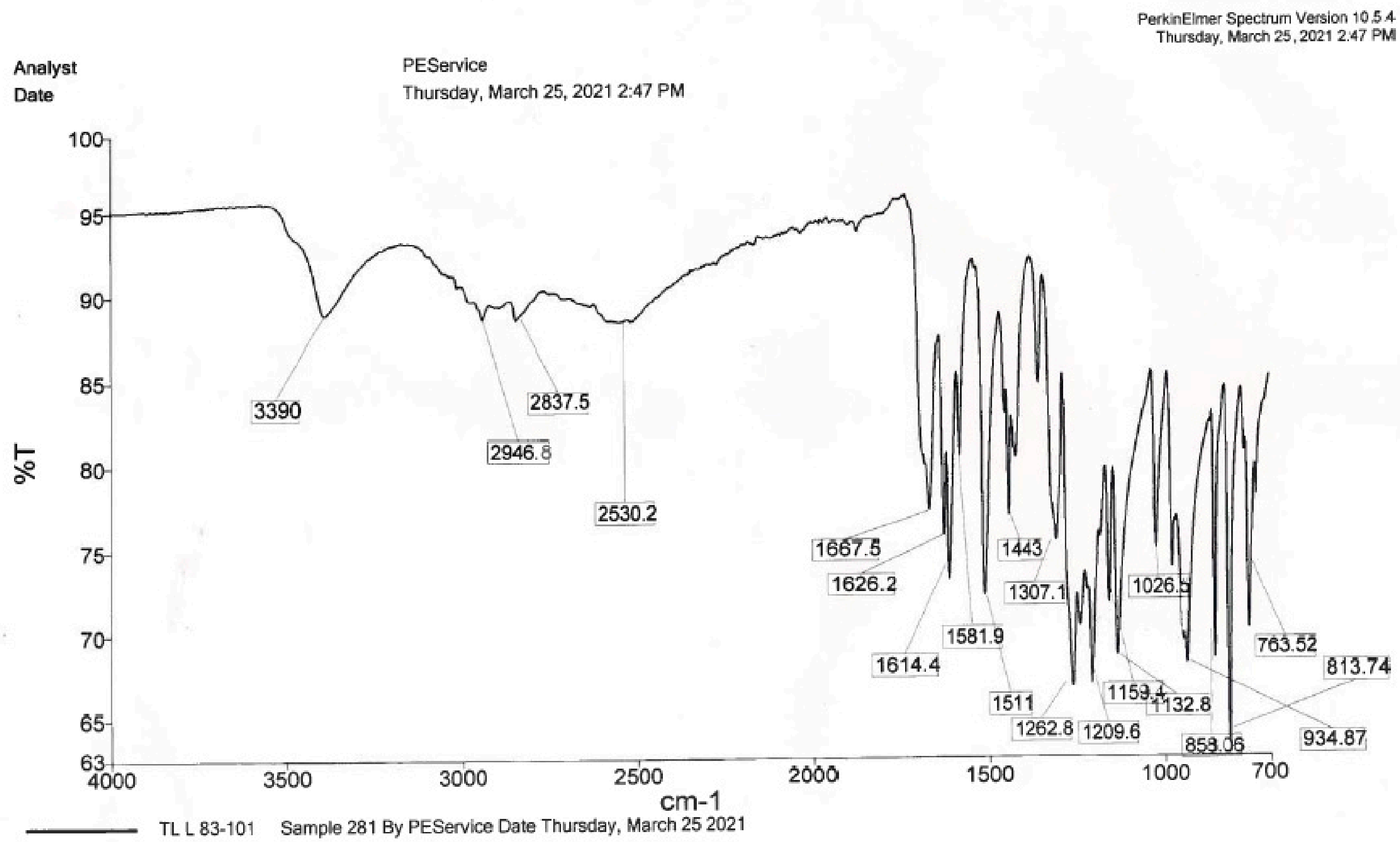

Figure SD.28: Fourier-Transform Infrared Spectroscopy (FTIR) spectrum of Ferulic acid [(*E*)-3-(4-hydroxy-3-methoxyphenyl) prop-2-enoic acid]

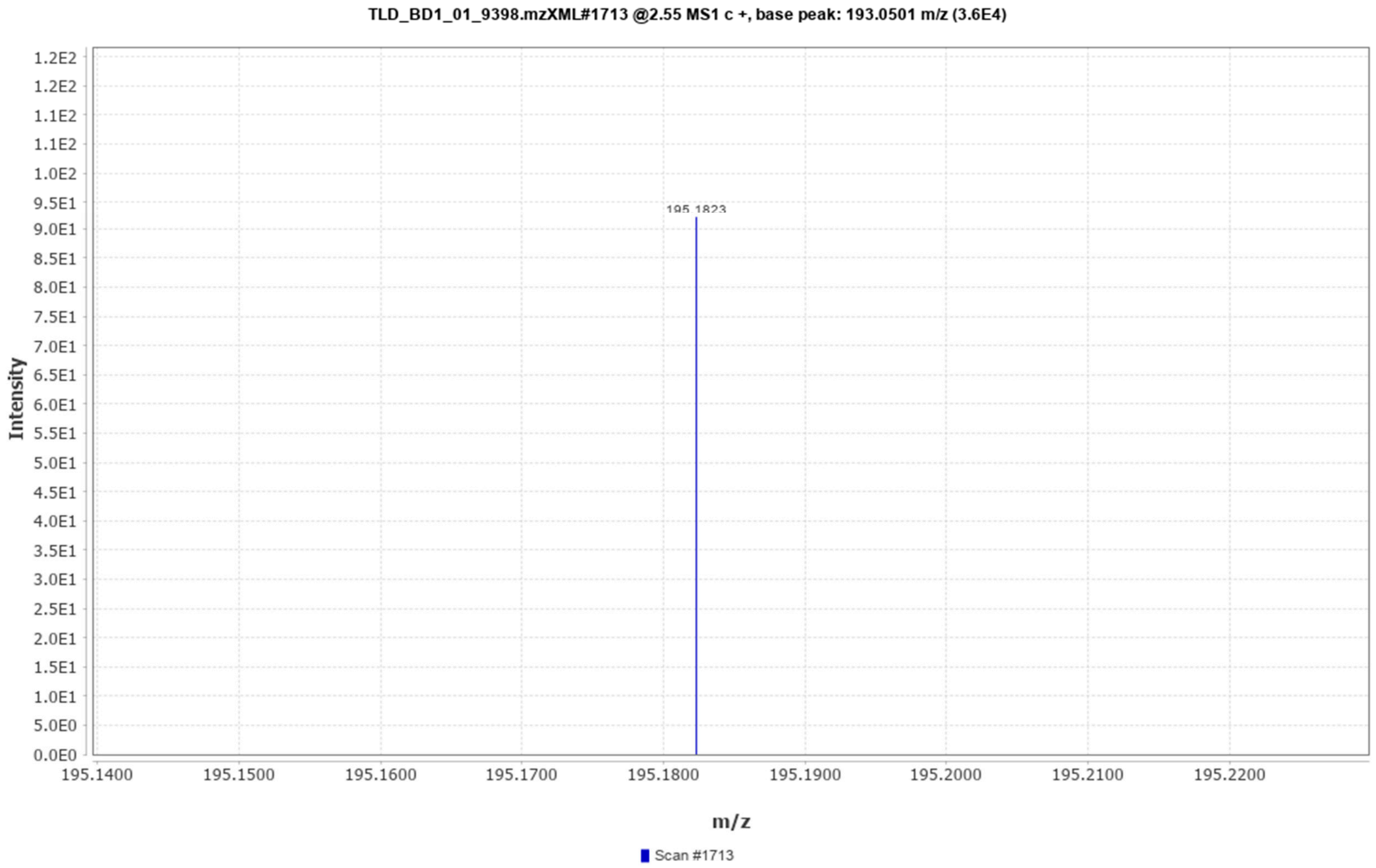

Figure SD.29: High-Resolution Electrospray Ionization Mass spectrum (HR-ESI-MS) of Ferulic Acid [M+H]$^+$ m/z = 195.1823

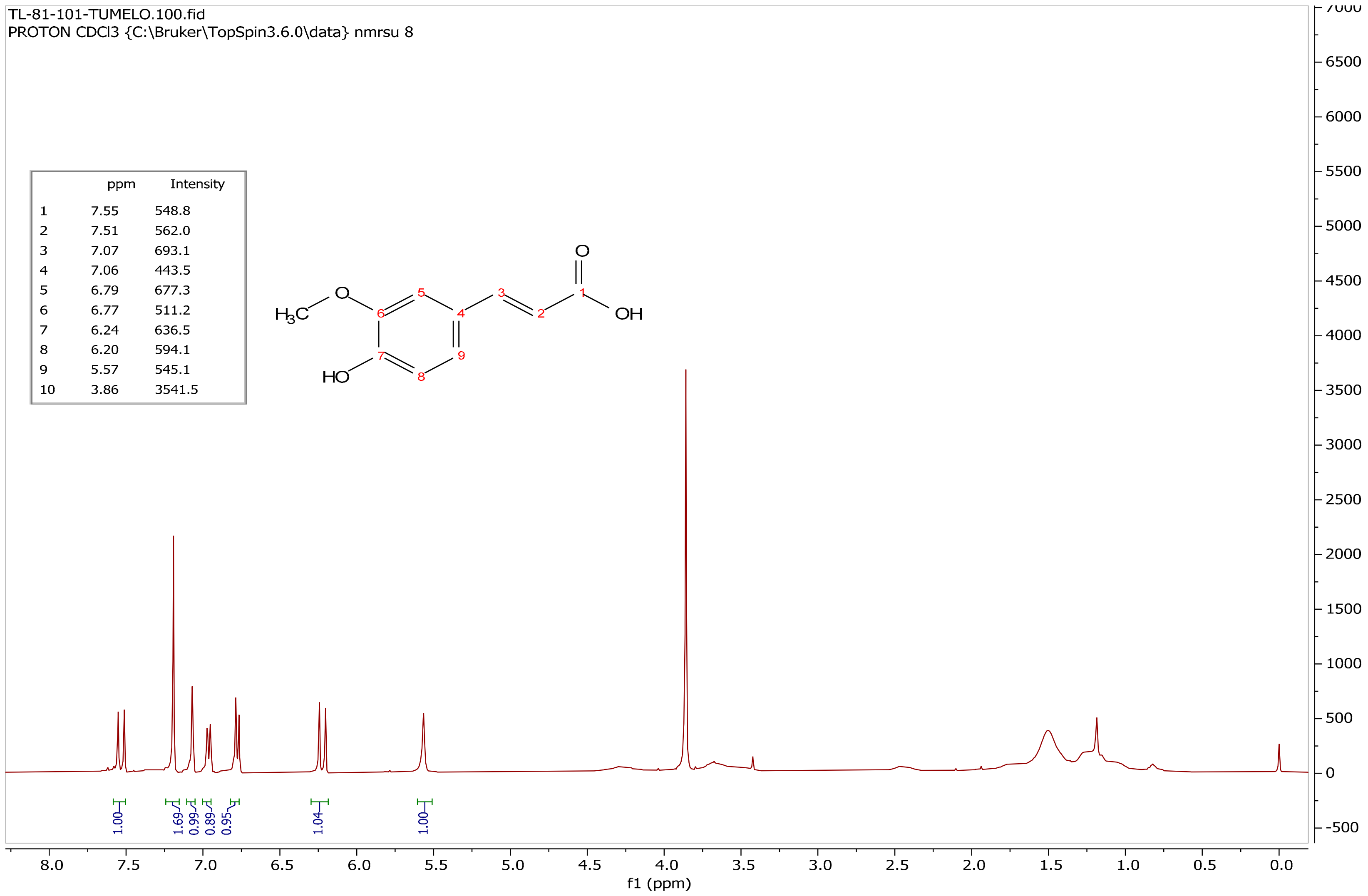

Figure SD.30: Proton Nuclear Magnetic Resonance (^{1}H NMR) spectrum of Ferulic Acid ($CDCl_3$, 400 MHz)

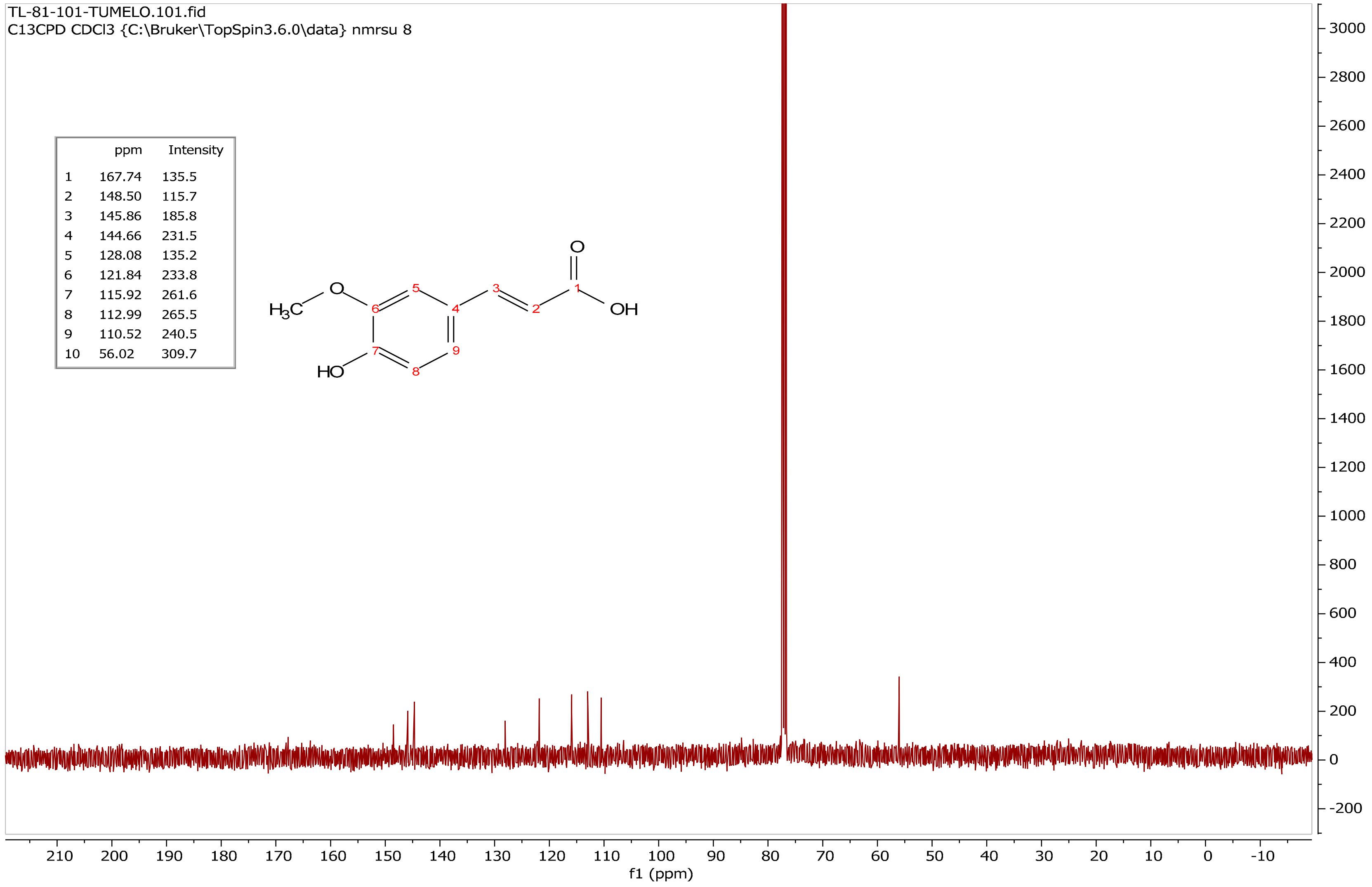

Figure SD.31: Carbon-13 Nuclear Magnetic Resonance (^{13}C NMR) spectrum of Dodecyl P Coumarate ($CDCl_3$, 100 MHz)

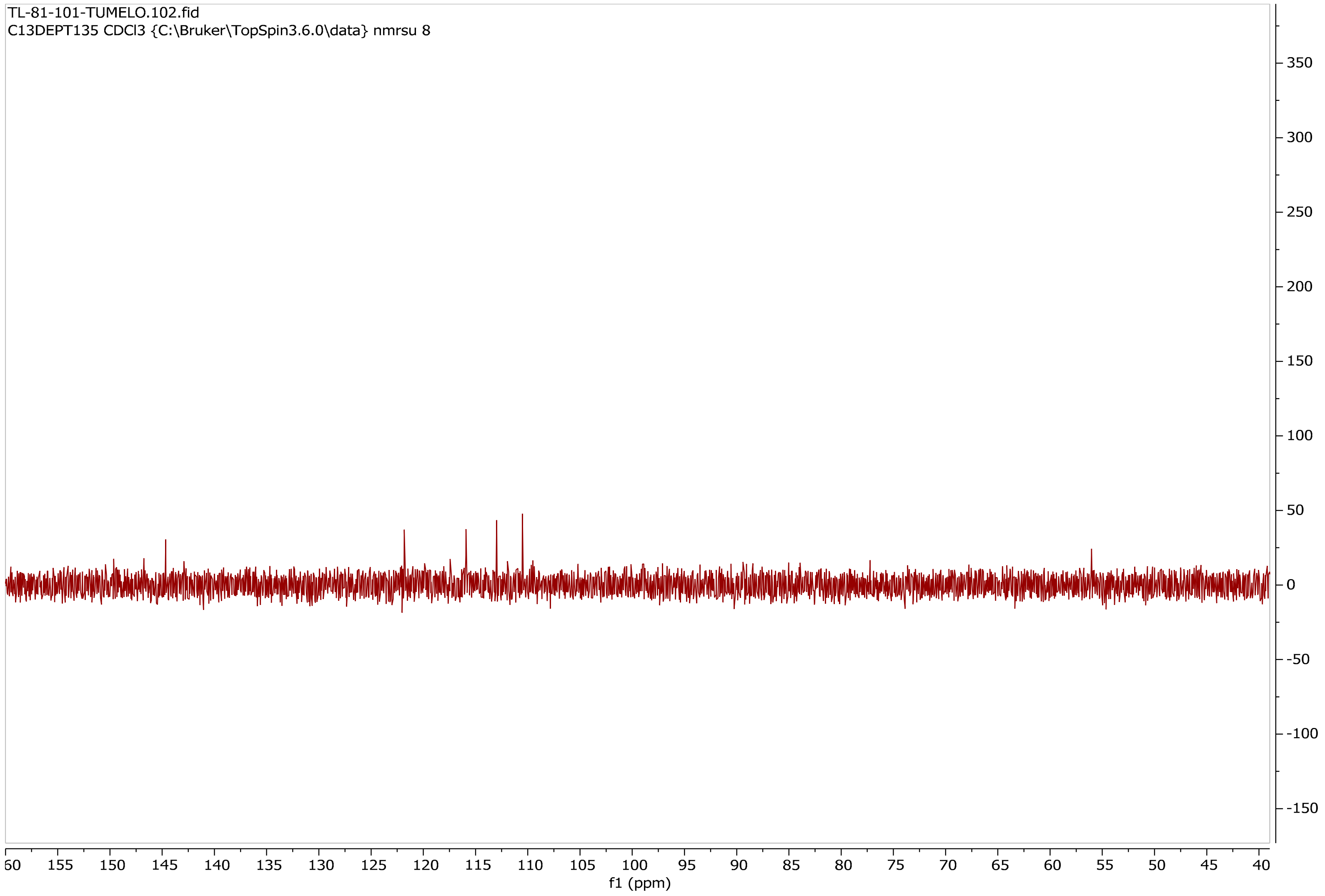

Figure SD.32: Distortionless Enhancement by Polarization Transfer (DEPT) NMR spectra of Ferulic Acid ($CDCl_3$, 100 MHz)

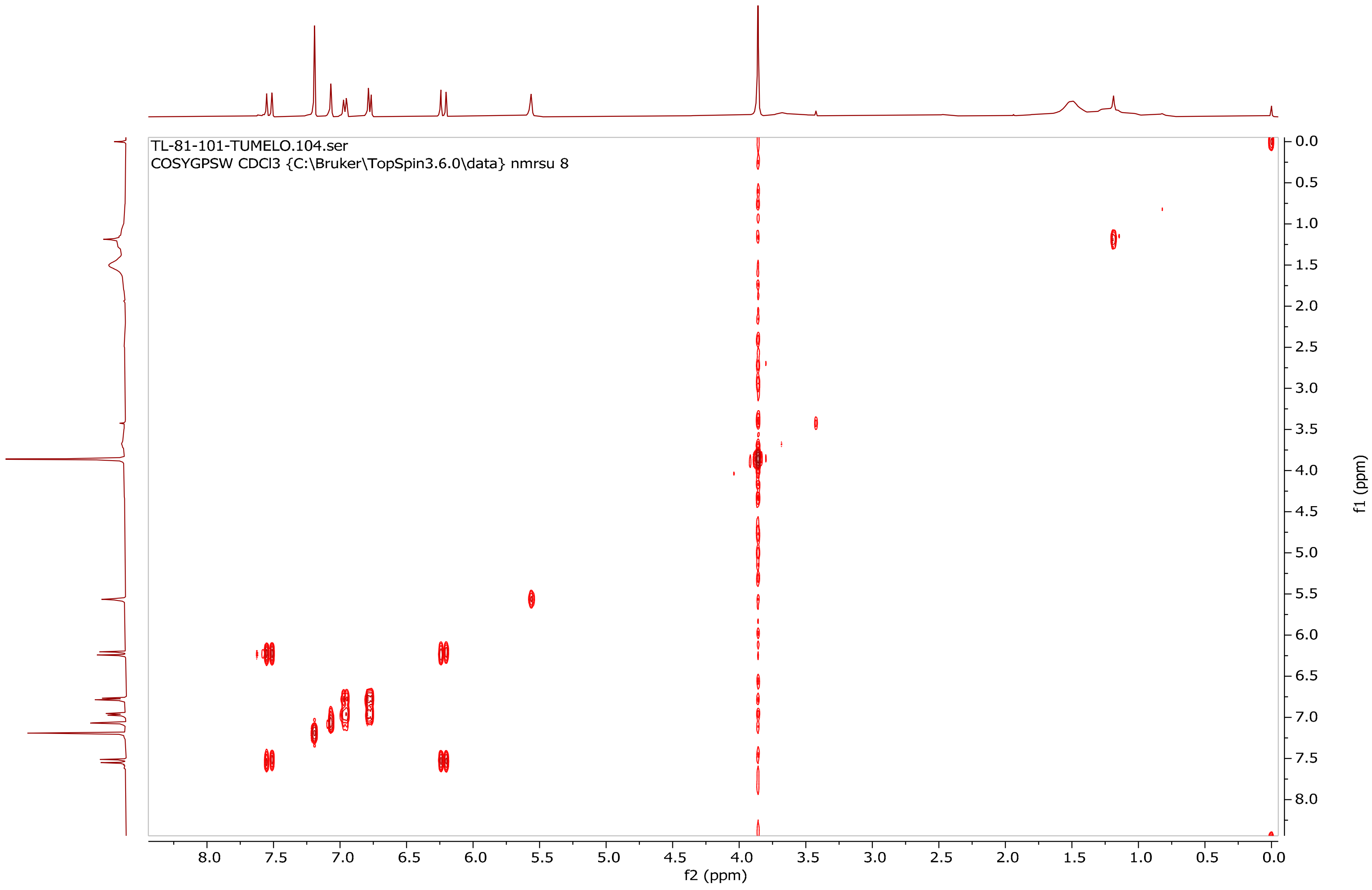

Figure SD.33: Gradient Correlated (gCOSY) spectrum of Ferulic Acid

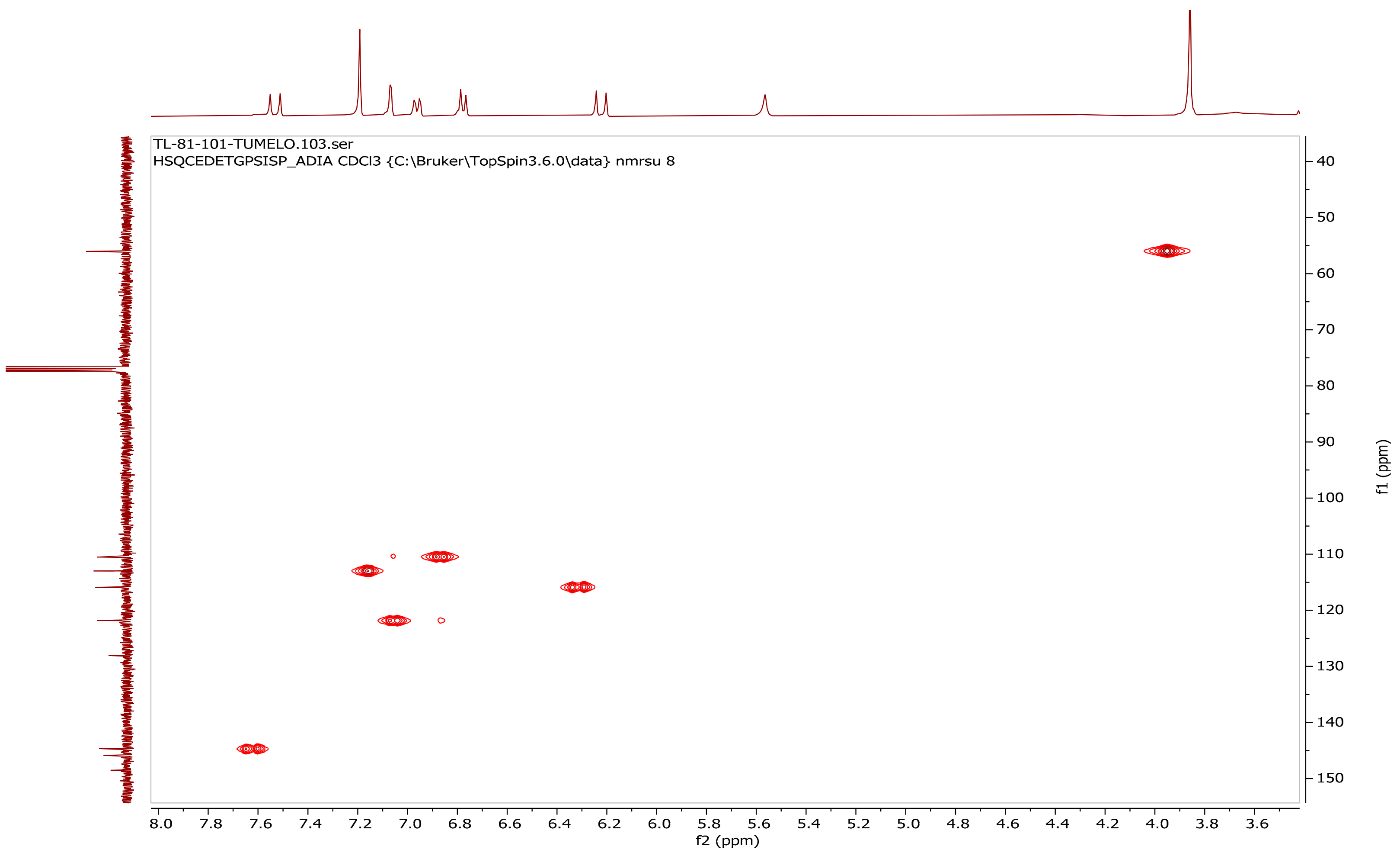

Figure SD.34: Gradient Heteronuclear Single Quantum Coherence (gHSQC) spectrum of Ferulic Acid

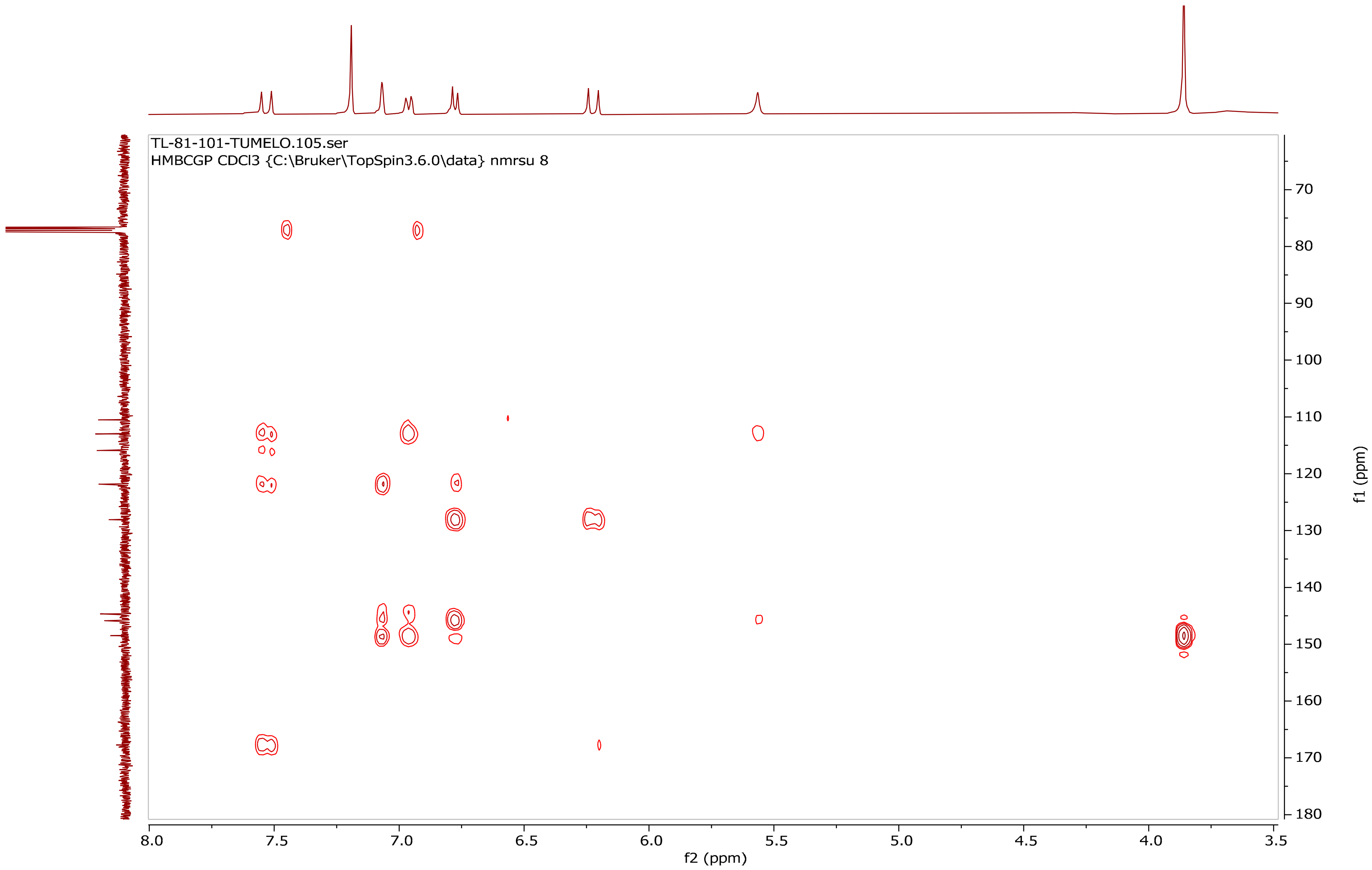

Figure SD.35: Gradient Heteronuclear Multiple Bond Quantum Coherence (gHMBC) spectrum of Ferulic Acid

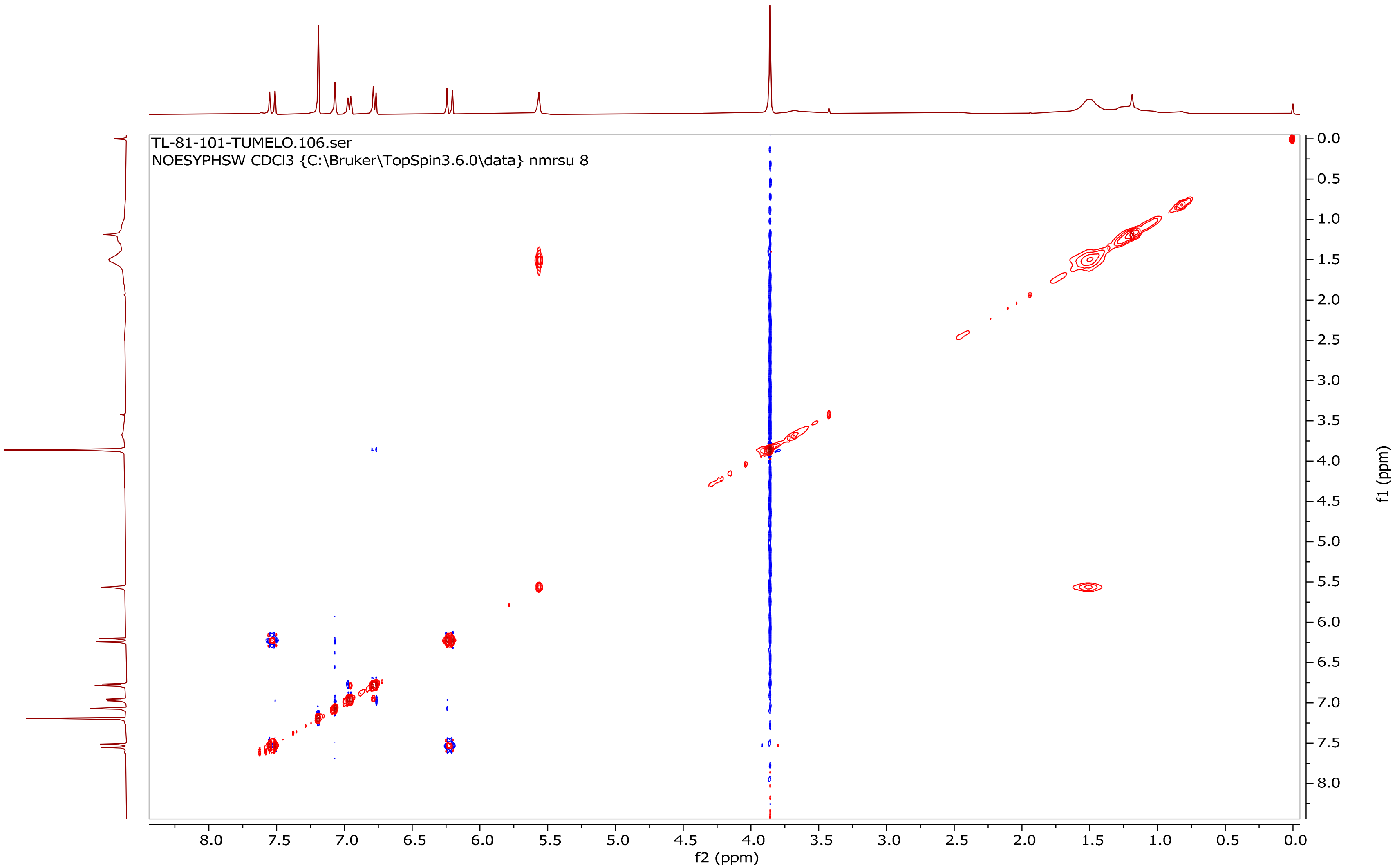

Figure SD.36: Nuclear Overhauser Effect Spectroscopy (NOESY) spectrum of Ferulic Acid

5. **Spectral data of Compound E**

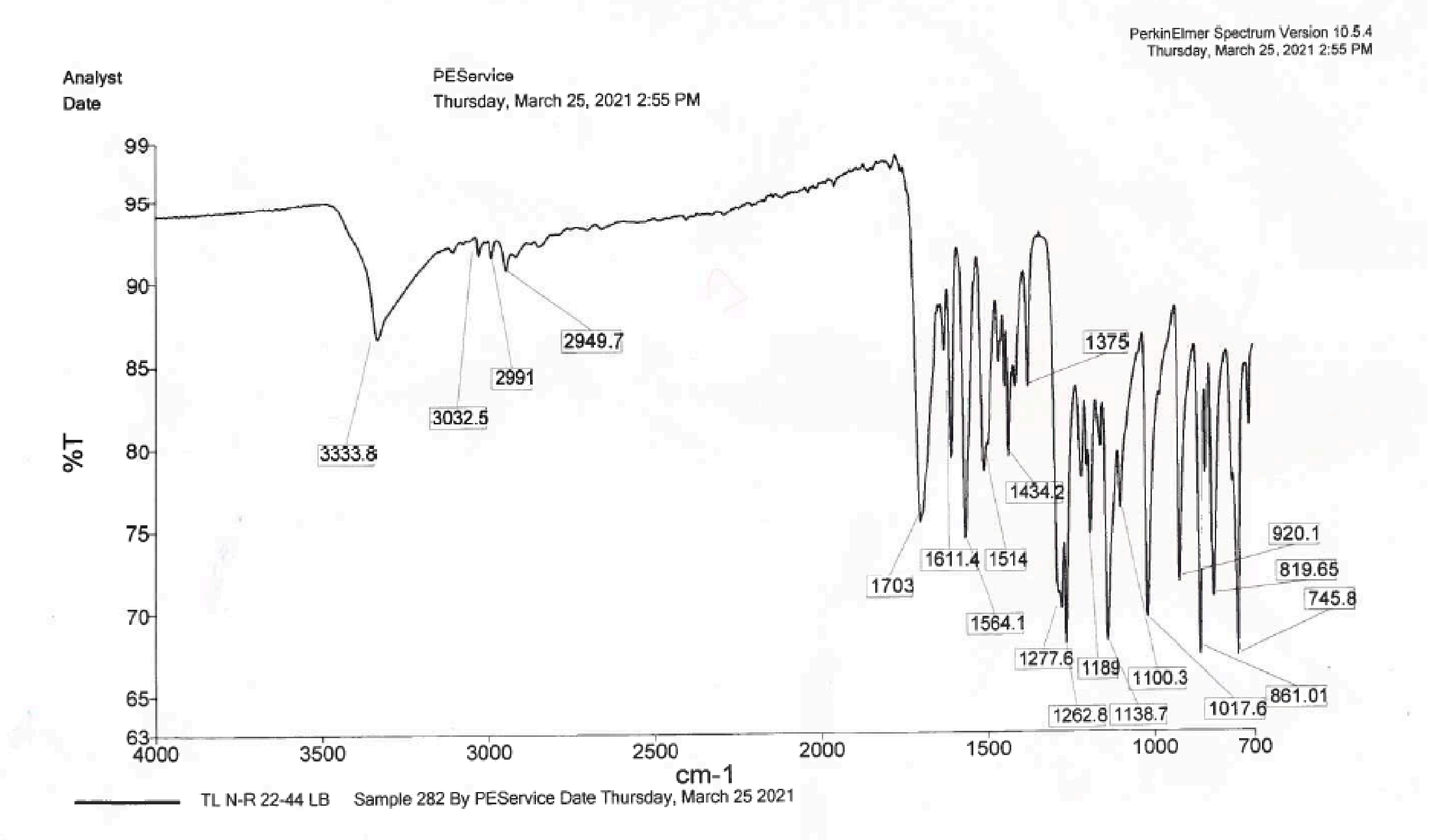

Figure SE.37: Fourier-Transform Infrared Spectroscopy (FTIR) spectrum of Scopoletin [7-hydroxy-6-methoxychromen-2-one]

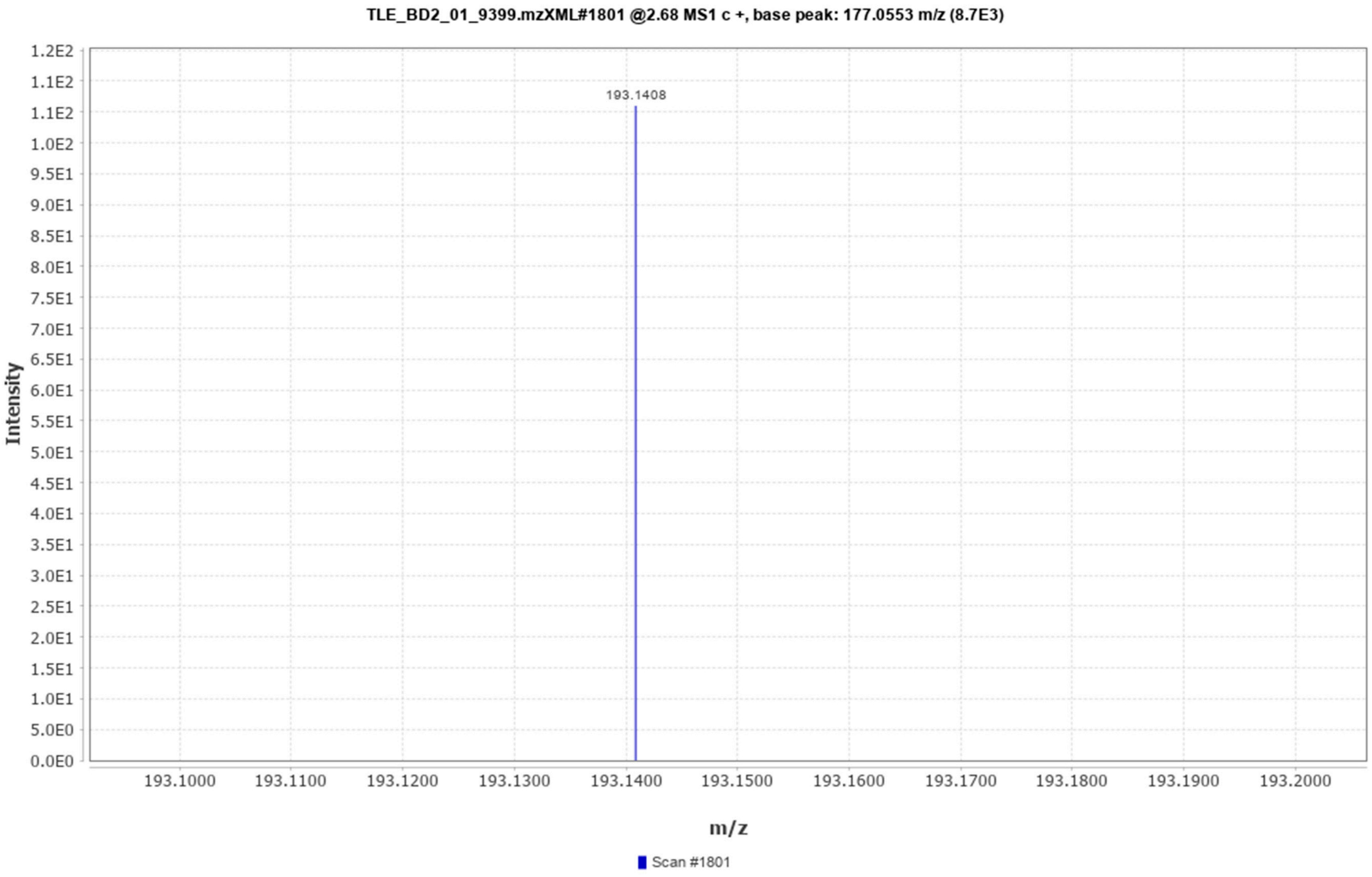

Figure SE.38: High-Resolution Electrospray Ionization Mass spectrum (HR-ESI-MS) of Scopoletin $[M+H]^+$ m/z = 193.1408

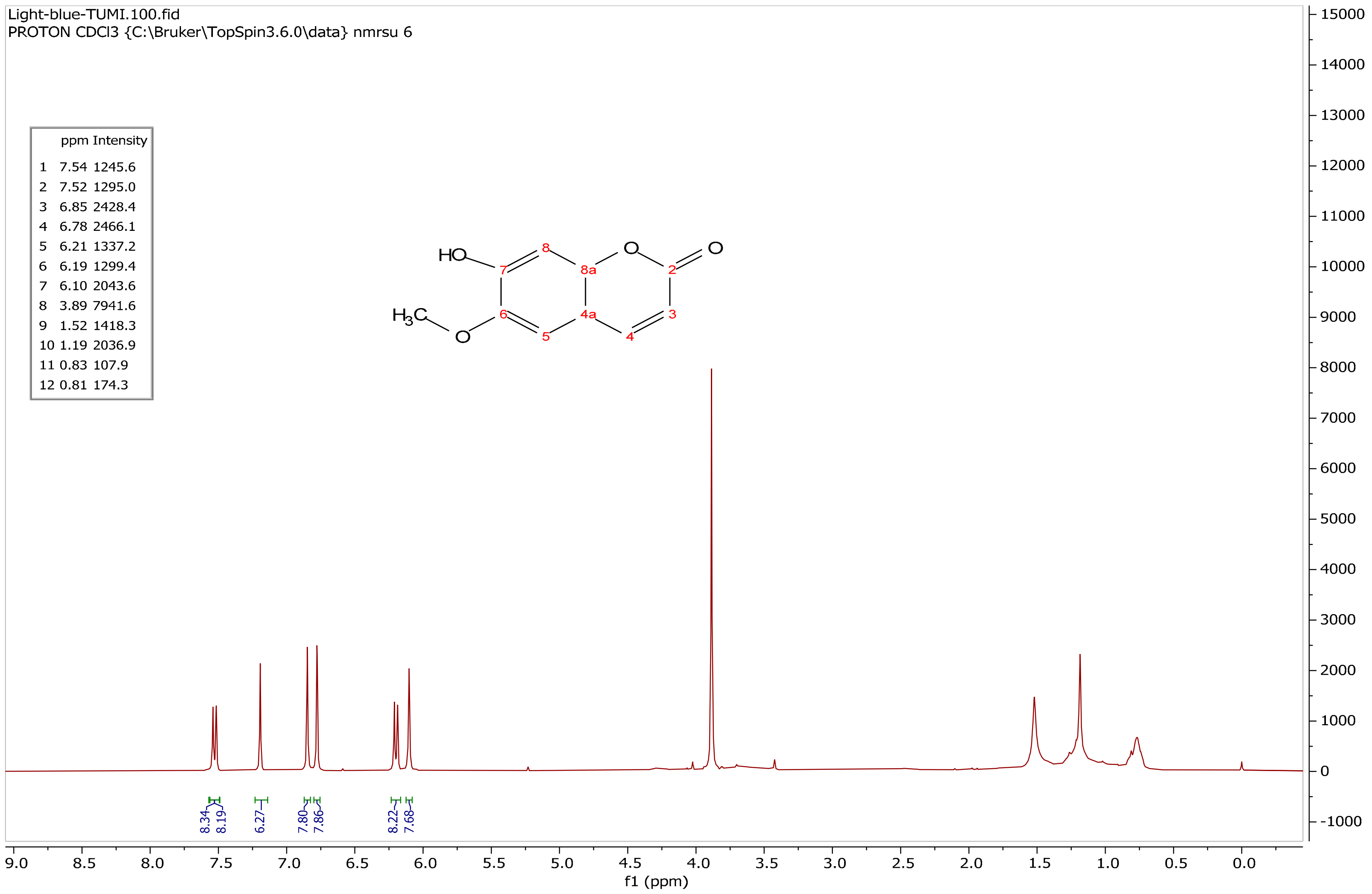

Figure SE.39: Proton Nuclear Magnetic Resonance (^{1}H NMR) spectrum of Scopoletin ($CDCl_3$, 400 MHz)

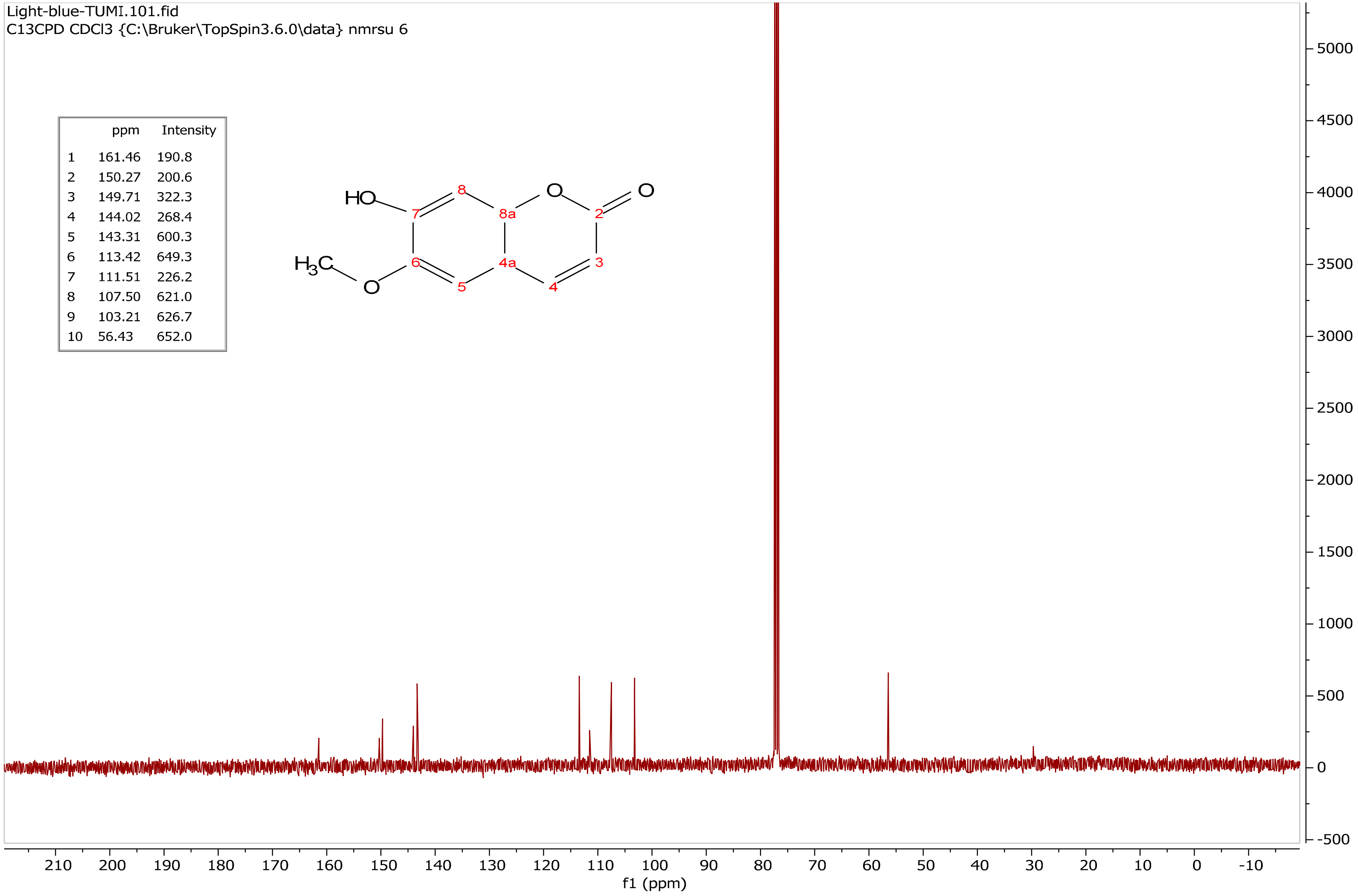

Figure SE.40: Carbon-13 Nuclear Magnetic Resonance (^{13}C NMR) spectrum of Scopoletin ($CDCl_3$, 100 MHz)

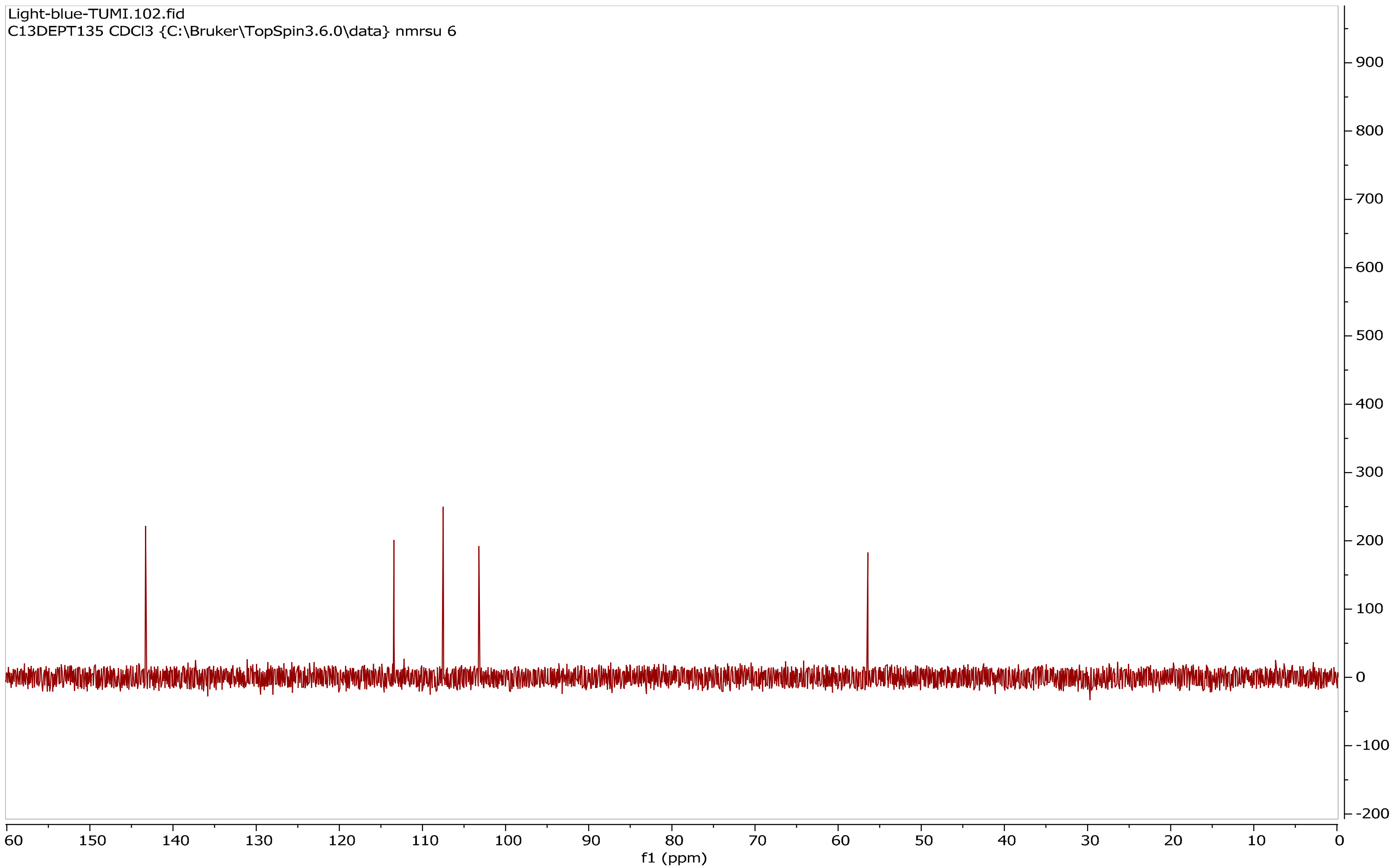

Figure SE.41: Distortionless Enhancement by Polarization Transfer (DEPT) NMR spectra Scopoletin ($CDCl_3$, 100 MHz)

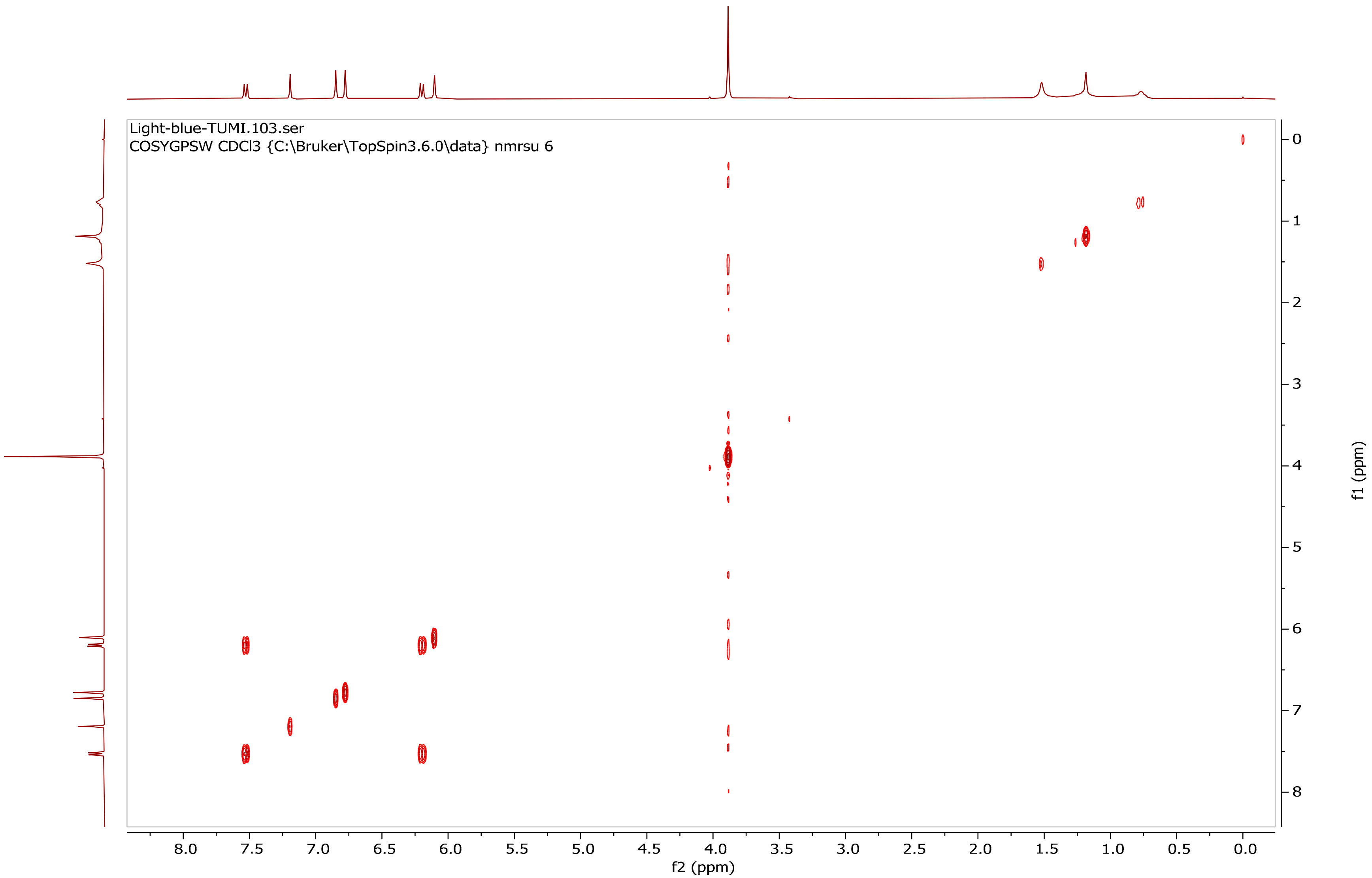

Figure SE.42: Gradient Correlated (gCOSY) spectrum of Scopoletin

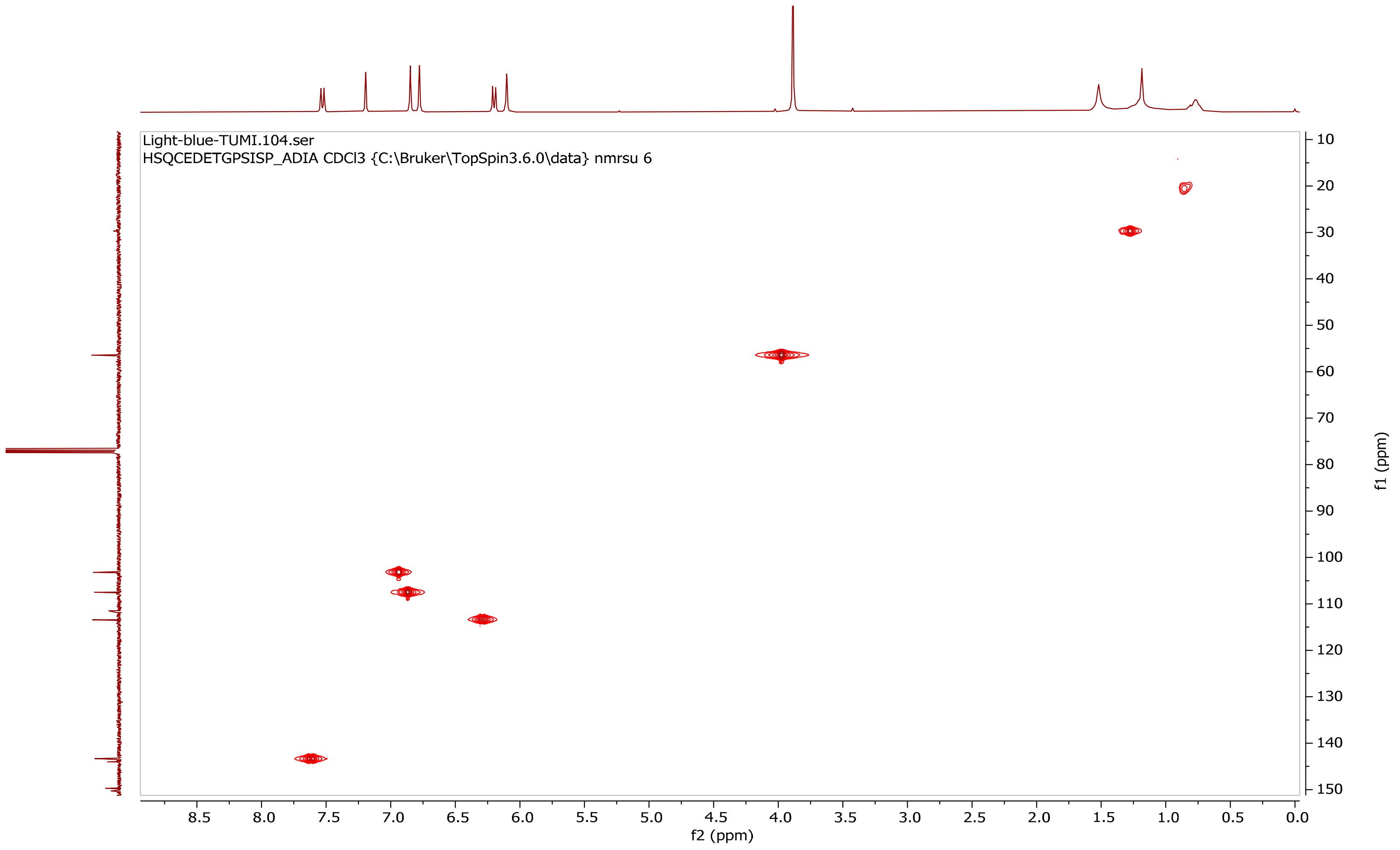

Figure SE.43: Gradient Heteronuclear Single Quantum Coherence (gHSQC) spectrum of Scopoletin

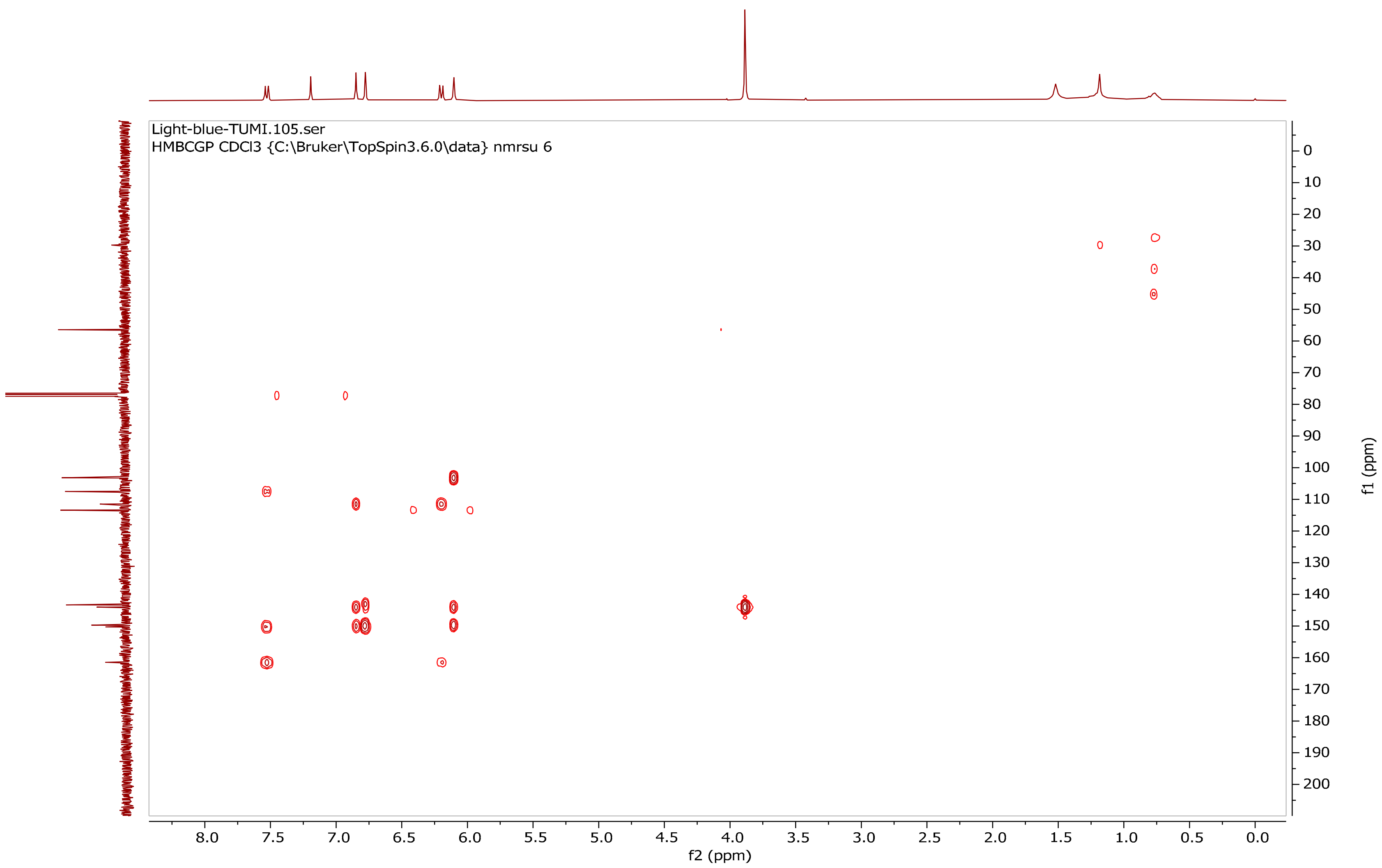

Figure SE.44: Gradient Heteronuclear Multiple Bond Quantum Coherence (gHMBC) spectrum of Scopoletin

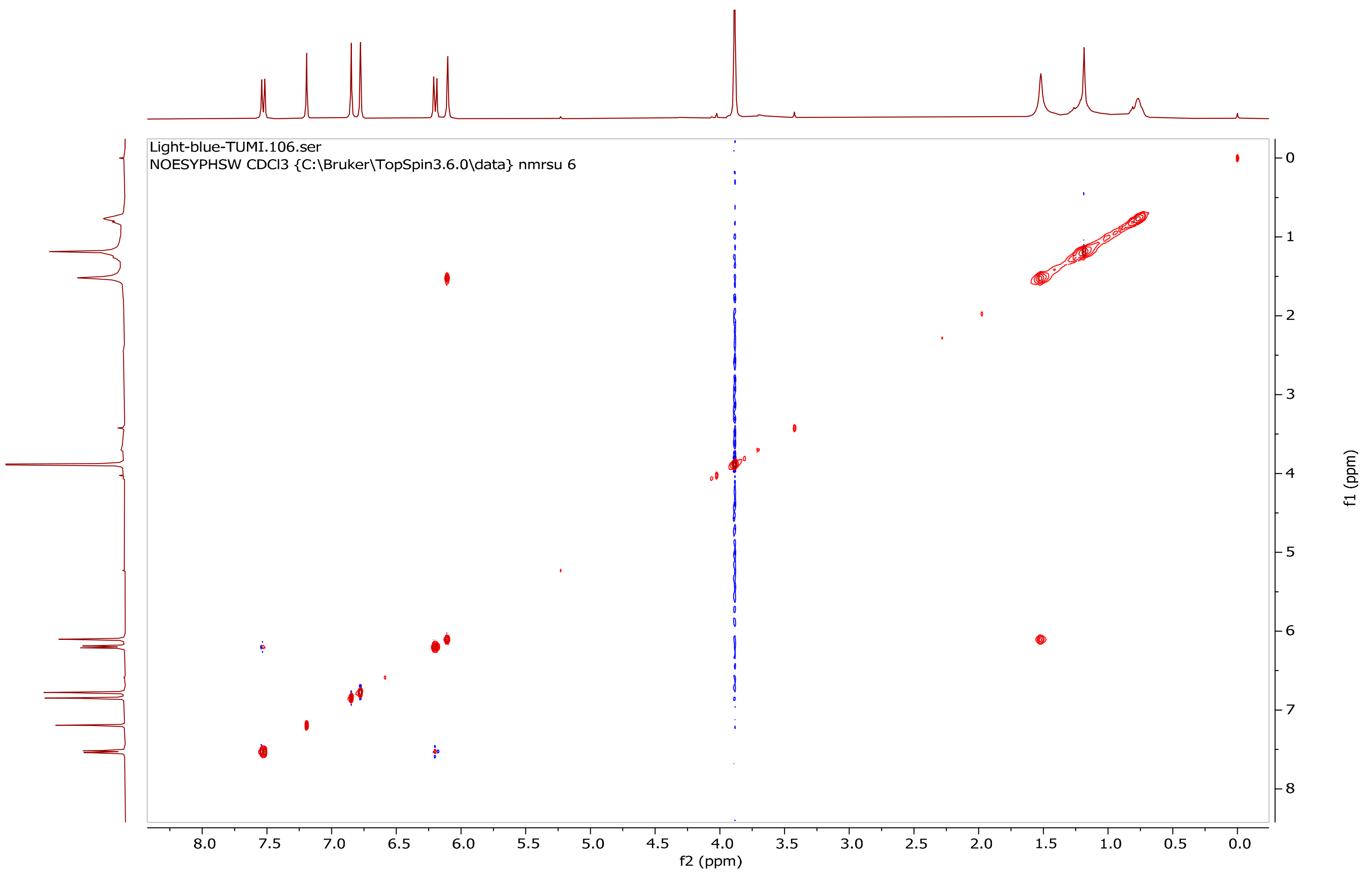

Figure SE.45: Nuclear Overhauser Effect Spectroscopy (NOESY) spectrum of Scopoletin

6. **Spectral data of Compound F**

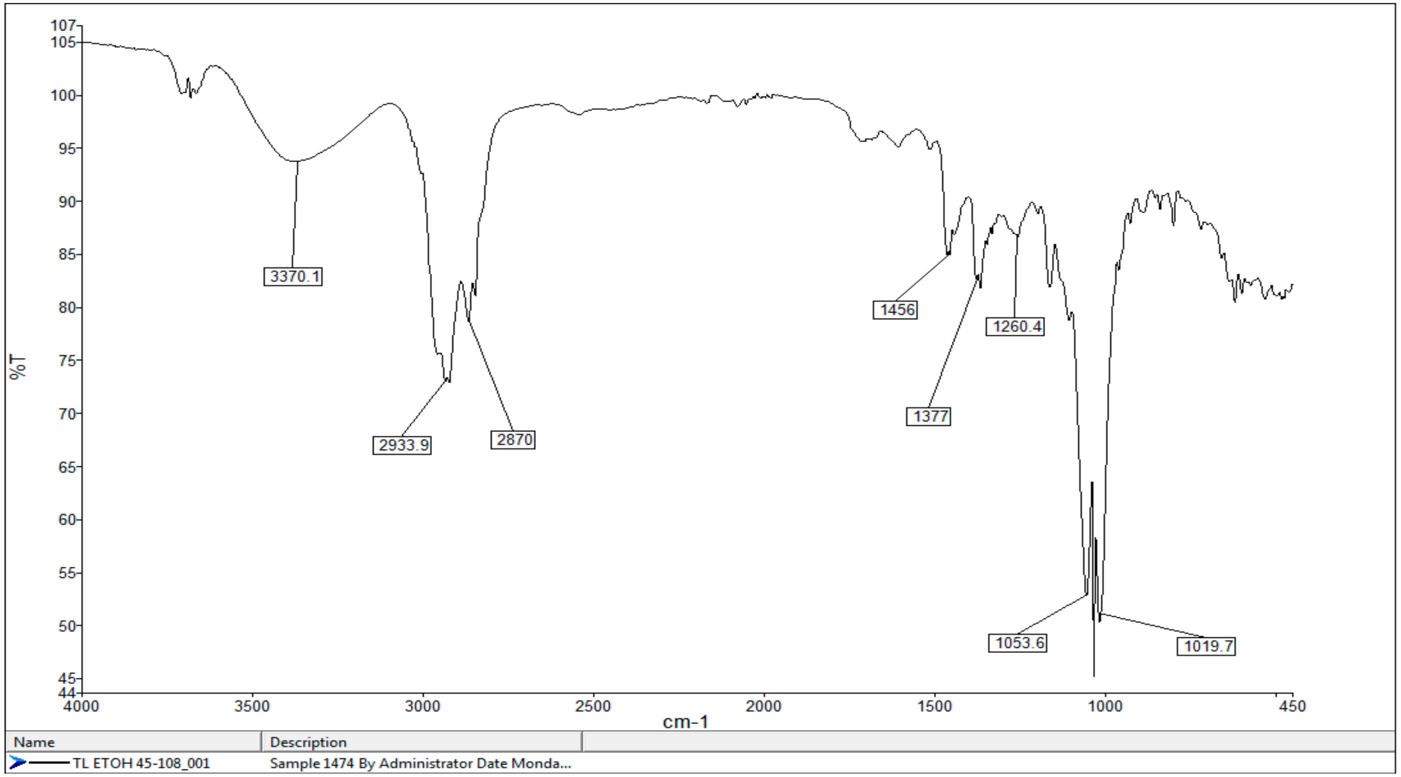

Figure SF.46: Fourier-Transform Infrared Spectroscopy (FTIR) spectrum of Sitosterol glucopyranoside

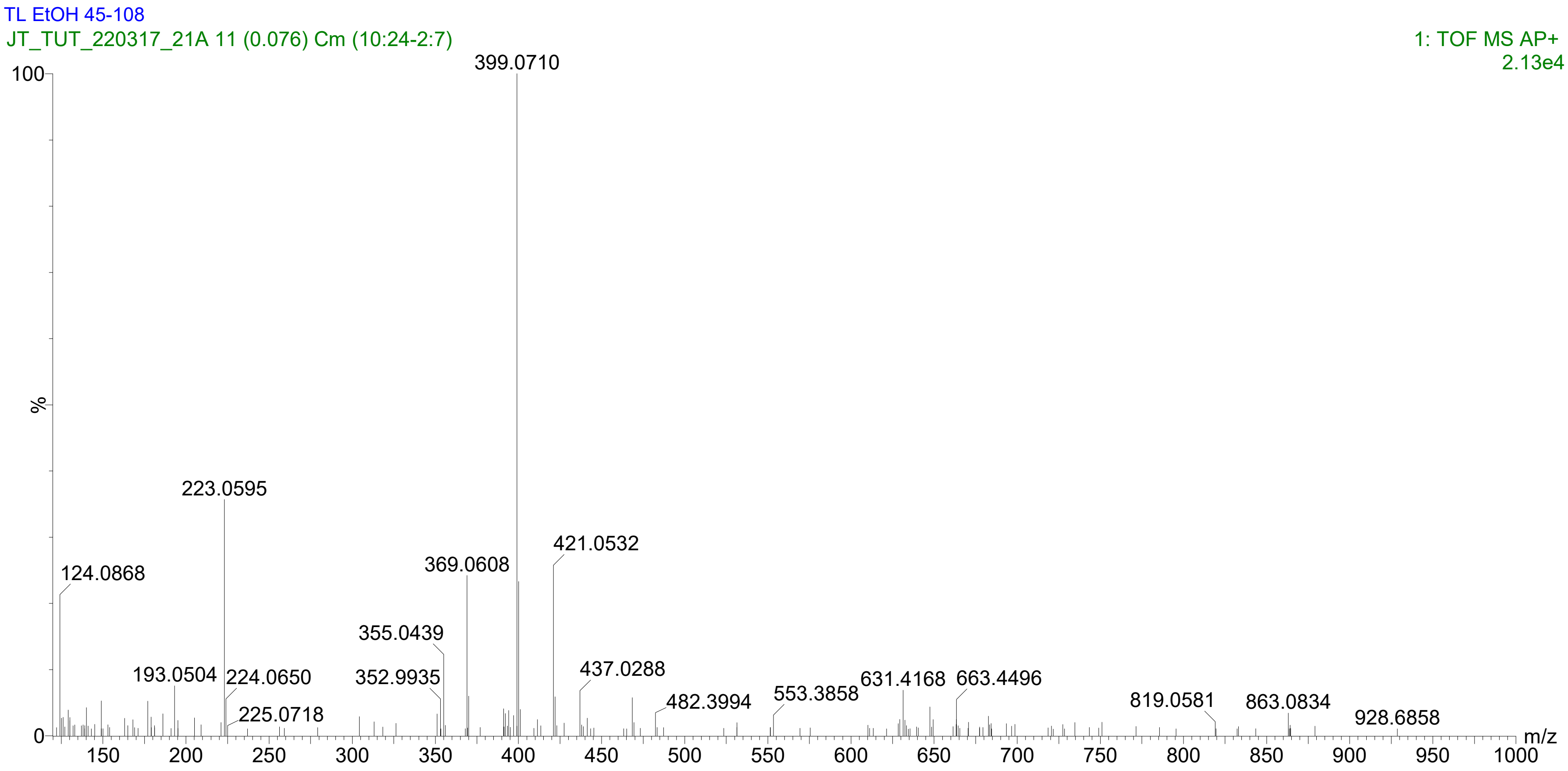

Figure SF.47: High-Resolution Electrospray Ionization Mass spectrum (HR-ESI-MS) of Sitosterol glucopyranoside [M + H–H_2O $C_6H_{12}O_6$] $^+$. m/z = 399.0710

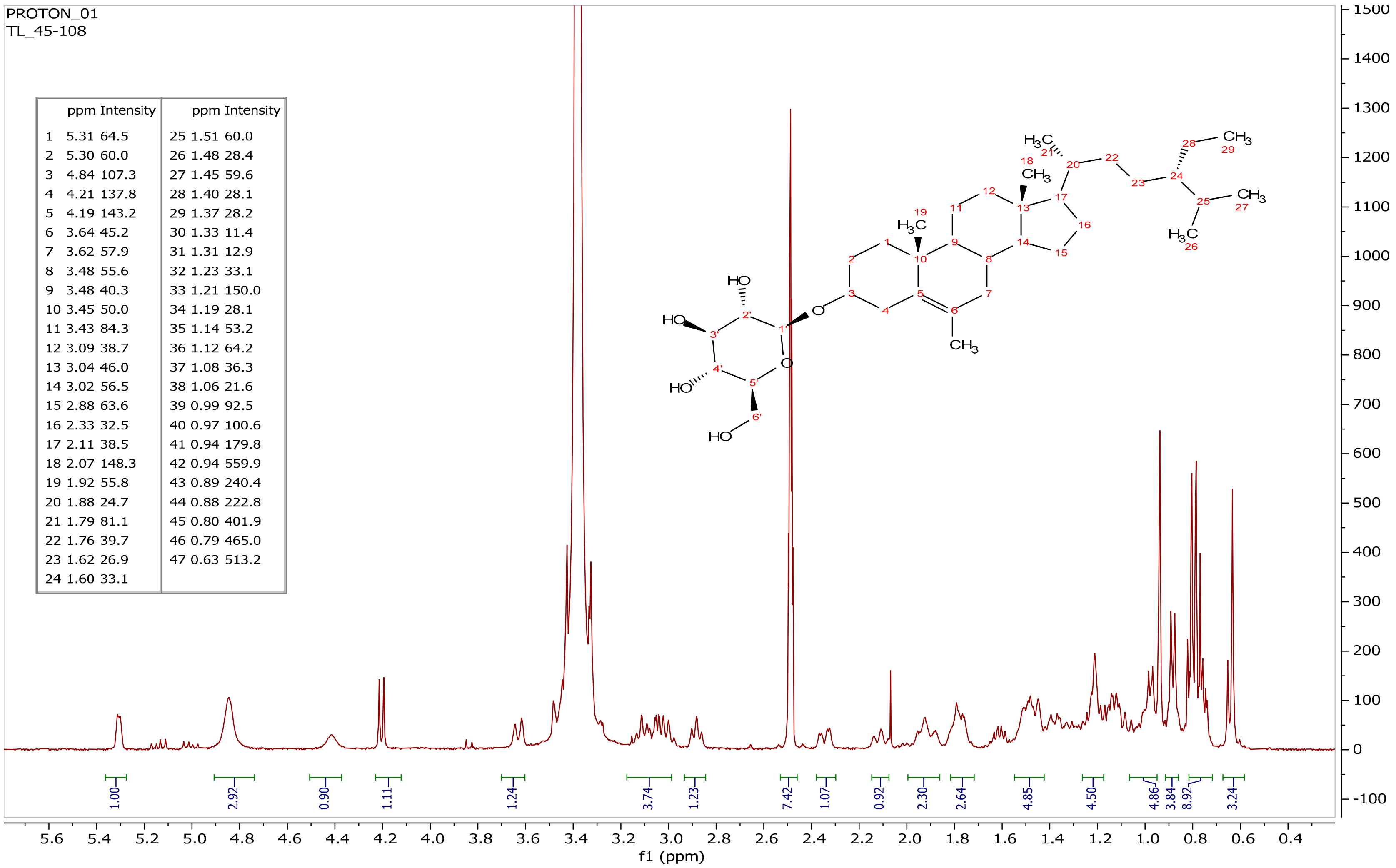

	ppm	Intensity		ppm	Intensity
1	5.31	64.5	25	1.51	60.0
2	5.30	60.0	26	1.48	28.4
3	4.84	107.3	27	1.45	59.6
4	4.21	137.8	28	1.40	28.1
5	4.19	143.2	29	1.37	28.2
6	3.64	45.2	30	1.33	11.4
7	3.62	57.9	31	1.31	12.9
8	3.48	55.6	32	1.23	33.1
9	3.48	40.3	33	1.21	150.0
10	3.45	50.0	34	1.19	28.1
11	3.43	84.3	35	1.14	53.2
12	3.09	38.7	36	1.12	64.2
13	3.04	46.0	37	1.08	36.3
14	3.02	56.5	38	1.06	21.6
15	2.88	63.6	39	0.99	92.5
16	2.33	32.5	40	0.97	100.6
17	2.11	38.5	41	0.94	179.8
18	2.07	148.3	42	0.94	559.9
19	1.92	55.8	43	0.89	240.4
20	1.88	24.7	44	0.88	222.8
21	1.79	81.1	45	0.80	401.9
22	1.76	39.7	46	0.79	465.0
23	1.62	26.9	47	0.63	513.2
24	1.60	33.1			

Figure SF.48: Proton Nuclear Magnetic Resonance (^{1}H NMR) spectrum of Sitosterol glucopyranoside (DMSO, 400 MHz)

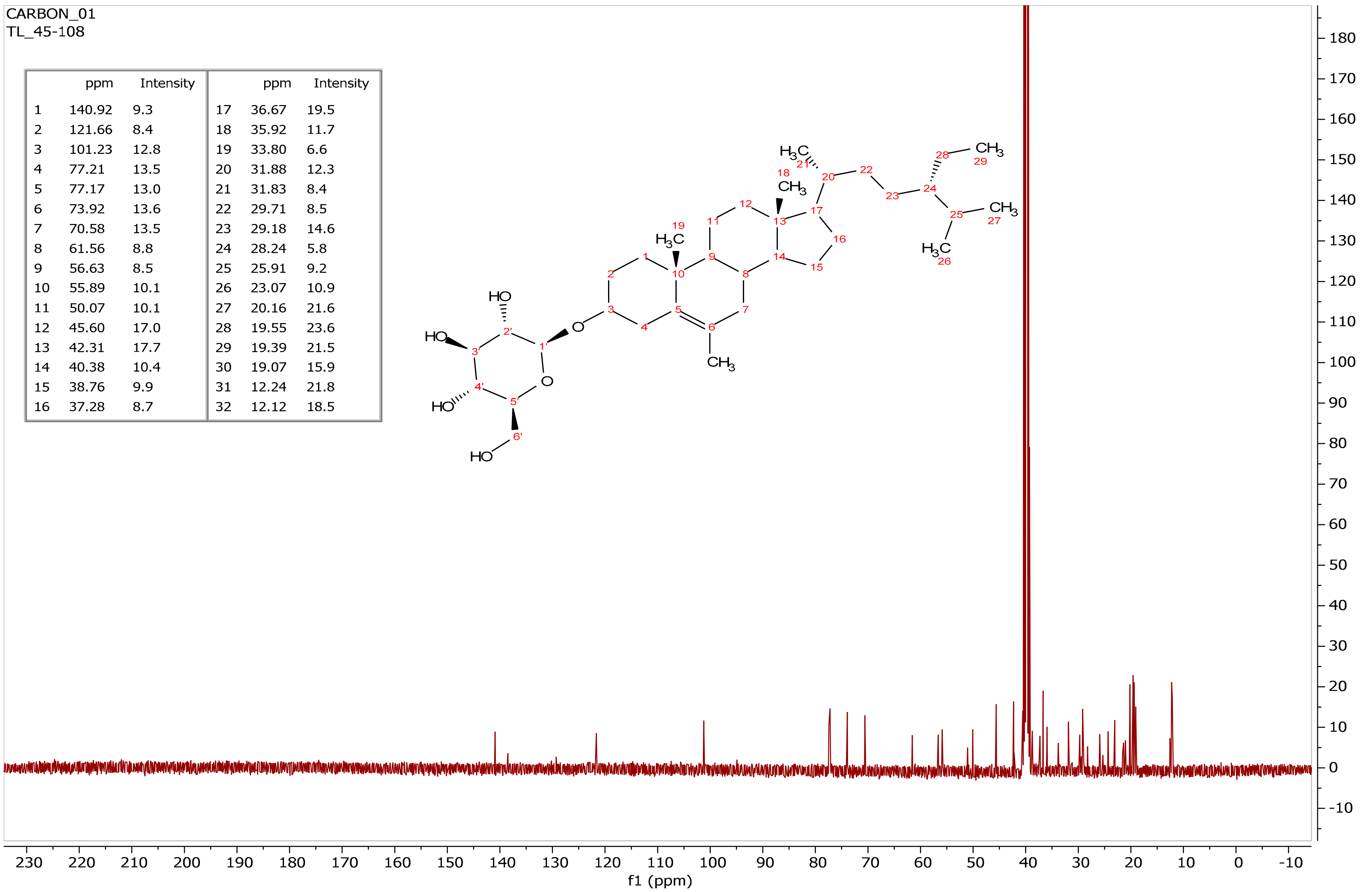

	ppm	Intensity		ppm	Intensity
1	140.92	9.3	17	36.67	19.5
2	121.66	8.4	18	35.92	11.7
3	101.23	12.8	19	33.80	6.6
4	77.21	13.5	20	31.88	12.3
5	77.17	13.0	21	31.83	8.4
6	73.92	13.6	22	29.71	8.5
7	70.58	13.5	23	29.18	14.6
8	61.56	8.8	24	28.24	5.8
9	56.63	8.5	25	25.91	9.2
10	55.89	10.1	26	23.07	10.9
11	50.07	10.1	27	20.16	21.6
12	45.60	17.0	28	19.55	23.6
13	42.31	17.7	29	19.39	21.5
14	40.38	10.4	30	19.07	15.9
15	38.76	9.9	31	12.24	21.8
16	37.28	8.7	32	12.12	18.5

Figure SF.49: Carbon-13 Nuclear Magnetic Resonance (^{13}C NMR) spectrum of Sitosterol glucopyranoside (DMSO, 100 MHz)

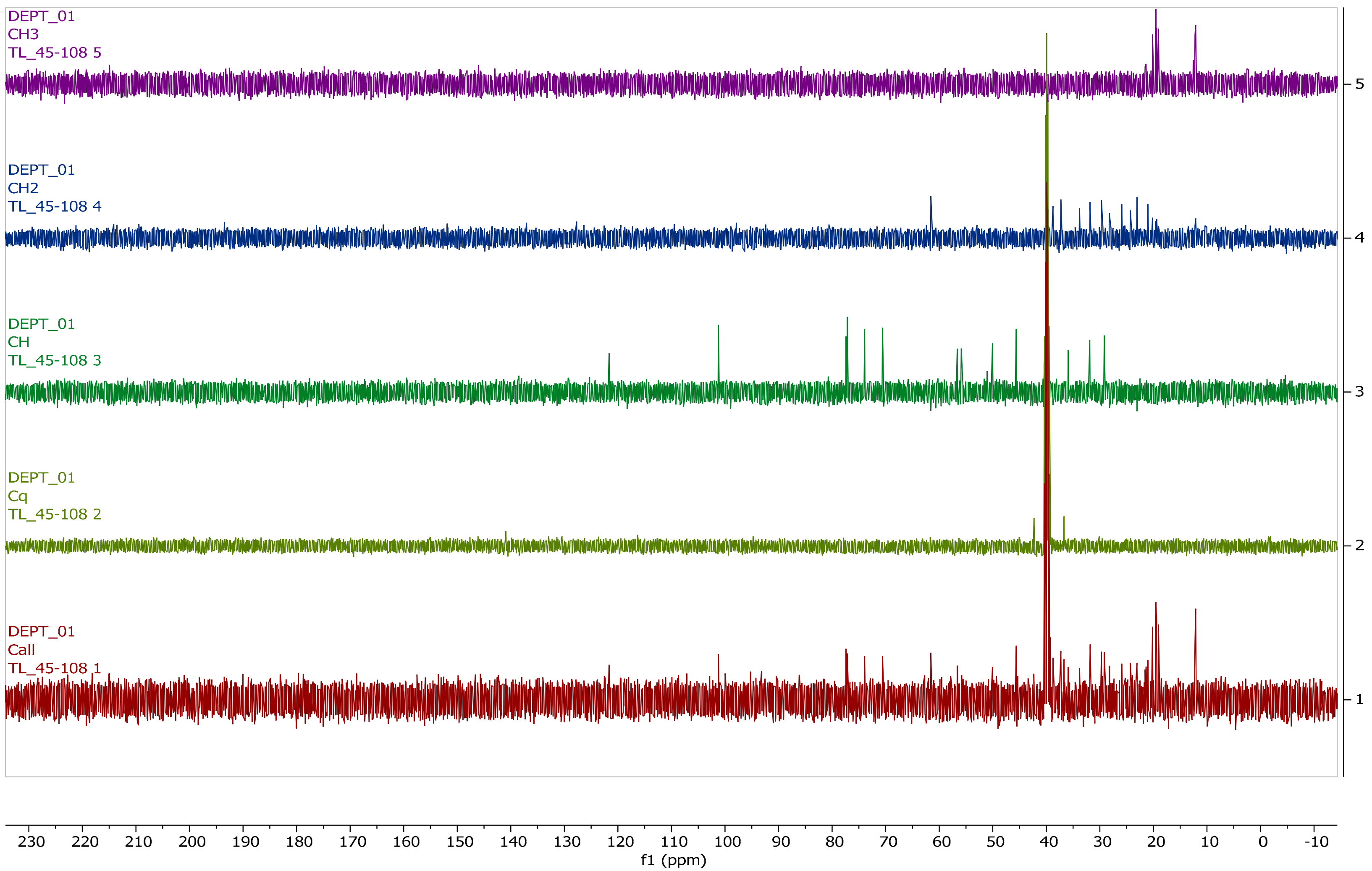

Figure SF.50: Distortionless Enhancement by Polarization Transfer (DEPT) NMR spectra Sitosterol glucopyranoside (DMSO, 100 MHz

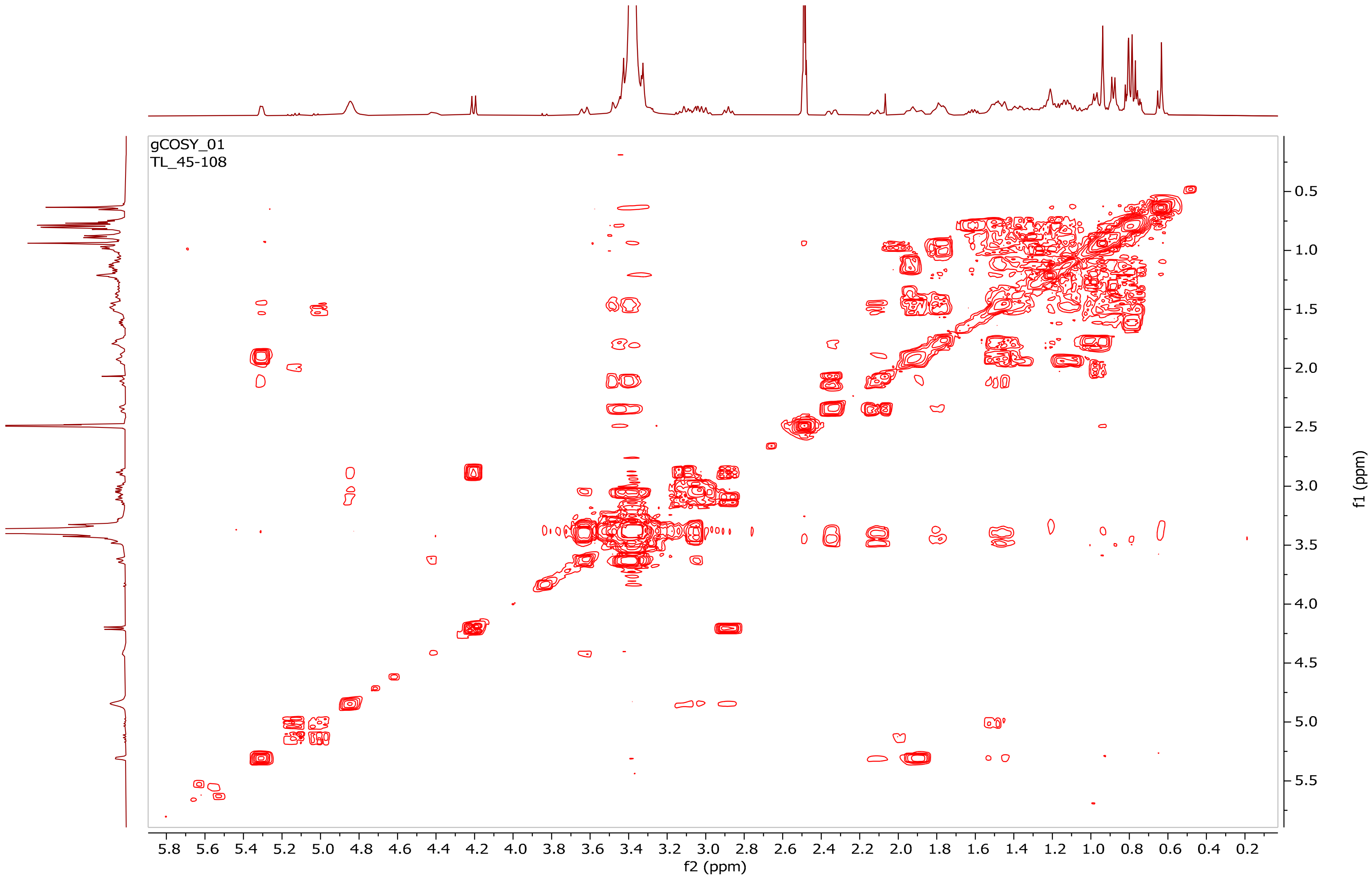

Figure SF.51: Gradient Correlated (gCOSY) spectrum of Sitosterol glucopyranoside

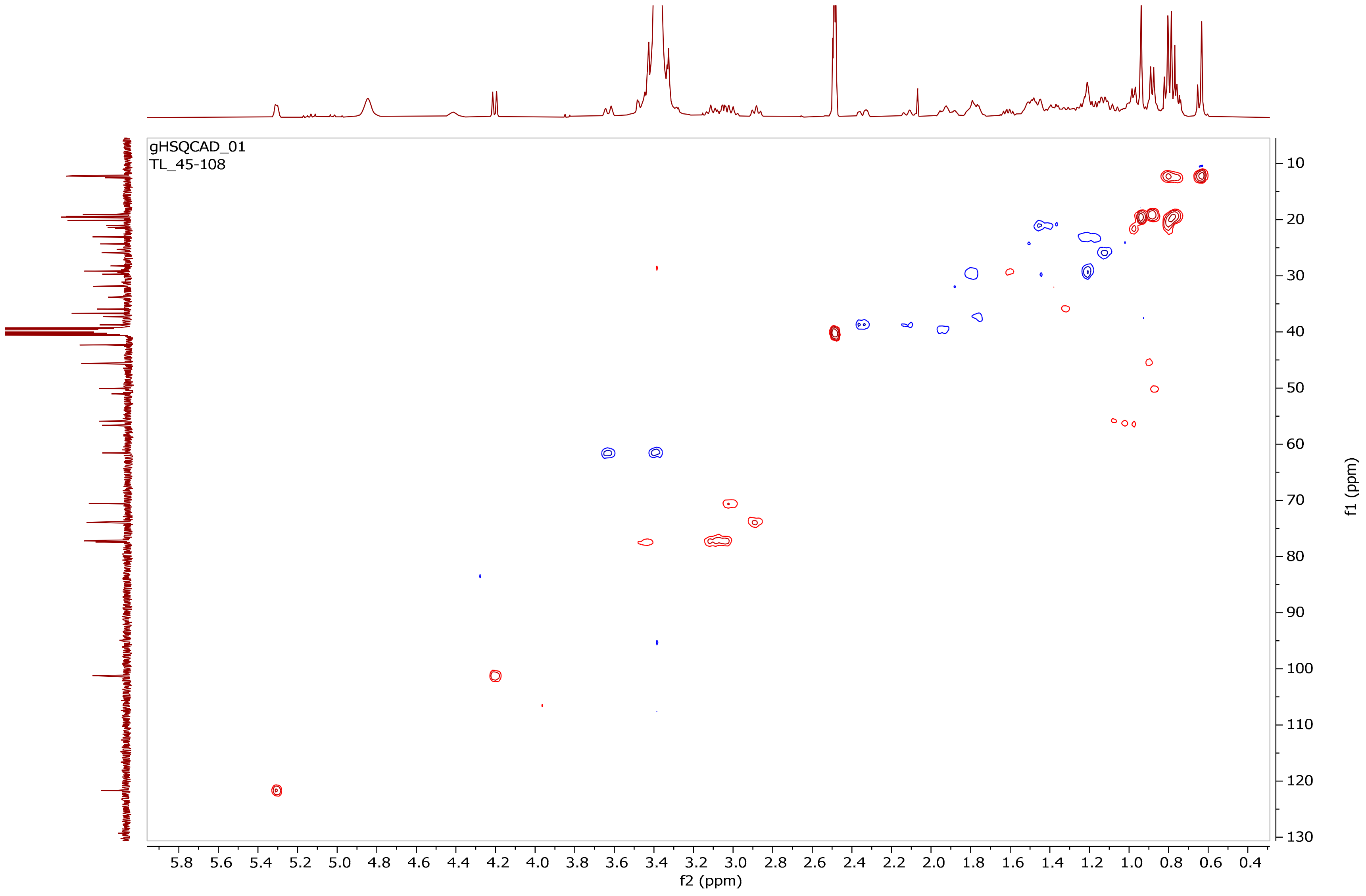

Figure SF.52: Gradient Heteronuclear Single Quantum Coherence (gHSQC) spectrum of Sitosterol glucopyranoside

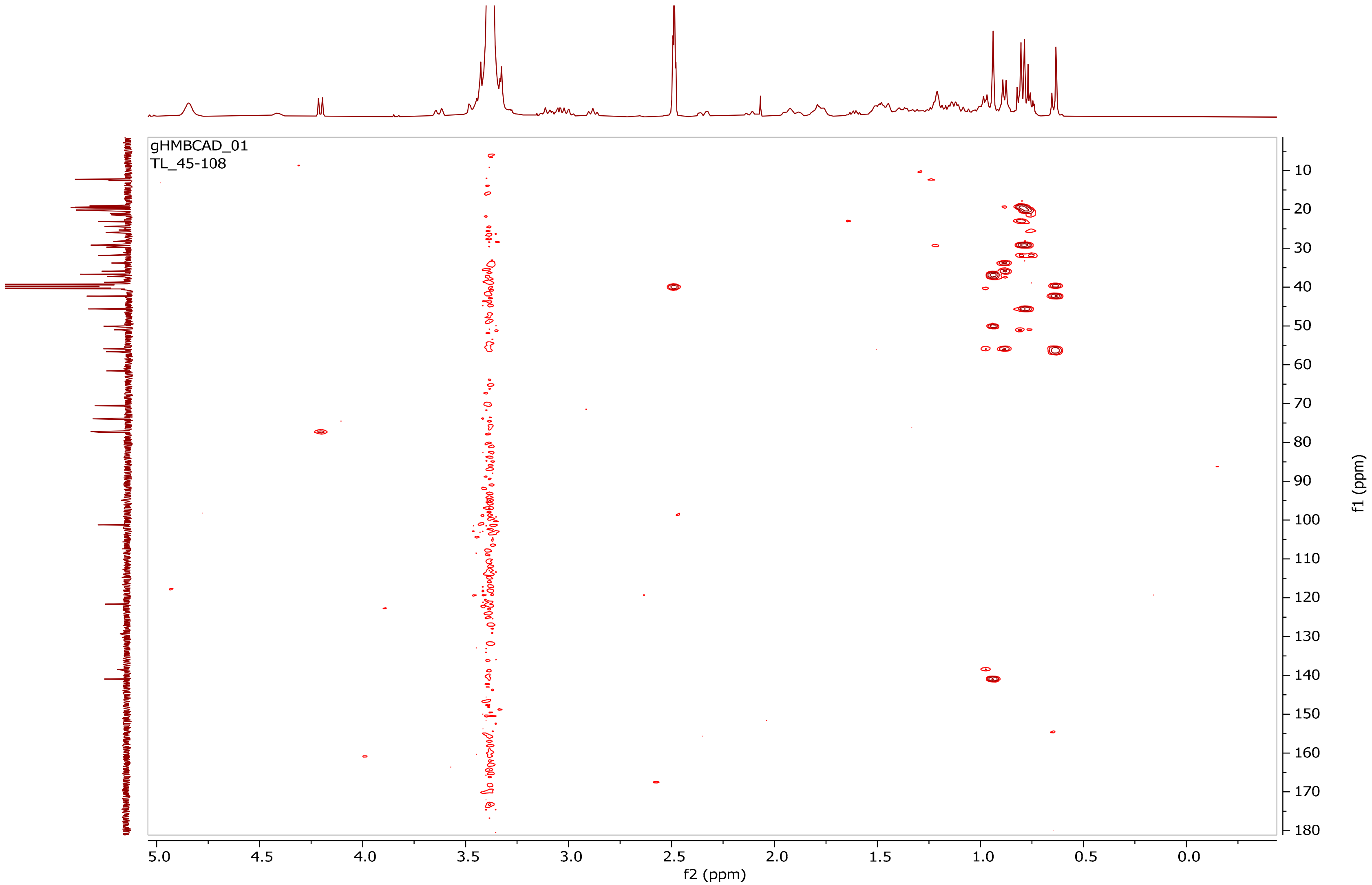

Figure SF.53: Gradient Heteronuclear Multiple Bond Quantum Coherence (gHMBC) spectrum of Sitosterol glucopyranoside

7. Spectral data of Compound G

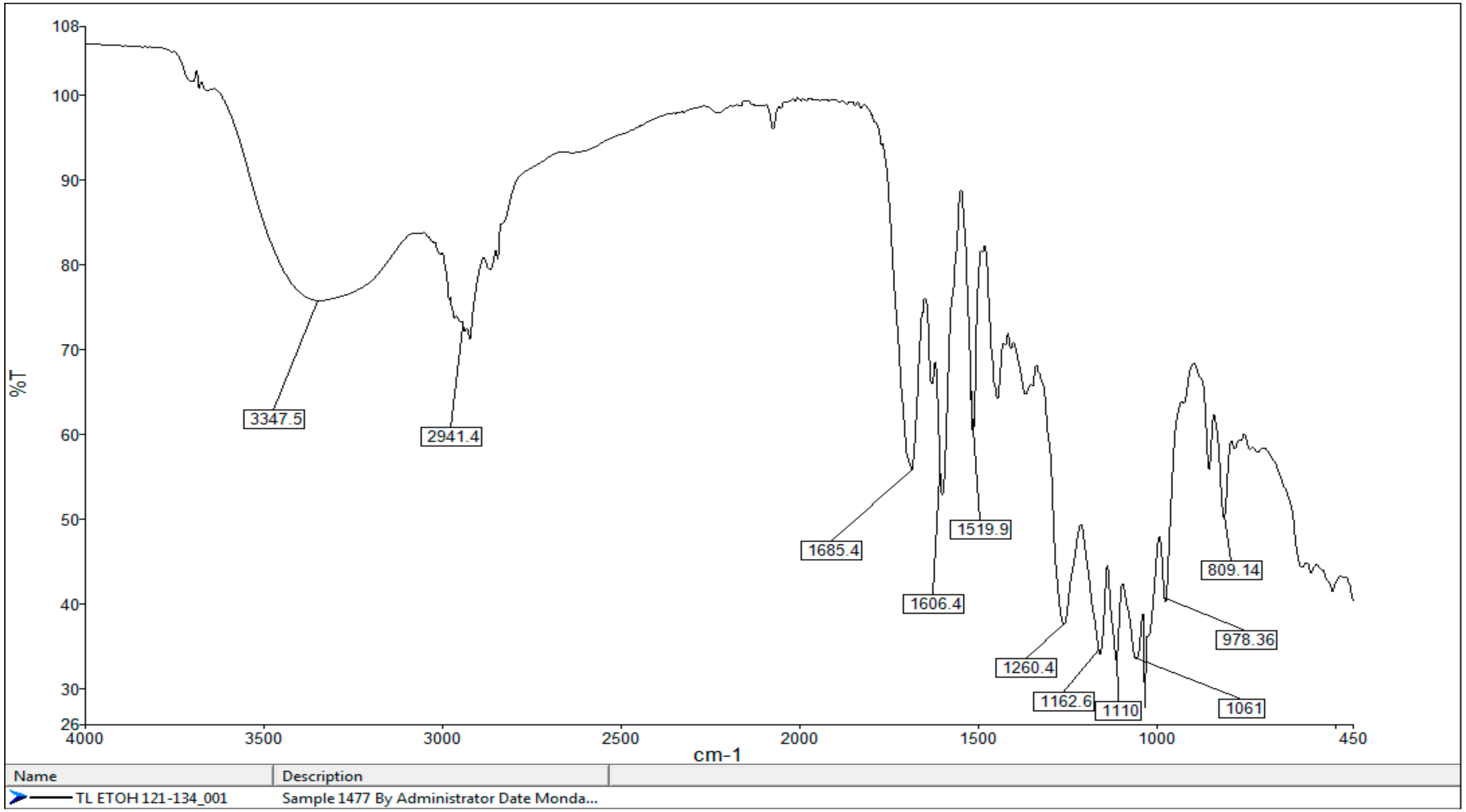

Figure SG.54: Fourier-Transform Infrared Spectroscopy (FTIR) spectrum of 1.5 Di-Feruloylquinicacid

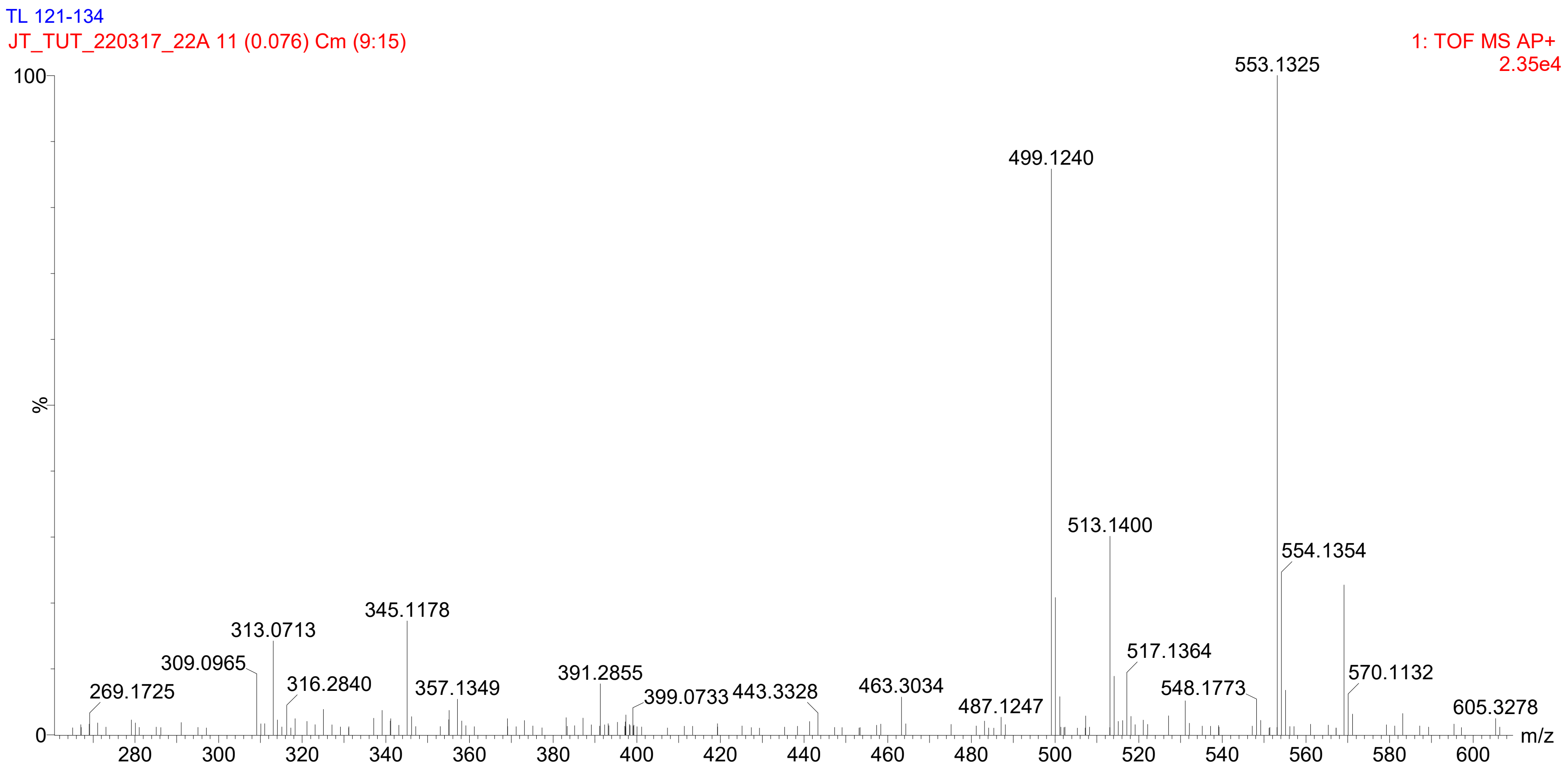

Figure SG.55: High-Resolution Electrospray Ionization Mass spectrum (HR-ESI-MS) of 1.5 Di-Feruloylquinicacid [M-Na+H_2O]$^-$ m/z = 553.1325

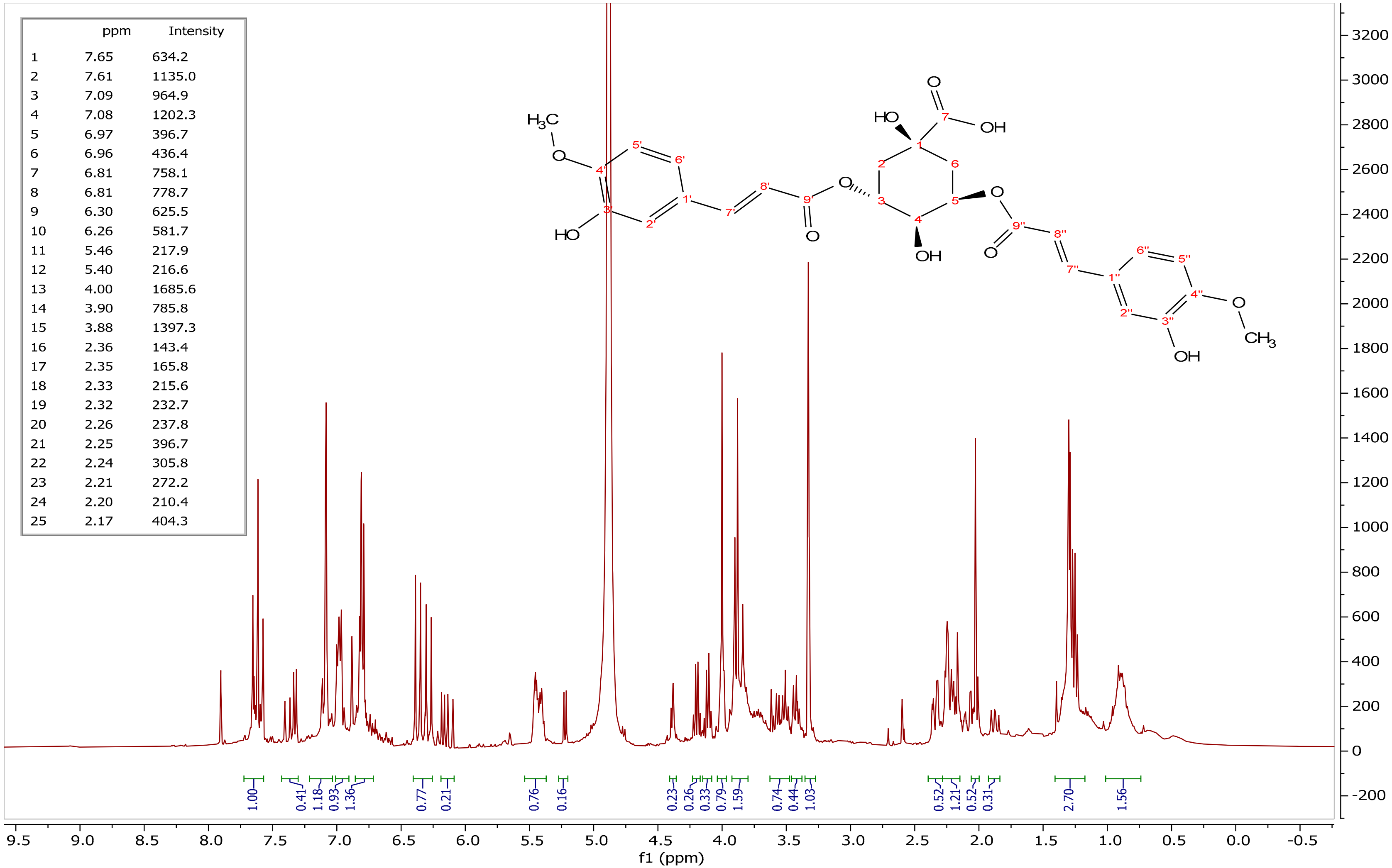

Figure SG.56: Proton Nuclear Magnetic Resonance (^{1}H NMR) spectrum of 1.5 Di-Feruloylquinicacid (MeOD, 400 MHz)

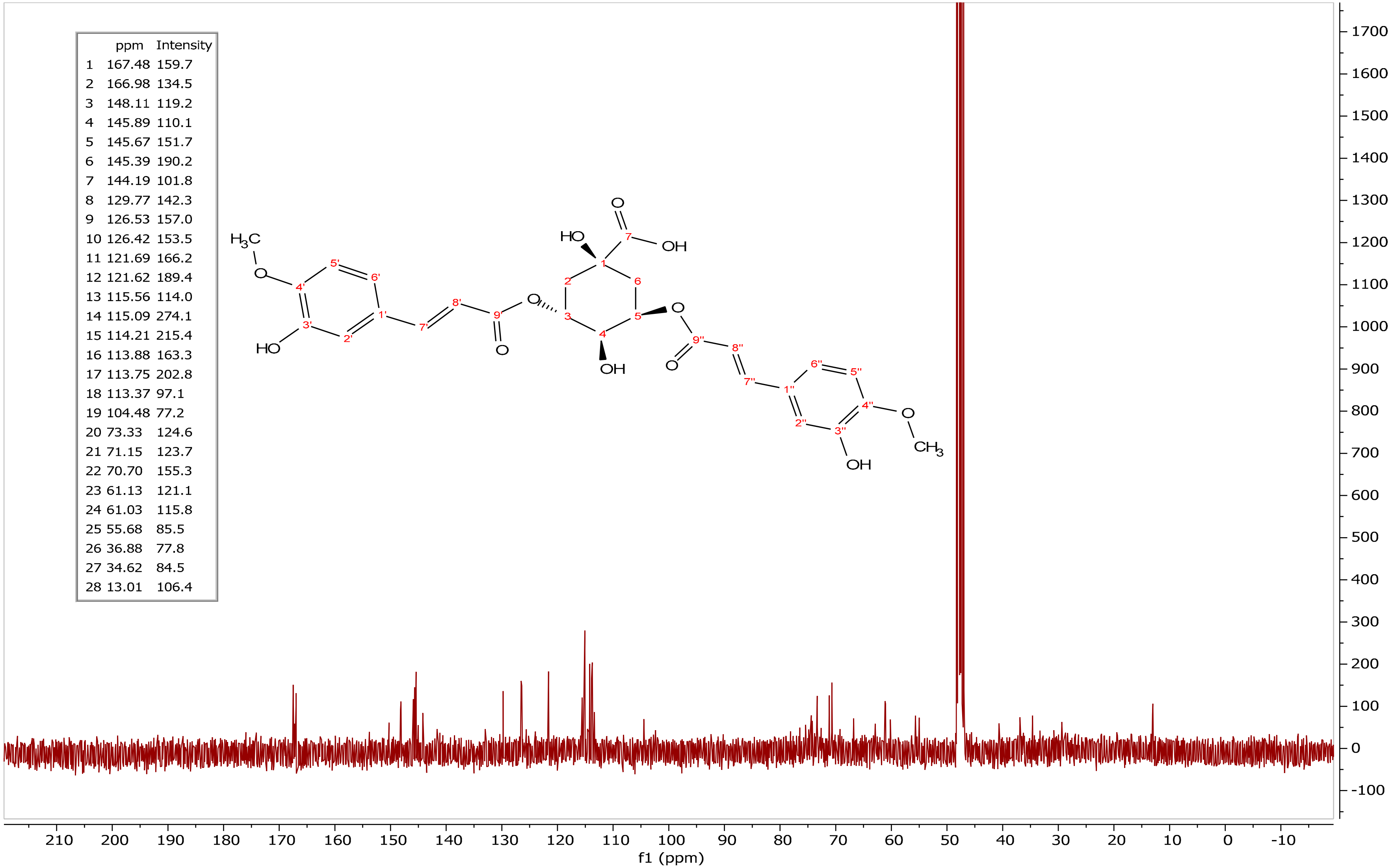

Figure SG.57: Carbon-13 Nuclear Magnetic Resonance (^{13}C NMR) spectrum of 1.5 Di-Feruloylquinicacid (MeOD, 100 MHz)

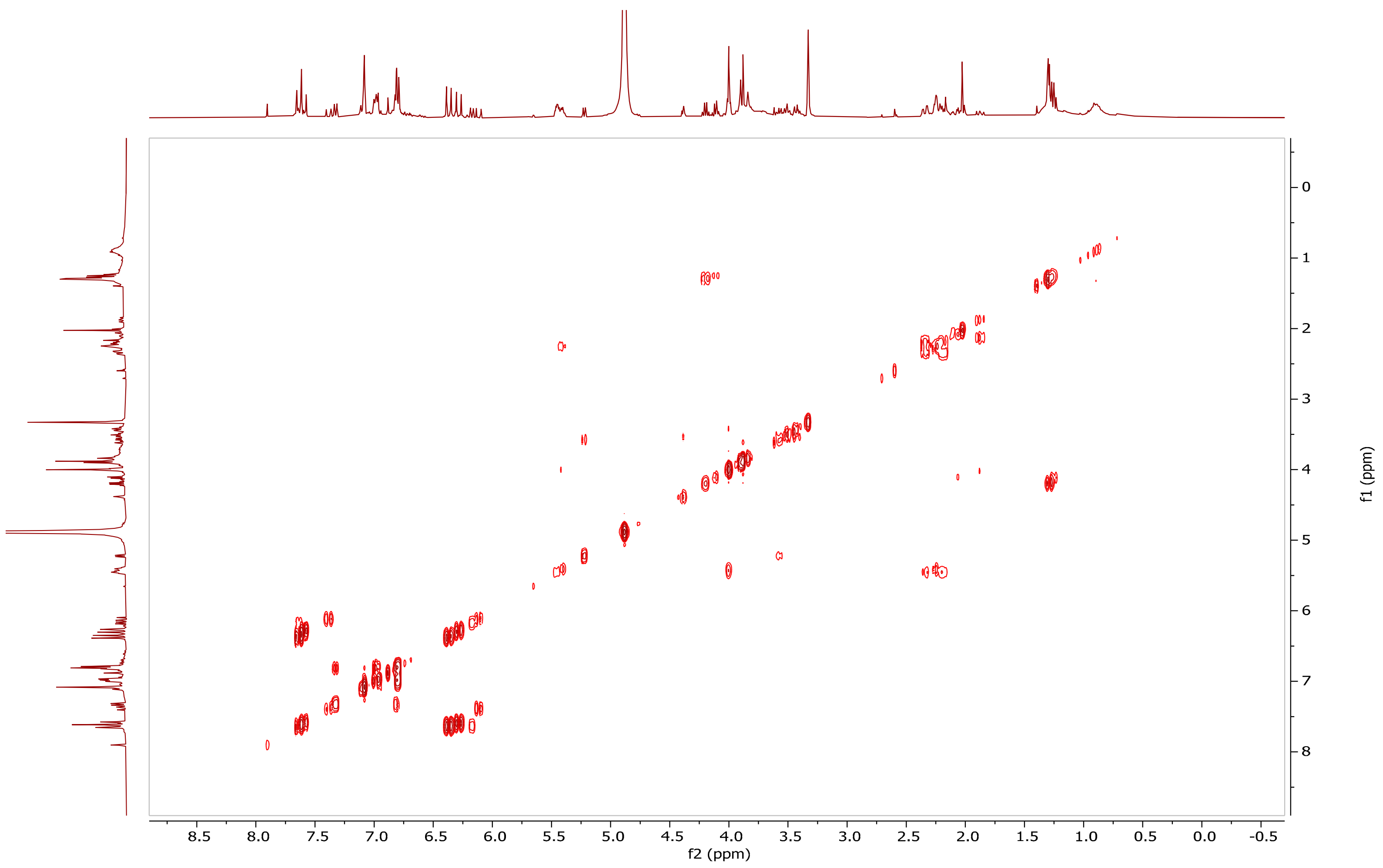

Figure SG.58: Gradient Correlated (gCOSY) spectrum of 1.5 Di-Feruloylquinicacid

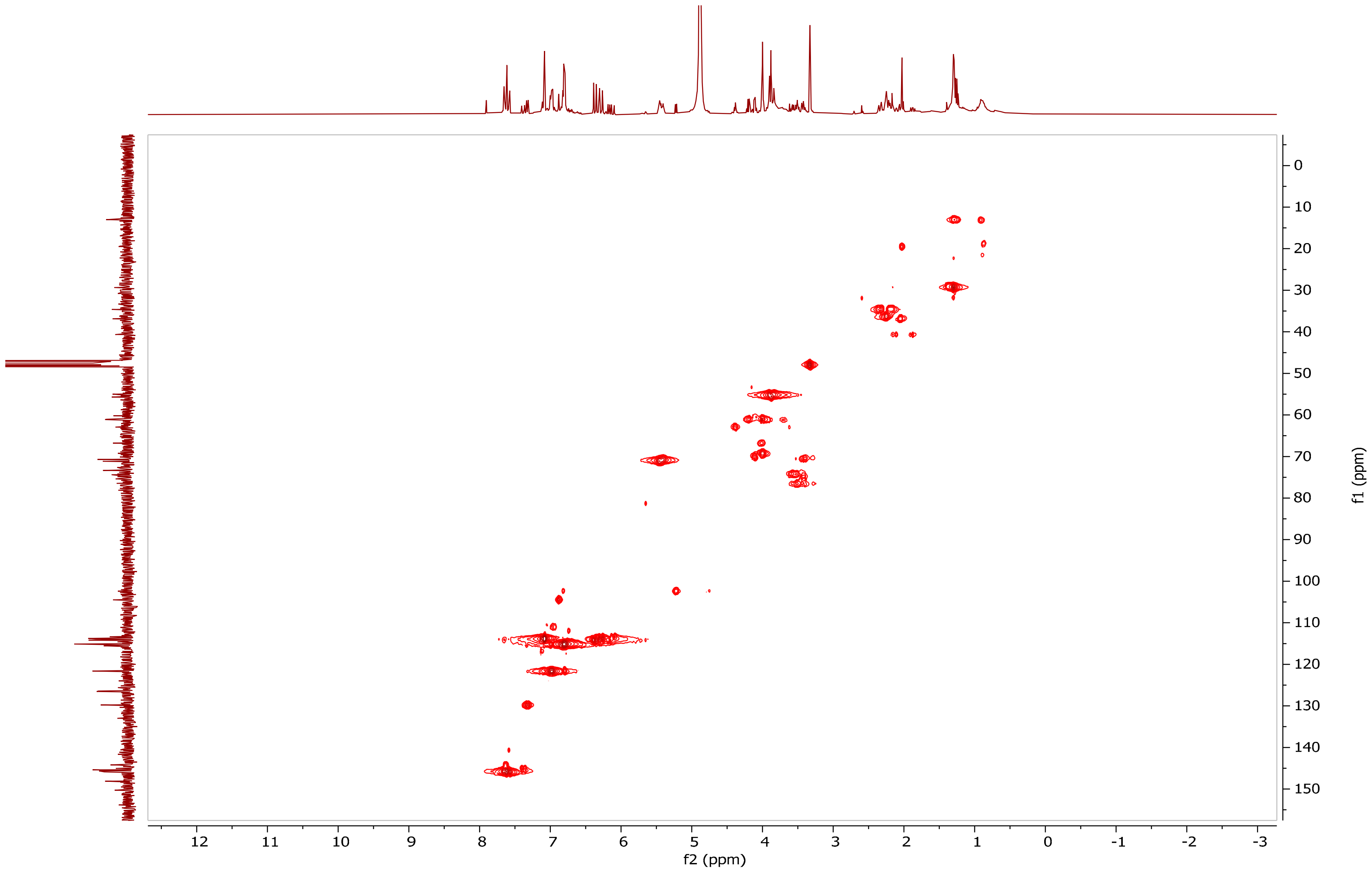

Figure SG.59: Gradient Heteronuclear Single Quantum Coherence (gHSQC) spectrum of 1.5 Di-Feruloylquinicacid

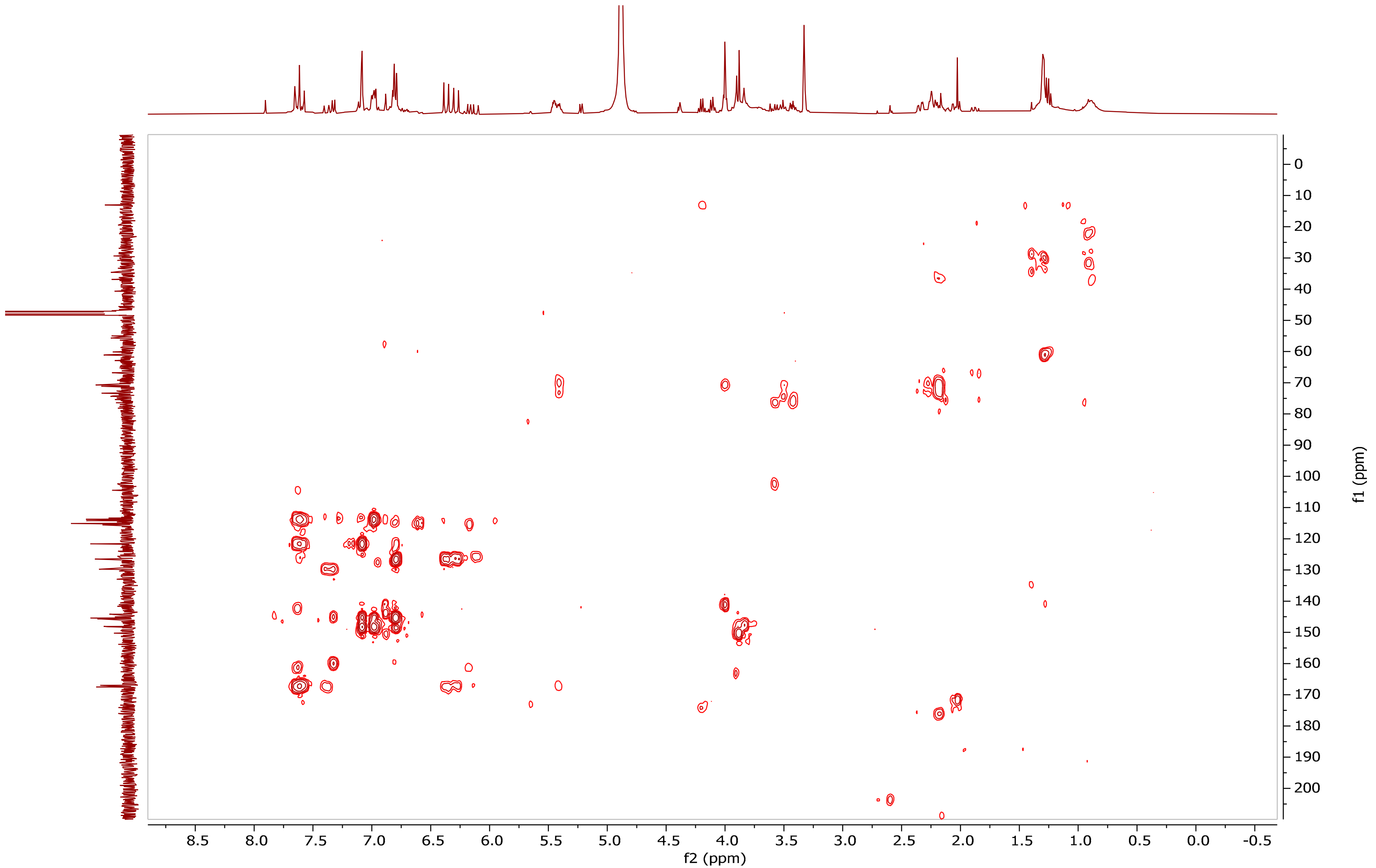

Figure SG.60: Gradient Heteronuclear Multiple Bond Quantum Coherence (gHMBC) spectrum of 1.5 Di-Feruloylquinicacid

8. Spectral data of Compound H

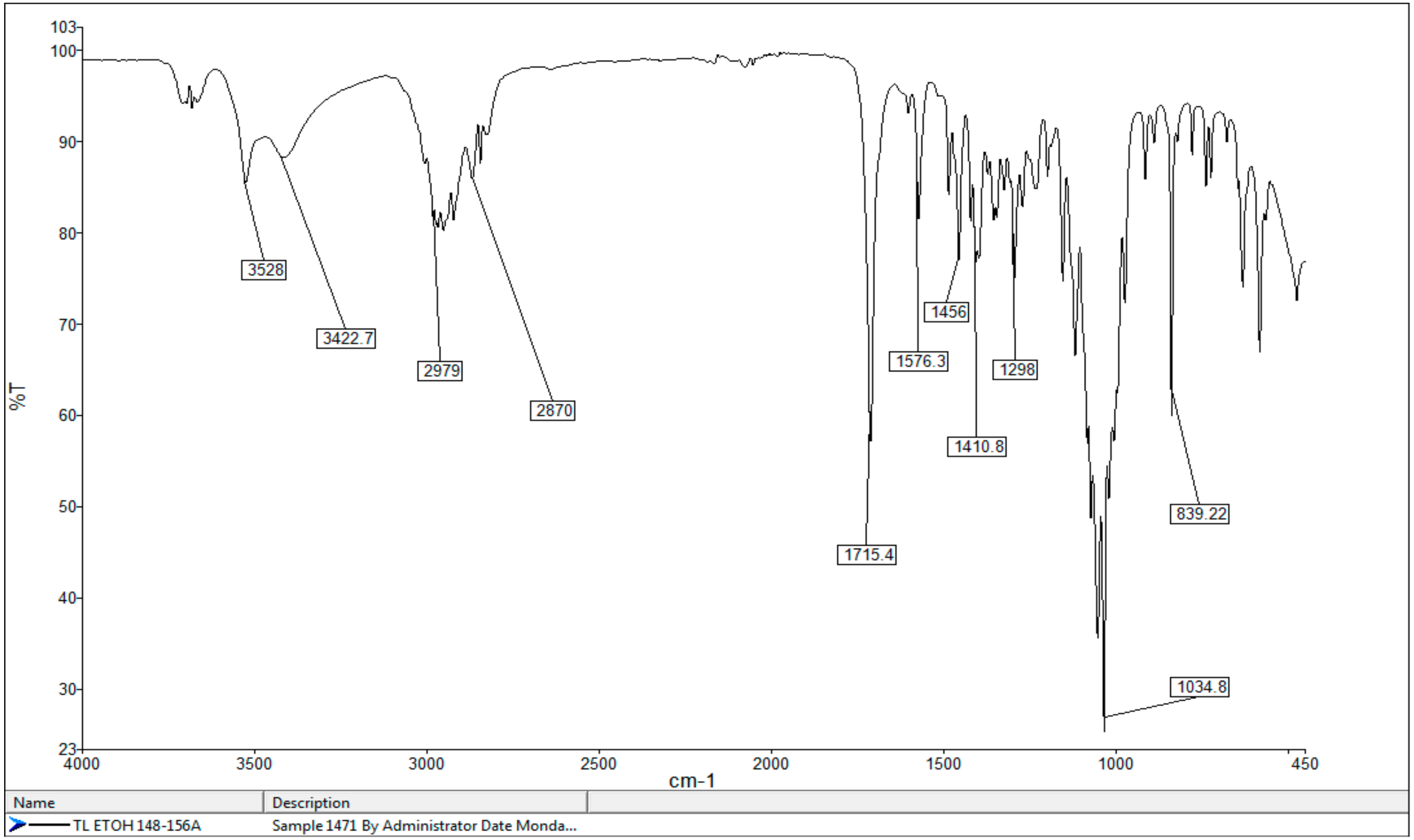

Figure SH.61: Fourier-Transform Infrared Spectroscopy (FTIR) spectrum of - Isofraxidin-7-O-β-D-glucopyranoside

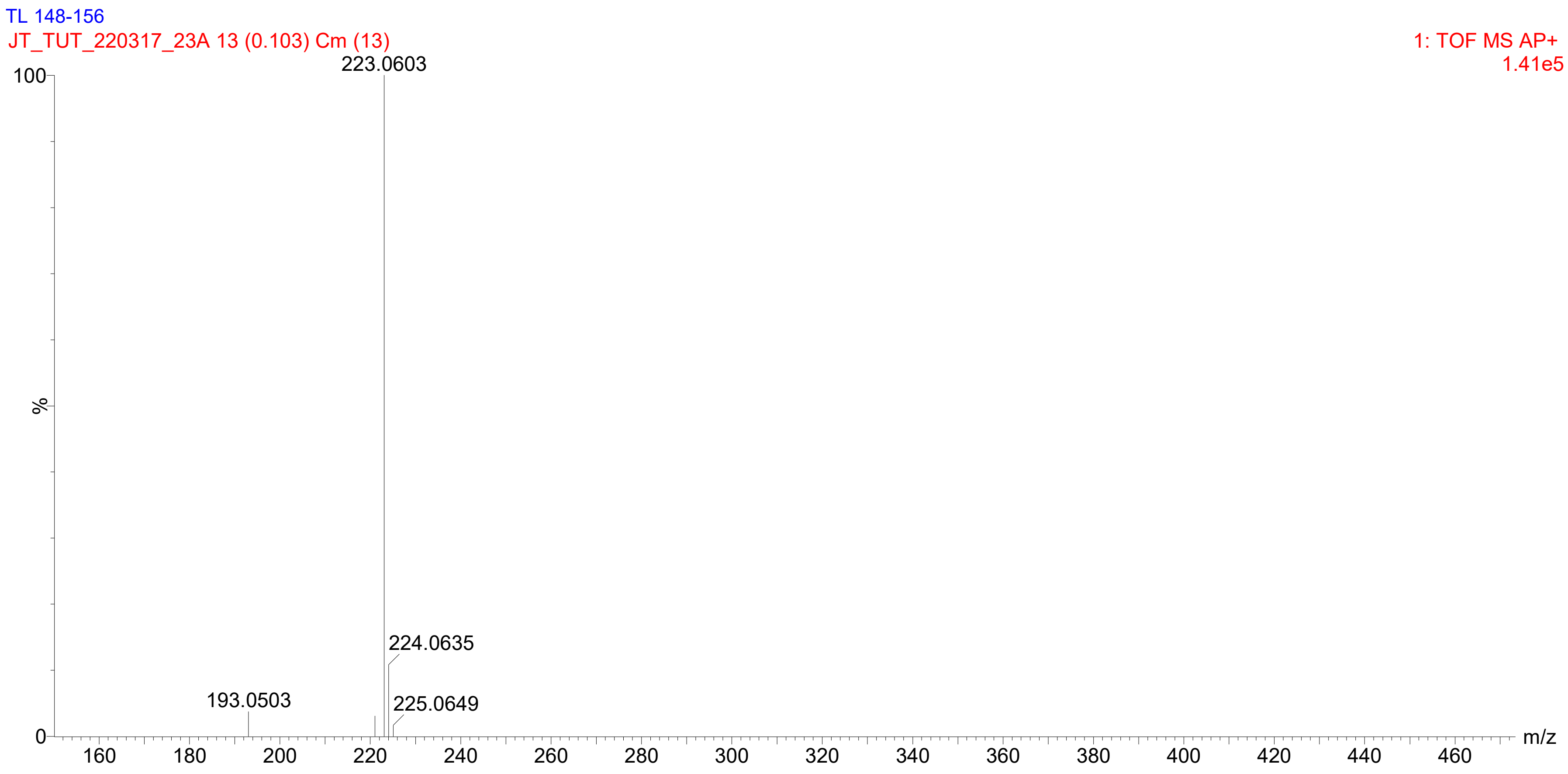

Figure SH.62: High-Resolution Electrospray Ionization Mass spectrum (HR-ESI-MS) of Isofraxidin-7-O-β-D-glucopyranoside [M-$C_6H_{12}O_6$] m/z = 223.0603

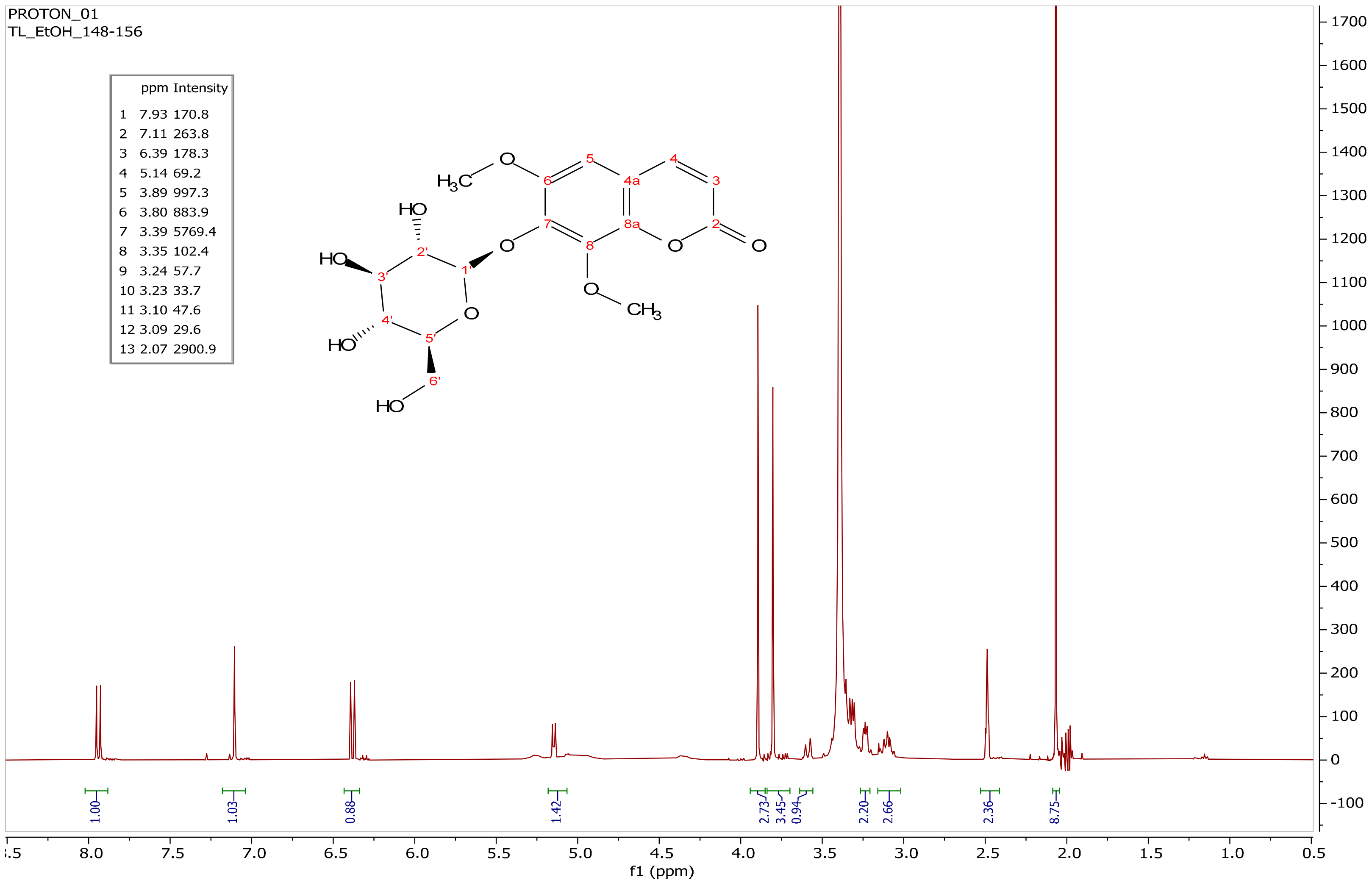

Figure SH.63: Proton Nuclear Magnetic Resonance (¹H NMR) spectrum of Isofraxidin-7-O-β-D-glucopyranoside (DMSO, 400 MHz)

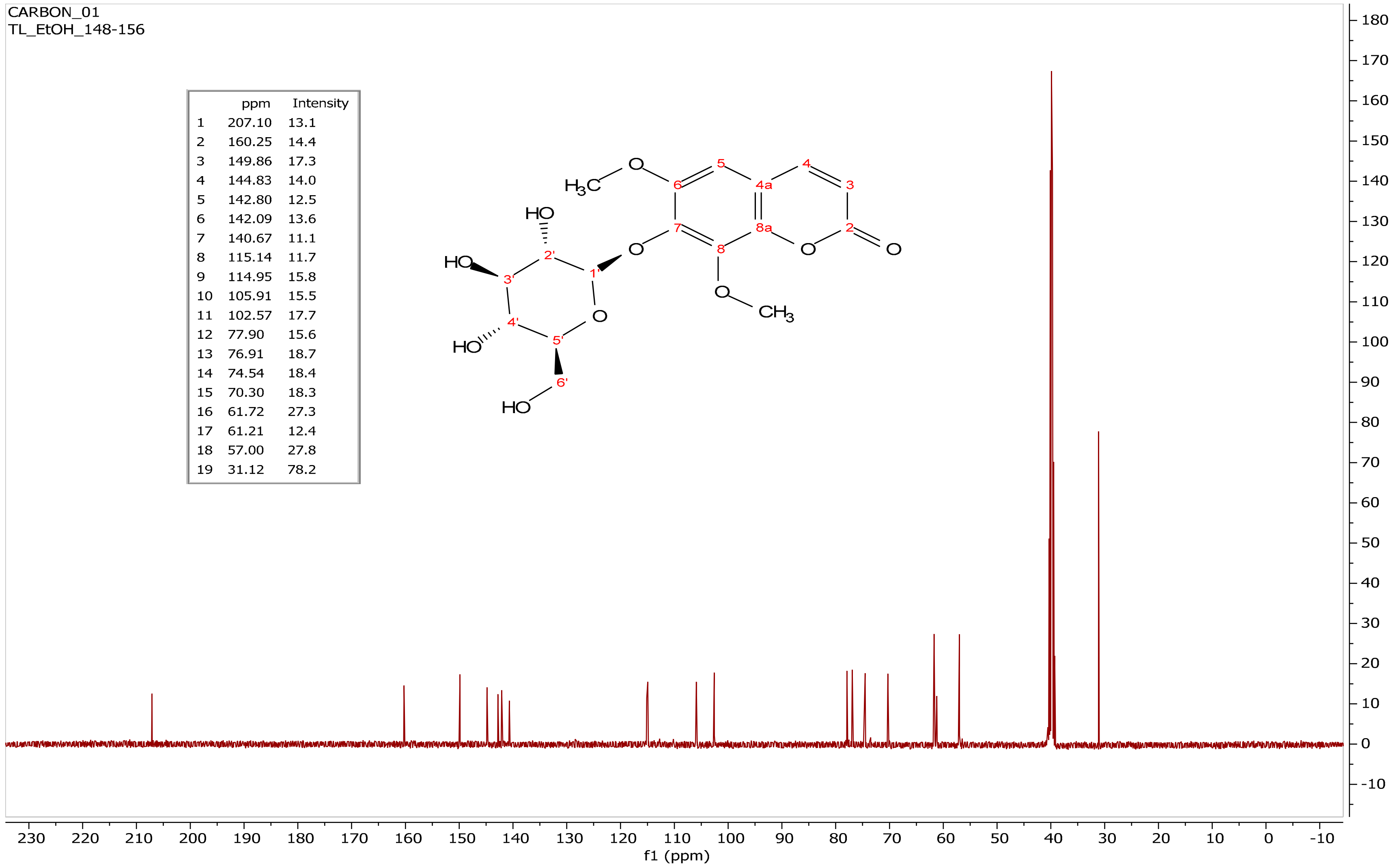

Figure SH.64: Carbon-13 Nuclear Magnetic Resonance (^{13}C NMR) spectrum of Isofraxidin-7-O-β-D-glucopyranoside (DMSO, 100 MHz)

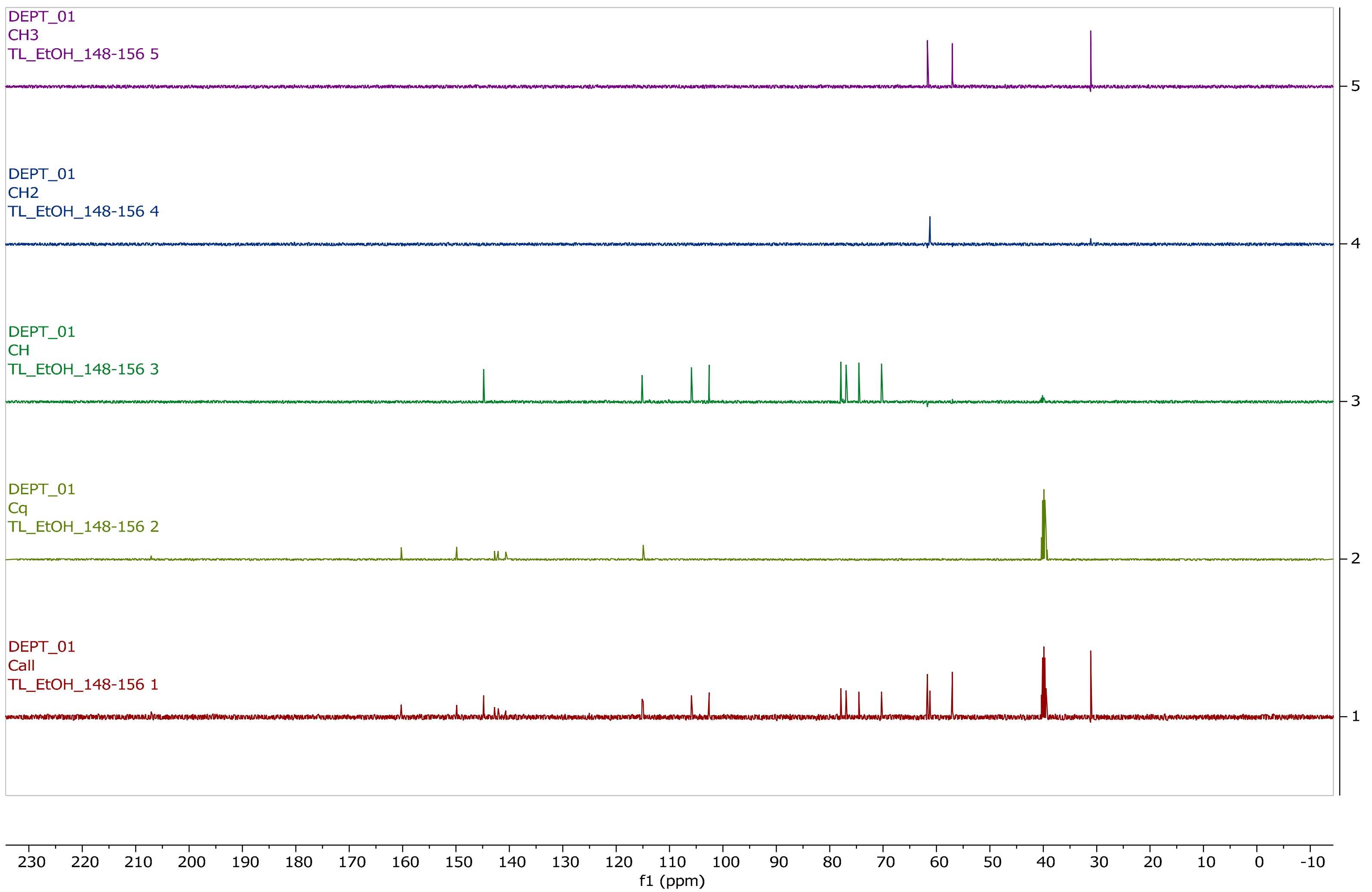

Figure SH.65: Distortionless Enhancement by Polarization Transfer (DEPT) NMR spectra Isofraxidin-7-O-β-D-glucopyranoside (DMSO, 100 MHz)

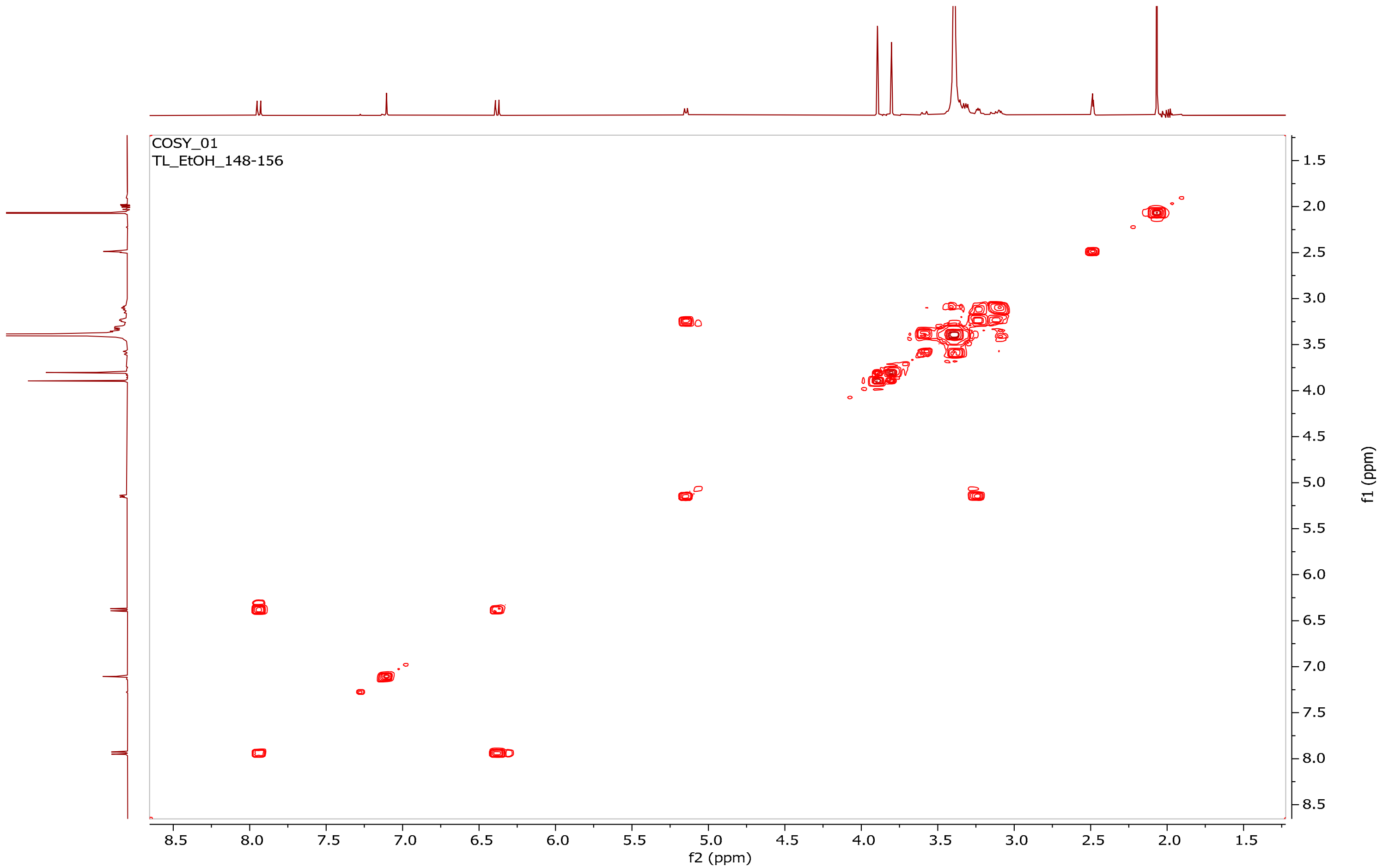

Figure SH.66: Gradient Correlated (gCOSY) spectrum of Isofraxidin-7-O-β-D-glucopyranoside

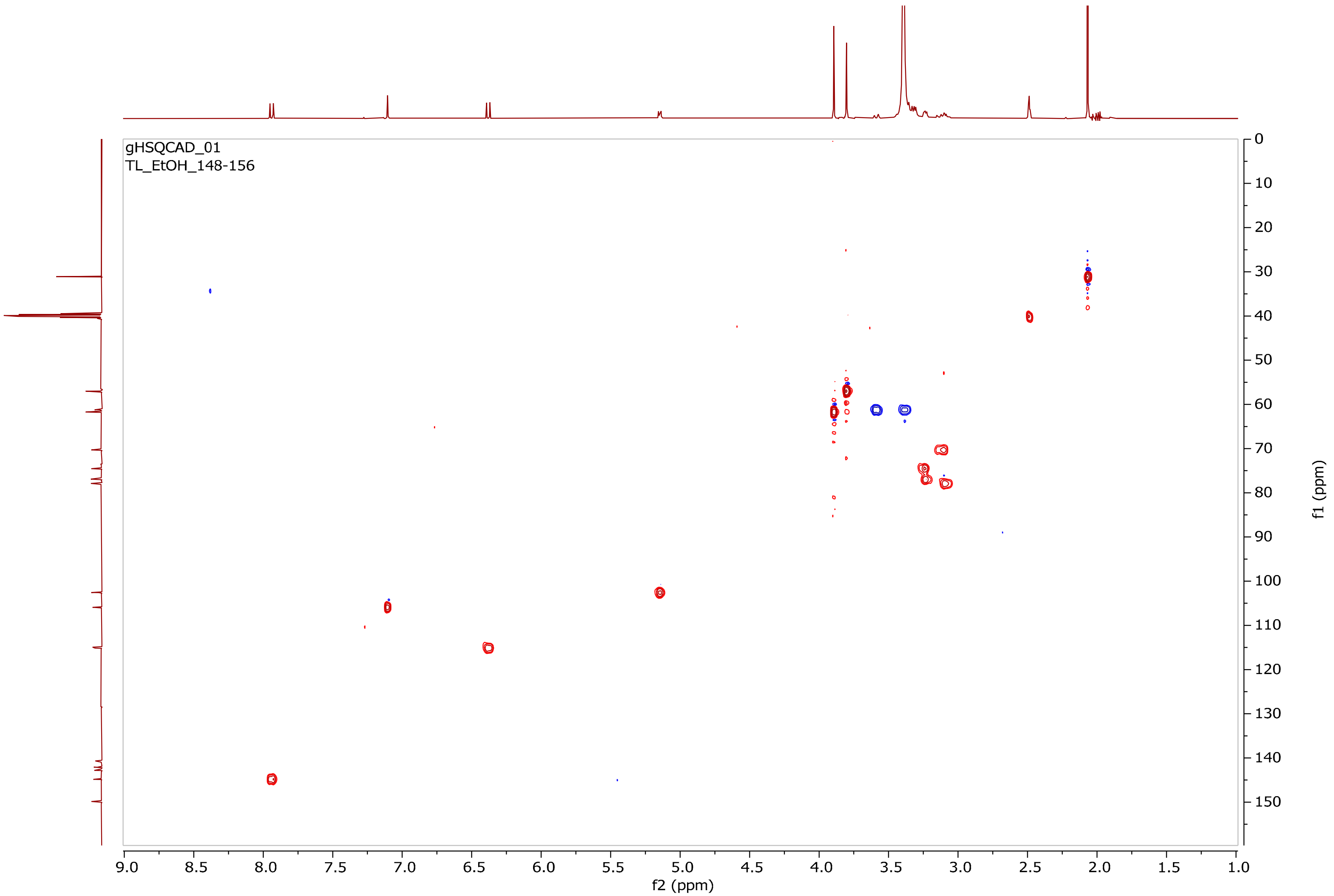

Figure SH.67: Gradient Heteronuclear Single Quantum Coherence (gHSQC) spectrum of Isofraxidin-7-O-β-D-glucopyranoside

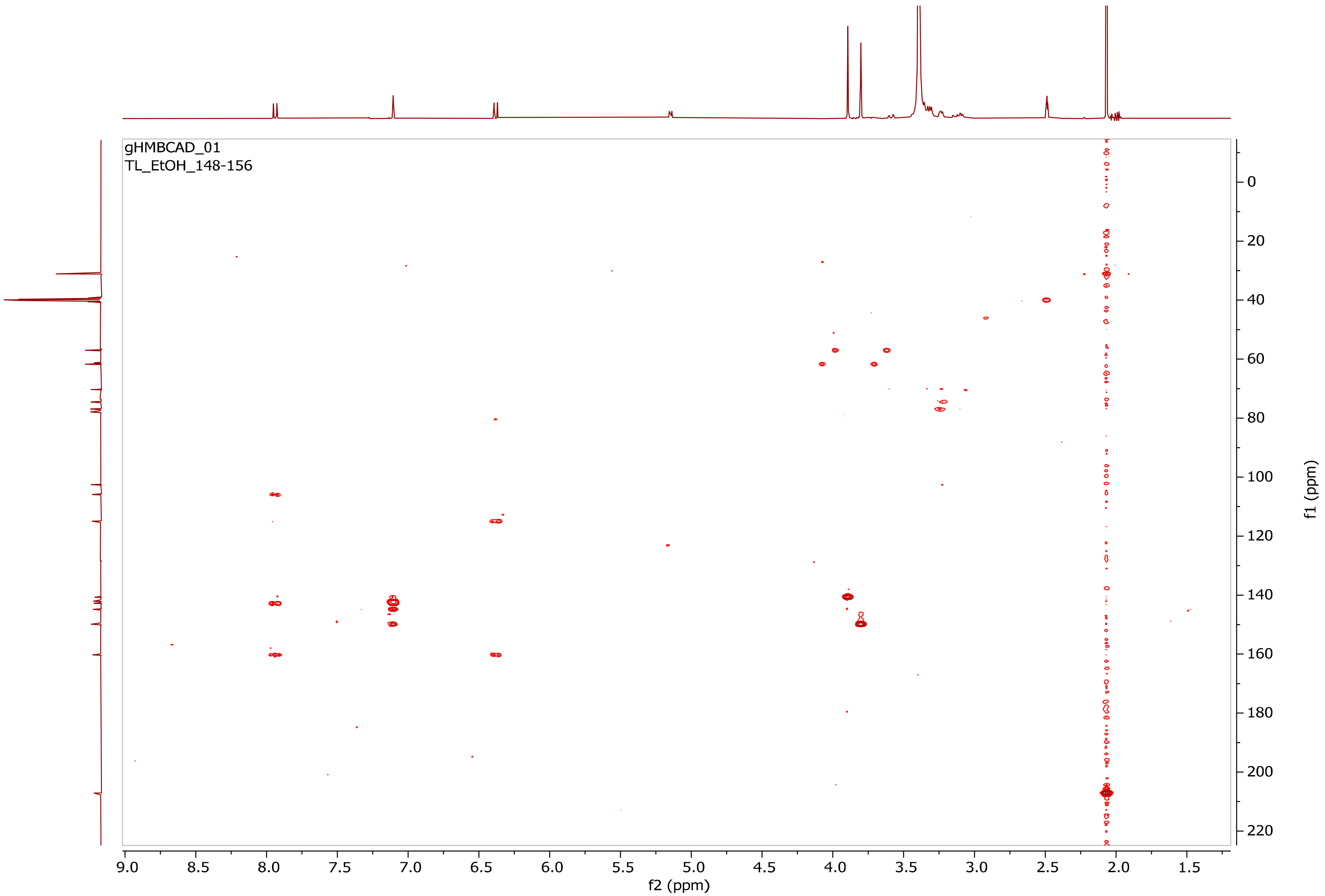

Figure SH.68: Gradient Heteronuclear Multiple Bond Quantum Coherence (gHMBC) spectrum of Isofraxidin-7-O-β-D-glucopyranoside
